# New Nitrogen-, Oxygen-, and Sulfur-Containing Heterocyclic Compounds as Anti-Colon Cancer Agents: Synthesis, Multitargeted Evaluations, Molecular Docking Simulations and ADMET Predictions

**DOI:** 10.3390/ph18060801

**Published:** 2025-05-27

**Authors:** Nahed Nasser Eid El-Sayed, Najeh Krayem, Hamed Ahmed Derbala, Shimaa Kamal, Syde Nasir Abbas Bukhari, Mohamed K. El-Ashrey, Zainab M. Almarhoon, Seham Soliman Alterary, Abir Ben Bacha

**Affiliations:** 1 Egyptian Drug Authority, 51 Wezaret El-Zerra St., Giza 35521, Egypt; 2 Laboratoire de Biochimie et de Génie Enzymatique des Lipases, ENIS, Université de Sfax, Route de Soukra 3038, Sfax BP 1173, Tunisia; krayemnajeh@yahoo.fr; 3 Chemistry Department, Faculty of Science, Ain Shams University, Abbassia, Cairo 11566, Egypt; hamed_derbala@sci.asu.edu.eg (H.A.D.); dr.shimo4189@yahoo.com (S.K.); 4 Department of Pharmaceutical Chemistry, College of Pharmacy, Jouf University, Sakaka 72388, Saudi Arabia; sbukhari@ju.edu.sa; 5 Pharmaceutical Chemistry Department, Faculty of Pharmacy, Cairo University, Kasr Elini St., Cairo 11562, Egypt; mohamed.elashrey@pharma.cu.edu.eg; 6 Medicinal Chemistry Department, Faculty of Pharmacy, King Salman International University, South Sinai 46612, Egypt; 7 Department of Chemistry, College of Science, King Saud University, P.O. Box 2455, Riyadh 11451, Saudi Arabia; zalmarhoon@ksu.edu.sa (Z.M.A.); salterary@ksu.edu.sa (S.S.A.); 8 Biochemistry Department, College of Sciences, King Saud University, P.O. Box 22452, Riyadh 11495, Saudi Arabia

**Keywords:** colorectal cancer, oxidative stress, Warburg effect, apoptosis, multitarget-directed ligands, heterocyclic compounds, ADMET

## Abstract

**Background/Objectives:** Oxidative stress, the Warburg effect, and resistance to apoptosis are key hallmarks driving colorectal tumorigenesis. This study aimed to develop novel multi-target compounds capable of modulating these pathways. **Methods:** A library of 24 newly synthesized compounds—incorporating annulated thiophene, thiazole, quinazolinone, 2-oxoindoline, and 1,2,3-oxadiazole scaffolds, as well as *N*-(1-(4-hydroxy-3-methoxyphenyl)-3-oxo-3-(2-(phenylcarbamothioyl)hydrazineyl) prop-1-en-2-yl)benzamide—was evaluated for antioxidant activity (DPPH assay), PDK-1 and LDHA inhibition, cytotoxic effects against LoVo and HCT-116 colon carcinoma cells, with parallel assessment of safety profiles on normal HUVECs. The underlying anticancer mechanism of the most active compound was investigated through analysis of cell cycle distribution, apoptosis induction, intracellular reactive oxygen species levels, mitochondrial membrane potential disruption, and expression levels of apoptosis-related genes. Molecular docking assessed binding interactions within LDHA and PDK-1 active sites. The physicochemical, drug-likeness, and ADMET properties of the multi-bioactive candidates were predicted in silico. **Results:** Among the synthesized compounds, thiophenes **3b** and **3d** exhibited potent PDK-1/LDHA and DPPH/LDHA inhibitions, along with significant cytotoxic effects on LoVo/HCT-116 cells (IC_50_ in µM: 190.30/170.21 and 156.60/160.96, respectively), while showing minimal cytotoxicity toward HUVECs. Molecular docking revealed favorable interactions with key amino acid residues within the LDHA and/or PDK-1 active sites. Compound **3d** notably induced G2/M (LoVo) and G1 (HCT-116) arrest and promoted apoptosis via enhancing ROS generation, modulating *Bax/Bcl-2* expressions, disrupting mitochondrial membrane potential, and ultimately activating *caspses-3.* In silico predictions indicated their promising drug-likeness and pharmacokinetics, though high lipophilicity, poor solubility (especially for **3b**), and potential toxicity risks were identified as limitations. **Conclusions:** Thiophenes **3b** and **3d** emerged as promising multi-target candidates; however, structural optimization is warranted to enhance their solubility, bioavailability, and safety to support further development as lead anti-colon cancer agents.

## 1. Introduction

Colorectal cancer (CRC) ranks as the third most commonly diagnosed malignancy in men and the second in women, and it remains the second-leading cause of cancer-related deaths worldwide [1]. Approximately 25% of patients are diagnosed at an advanced stage (stage IV), where the five-year survival rate drops below 14% [2]. Despite the availability of treatments such as surgery, chemotherapy, and targeted therapies, clinical outcomes remain suboptimal due to systemic toxicity, therapeutic resistance, poor long-term survival, and high recurrence rates [3]. These challenges highlight the need for novel strategies targeting the complex and multifactorial nature of CRC pathogenesis.

CRC arises from the dysregulation of multiple intracellular signaling pathways [4], enabling normal cells to acquire abnormal capabilities—or hallmarks—associated with malignancy [5].

One major contributor to CRC initiation, progression, and maintenance is oxidative stress (OS), a pathological condition marked by excessive production of reactive oxygen species (ROS) that overwhelm the cell’s antioxidant defenses. In intestinal mucosal cells, this ROS overproduction leads to oxidative DNA damage and promotes chronic inflammation [6], both of which contribute to malignant transformation. Moreover, OS supports tumor development through several mechanisms, including resistance to cell death, stimulation of proliferation, stress tolerance, angiogenesis, invasion, migration, and recurrence [7]. Accordingly, antioxidant compounds have garnered attention as potential chemopreventive agents against CRC [8].

CRC is also characterized by a distinct metabolic hallmark known as the Warburg effect, wherein cancer cells favor glycolysis over oxidative phosphorylation for ATP production—even under normoxic conditions [5]. This metabolic shift is driven largely by the upregulation of key glycolytic regulators such as lactate dehydrogenase A (LDHA) and pyruvate dehydrogenase kinase-1 (PDK-1) [9]. These enzymes facilitate the conversion of pyruvate to lactate, leading to promoting its accumulation. Lactate, an oncogenic metabolite, acidifies the tumor microenvironment, resulting in immune evasion, tumor progression, metastasis, and resistance to chemotherapy [10]. Consequently, impeding lactate production via the inhibition of LDHA and PDK-1 has emerged as a promising anticancer strategy [11].

Furthermore, CRC cells often harbor mutations in tumor suppressor genes such as *APC*, *TP53*, *KRAS*, and *CDKN2A* [5,12,13], which enable them to bypass cell cycle checkpoints, leading to uncontrolled proliferation [5]. In parallel, evasion of apoptosis is frequently observed, facilitated by dysregulation of pro-apoptotic (e.g., Bax, Bak) and anti-apoptotic (e.g., Bcl-xL, Bcl-2) proteins in the intrinsic pathway or by disruption of death-inducing signaling complexes in the extrinsic cascade [5,14]. This imbalance between proliferation and apoptosis plays a central role in tumor development, drives disease progression, and contributes to resistance to chemotherapy and radiotherapy [15]. As such, therapeutic approaches that simultaneously target cell cycle regulation and apoptotic pathways are being increasingly explored for effective CRC management [16].

Given these interconnected pathogenic features, the development of multitarget agents capable of modulating several cancer hallmarks represents a compelling strategy [17]. In this context, heterocyclic compounds containing nitrogen, oxygen, or sulfur have emerged as versatile scaffolds capable of modulating diverse cellular targets implicated in carcinogenesis [18,19,20,21,22,23].

In continuation of our previous efforts targeting CRC [24], we report the design and synthesis of four novel series of nitrogen-, oxygen-, and sulfur-containing five- and six-membered heterocycles as potential multitarget therapeutic agents. These compounds were evaluated for their antioxidant activity, inhibition of PDK-1 and LDHA, as well as their antiproliferative effects against HCT-116 and LoVo colon carcinoma cell lines. To assess selectivity and safety, cytotoxicity was also tested against normal human umbilical vein endothelial cells (HUVECs).

The lead antiproliferative compound was further investigated for its ability to disrupt cell cycle progression and induce apoptosis in CRC cells. Additionally, molecular docking simulations were performed to elucidate the interactions of active compounds with their enzymatic targets. Finally, in silico predictions of physicochemical properties, drug-likeness, ADME, and toxicity risks were conducted to assess the drug development potential of the lead multitarget candidates.

## 2. Results and Discussion

### 2.1. Chemistry

**Scheme 1:**
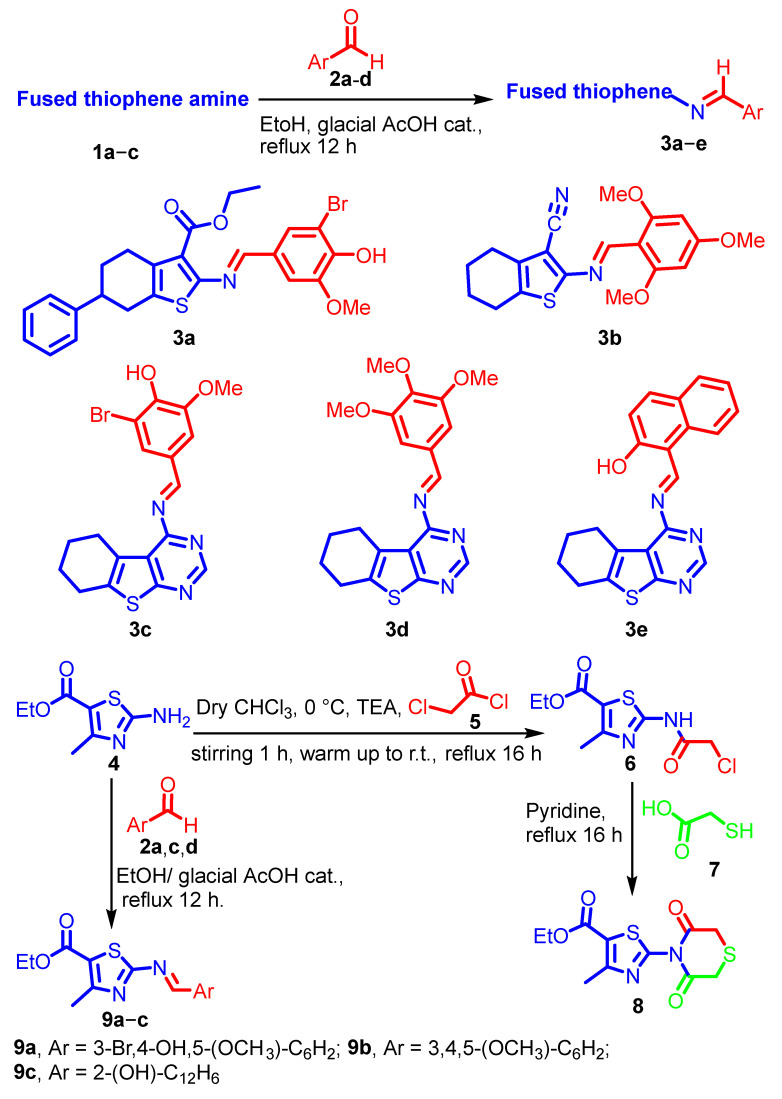
Synthesis of compounds **3a**–**e**, **8**, and **9a**–**c**.

**Scheme 2:**
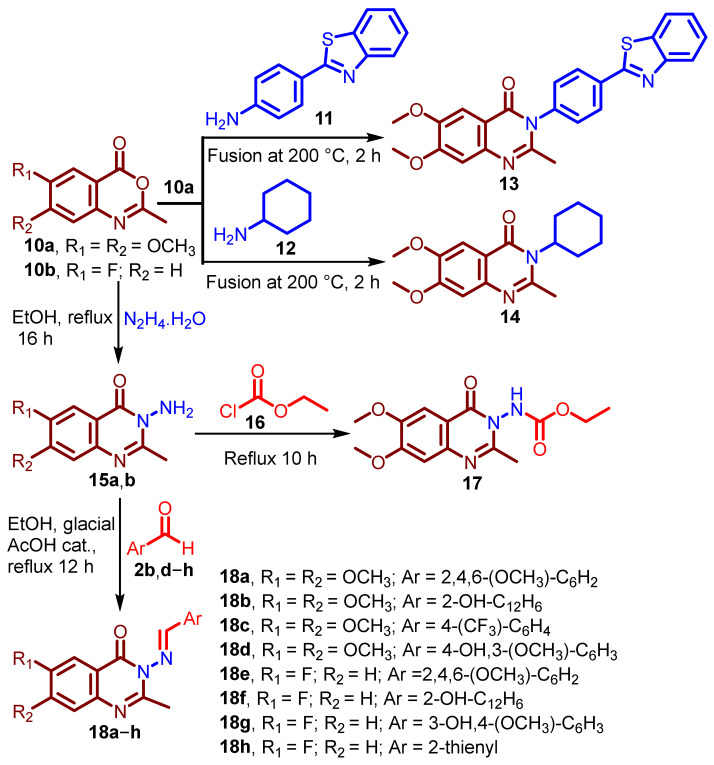
Synthesis of compounds **13**, **14**, **17**, and **18a**–**h**.

**Scheme 3:**
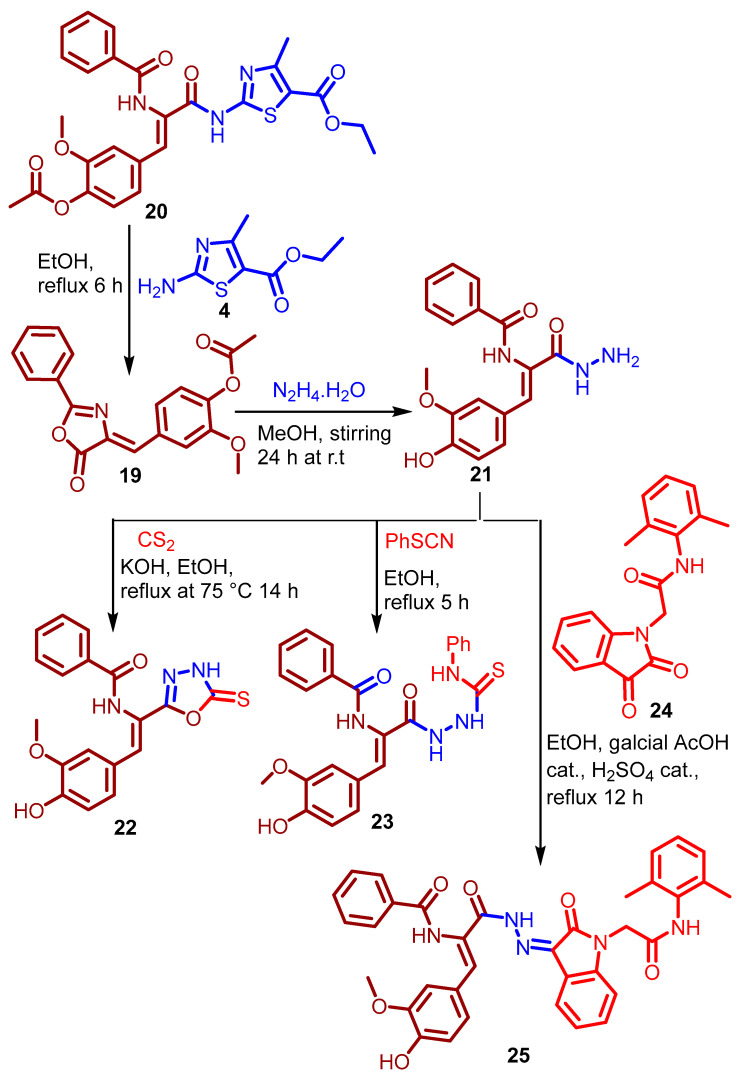
Synthesis of compounds **20**, **22**, **23**, and **25**.

The synthetic routes leading to the new target compounds **3a**–**e**, **8**, **9a**–**c**, **13**, **14**, **17**, **18a**–**h**, **20**, **22**, **23**, and **25** are depicted in Scheme 1, Scheme 2 and Scheme 3. The starting heterocyclic compounds used in this study—including 2-aminothiophenes **1a**–**c** [25,26,27], 2-aminothiazole **4** [28], 4-(benzo[*d*]thiazol-2-yl)aniline **11** [29], oxazinones **10a**,**b** and the corresponding 3-aminoquinazolinones **15a**,**b** [30,31], as well as azlactone **19** and its hydrazide derivative **23** [32]—were synthesized according to previously reported procedures.

Initially, equimolar amounts of the appropriate fused thiophene-based amines **1a**–**c** and aromatic aldehydes **2a**–**d** were heated under reflux in absolute ethanol containing a catalytic amount of glacial acetic acid for 12 h, yielding the thiophene–Schiff base-conjugates **3a**–**e** (Scheme 1). Meanwhile, the reaction of 2-aminothiazole **4** with chloroacetyl chloride **5** in dry chloroform and in the presence of Et_3_N yielded chloroacetamide intermediate **6**, which subsequently condensed with thioglycolic acid **7** in pyridine to furnish ethyl 2-(3,5-dioxothiomorpholino)-4-methylthiazole-5-carboxylate **8**. Additionally, the condensation of amine **4** with aromatic aldehydes **2a**, **2c,** and **2d** in absolute ethanol containing a catalytic amount of glacial acetic acid afforded Schiff bases **9a**–**c** (Scheme 1).

The fusion of oxazinone **10a** with 4-(benzo[*d*]thiazol-2-yl)aniline **11** or cyclohexyl amine **12** led to the formation of 3-substituted-6,7-dimethoxy-2-methyl quinazolin-4(3*H*)-one derivatives **13** and **14**, respectively. Also, the reaction of oxazinones **10a**,**b** with excess hydrazine hydrate afforded the corresponding 3-aminoquinazolinones **15a**,**b**. Next, refluxing 3-aminoquinazolinone **15a** with excess ethyl chloroformate **16** yielded the carbamate derivative **17**, and eight new Schiff bases, **18a**–**h**, were synthesized through the condensation of 3-aminoquinazolinones **15a**,**b** with the appropriate aryl and/or heteroaryl aldehydes **2b**,**2d**–**h** in absolute ethanol containing a catalytic amount of glacial acetic acid (Scheme 2).

In a separate sequence (Scheme 3), (*Z*)-2-methoxy-4-((5-oxo-2-phenyloxazol-4(5*H*)-ylidene)methyl) phenyl acetate **19** underwent ring opening upon reaction with ethyl 2-amino-4-methylthiazole-5-carboxylate **4**, affording the corresponding thiazolyl benzamidoacrylamide **20**. Meanwhile, treatment of compound **19** with excess hydrazine hydrate at room temperature led to ring opening and deacylation of the ester group, yielding hydrazide **21**. Cyclocondensation of the latter compound with carbon disulfide in the presence of KOH afforded 2-thioxo-1,3,4-oxadiazole derivative **22**. Furthermore, hydrazide **21** was converted into the *N*-phenylthiosemicarbazide derivative **23** through reaction with phenyl isothiocyanate. Finally, condensation of hydrazide **21** with isatin derivative **24** produced 2-oxoindolyl hydrazone **25** (Scheme 3).

The structures of all newly synthesized compounds were confirmed by FT-IR, ^1^H and ^13^C-NMR and MS analyses as detailed in Section 3; representative spectra are provided in the Supplementary Materials.

Notably, due to the restricted rotation around the imine (C=N) group, two geometric isomers—*E* and *Z*—are theoretically possible for the aldimines **3a**–**e**, **9a**–**c**, and **18a**–**h**. However, these compounds were obtained as single geometrical isomers, evidenced by their ^1^H-NMR spectra. Additionally, energy minimization and total energy calculations (Table S1) revealed that the *Z*-isomers possessed higher total energies and were therefore less stable than their *E* counterparts. This reduced stability is likely due to steric repulsion between the bulky heterocyclic and aryl groups across the C=N bond [33], thus favoring the *E*-configuration for these aldimines.

Considering compounds **20**, **21**, **22**, **23**, and **25**, the configuration of the olefinic double bond within the conjugated side chains was assigned as *Z*, consistent with the geometry of the parent 4-arylidene-2-phenyloxazol-5(4*H*)-one [34].

In addition, the geometry around the double bond of the 2-oxoindolin-3-ylidene)hydrazineyl fragment in compound **25** was assigned a *Z*-configuration based on previous studies of related compounds, which reported that the hydrazide-NH and the 2-oxoindoline carbonyl group form an intramolecular hydrogen bond that enhances stabilization [35]. This assignment is further supported by energy minimization data, which indicate that the *Z*,*Z*-isomer possesses lower energy than the *Z*,*E*-isomer (Table S1).

### 2.2. In Vitro Biological Evaluations

The results of various biochemical assays for the newly synthesized compounds, **3a**–**e**, **8**, **9a**–**c**, **13**, **14**, **17**, **18a**–**h**, **20**, **22**, **23**, and **25**, are summarized in Tables S2–S6.

Notably, in the enzyme inhibition and viability assays, the initial screening concentration of 100 µg/mL was selected based on our standard practice in early-stage multitarget drug discovery [24]. This approach facilitates the identification of hit compounds by enabling rapid and consistent comparison across structurally diverse candidates prior to detailed dose-response analysis.

#### 2.2.1. Antioxidant Activity

The antioxidant activities of the studied compounds were assessed using the 1,1-diphenyl-2-picrylhydrazyl (DPPH) radical scavenging method as detailed by Bersuder et al. [36]. The results were expressed as IC_50_ (mM)—the concentration of the test compound required to neutralize 50% of DPPH radicals. As shown in Table S2, the compounds exhibited variable degrees of scavenging power, with IC_50_ values spanning from 0.164 ± 0.008 to 1.811 ± 0.115 as compared to 0.245 ± 0.027 mM for the reference antioxidant, butylated hydroxytoluene (BHT). In detail, the thiophenes **3a**–**e**, thiazoles **8** and **9a**–**c**, and quinazolinones **13**, **14**, **17**, and **18a**–**h**, along with benzamide derivatives **20**, **22**, **23**, and **25**, demonstrated IC_50_ values ranging from 0.164 ± 0.008 to 0.890 ± 0.039, 0.881 ± 0.041 to 1.811 ± 0.115, 0.188 ± 0.010 to 1.820 ± 0.188 and 0.162 ± 0.023 to 1.211 ± 0.030 mM, respectively. Among them, compounds **3a**, **3d**, **18b**, and **18g** demonstrated better DPPH radical scavenging activity than BHT, with lower IC_50_ values of 0.214 ± 0.027, 0.164 ± 0.008, 0.188 ± 0.010, and 0.238 ± 0.006 mM, respectively. In contrast, none of the thiazole-based derivatives **8**, **9a**–**c **and **20** exhibited significant antioxidant activity, despite the fact that the Schiff bases **9a**–**c** were derived from the same aldehydes as the most potent scavengers **3a**, **3d** and **18b**, respectively.

#### 2.2.2. PDK-1 and LDHA Inhibitory Activities

The same set of compounds was initially screened for their inhibitory effects on PDK-1 and LDHA at a concentration of 100 µg/mL, and the results are listed in Table S3.

In the PDK-1 assay, thiophene **3b** along with quinazolinones **14** and **17** showed the highest inhibition values of 94.65 ± 2.33, 80.50 ± 4.95, and 91.00 ± 2.83%, respectively, compared to 100% inhibition by the reference compound sodium dichloroacetate (SDA) at 150.92 µg/mL. The rest of the compounds exhibited weak to moderate inhibition, ranging from 7.60 ± 0.85 to 72.67 ± 2.08%.

Similarly, in the LDHA assay, thiophenes **3b** and **3d** were the most potent inhibitors, with inhibition rates of 82.75 ± 3.18 and 85.90 ± 2.97, respectively, compared to 100% inhibition achieved by the standard inhibitor sodium oxamate (SO) at 111.03 µg/mL. The rest of the compounds showed inhibition ranging from 5.75 ± 0.35 to 67.35 ± 3.32%.

For compounds showing >80% inhibition of either enzyme, IC_50_ values (µM) were determined (Table S3). Thus, IC_50_ values against PDK-1 for compounds **3b**, **3d**, **14**, and **17** were 147.29 ± 5.95, 305.11 ± 11.07, 192.08 ± 5.57, and 168.86 ± 1.81, respectively, compared to 170.62 ± 7.03 µM for SDA. Against LDHA, their IC_50_ (µM) values were 166.79 ± 10.11, 149.03 ± 7.93, 378.01 ± 3.98, and 1059.71 ± 7.64, respectively, compared to 140.50 ± 7.64 for SO.

Overall, among all the studied compounds, thiophene derivative **3b** was identified as the most promising dual inhibitor of PDK-1 and LDHA. Although thiophene **3d** exhibited weak PDK-1 inhibition, it was the most potent LDHA inhibitor. These findings are notable, as several studies highlighted a strong correlation between PDK-1 inhibition and the reduction of cellular proliferative tumorigenic potential as well as enhanced radiosensitization in various tumor cell lines [37,38]. Similarly, previous in vitro and in vivo xenograft models and preclinical studies have shown that the reduction of LDHA expression and inhibition of its activity in cancer cells stimulates mitochondrial OXPHOS, leading to increased mitochondrial ROS production, which in turn triggers apoptosis-mediated cell death [39,40] and enhances the efficacy of radiotherapy [41].

#### 2.2.3. Cytotoxic Activities

The studied compounds were also screened for their cytotoxic effects on HCT-116 and LoVo colon carcinoma cells, as well as normal HUVECs, using the standard LDH assay [42] at a concentration of 100 µg/mL. Results are reported as the mean percentage of viable cells after 48 h of treatment (Table S4). Notably, compounds **3b**, **3d**, **8**, **14**, **17**, **18d**, **18h**, and **22** induced significant cytotoxicity in LoVo cells, reducing cell viability to 28.33 ± 1.41, 19.67 ± 2.83, 13.50 ± 0.71, 13.00 ± 1.14, 10.50 ± 0.71, 30.67 ± 4.24, 33.00 ± 4.24, and 29.50 ± 2.12%, respectively, compared to 99.50 ± 0.71% (assay medium, negative control) and 0% (0.1% Triton X-100, positive control). Against HCT-116 cells, compounds **3b**, **3d**, **8**, **14**, **17**, **18a**, **18d**, **18h**, **22** and **23** reduced cell viability to 20.67 ± 2.08, 21.67 ± 2.08, 26.50 ± 2.12, 12.50 ± 0.71, 20.50 ± 2.12, 40.67 ± 4.16, 38.00 ± 2.00, 37.00 ± 2.00, 40.50 ± 3.54 and 23.00 ± 2.83%, respectively, compared to 100.00% (negative control) and 0% (positive control). The remaining compounds exhibited moderate to weak cytotoxic activities, with viabilities ranging from 48.33 ± 2.83 to 95.67 ± 1.53% (LoVo cells) and 49.00 ± 2.83 to 89.33 ± 4.16% (HCT-116 cells). Notably, all tested compounds showed limited cytotoxicity toward normal HUVECs, with cell viabilities ranging from 96.67 ± 1.16 to 70.33 ± 1.53%.

Based on these results, the mean IC_50_ values (µM) were determined for the ten most active compounds (i.e., those reducing viability to <41.00% against either cell line) and are presented in Table S5. These compounds demonstrated variable sensitivity, with IC_50_ values spanning from 132.23 ± 7.40 to 479.17 ± 19.18 µM (LoVo) and from 160.96 ± 5.43 to 510.84 ± 5.61 µM (HCT-116), compared to 19.99 ± 3.21 and 26.98 ± 1.87 µM, respectively, for 5-fluorouracil. Among all the tested compounds, quinazolinone **18a** was the most effective against LoVo cells (IC_50_ = 132.23 ± 7.40 µM), while thiophene **3d** was the second most active compound against LoVo cells (IC_50_ = 156.60 ± 5.22 µM) and the most potent against HCT-116 (IC_50_ = 160.96 ± 5.43 µM).

#### 2.2.4. Investigation of Anticancer Mechanism of **3d**

Cell cycle regulation and apoptosis are closely interconnected processes that play critical roles in carcinogenesis. Disruption of genomic integrity through severe or irreparable DNA damage can lead to the unchecked proliferation of abnormal cells, thereby contributing to tumor development. Conversely, the activation of cell cycle checkpoints and arrest, along with the induction of apoptosis, functions as a crucial negative regulatory mechanism to eliminate damaged cells and prevent malignant transformation [43]. Accordingly, these mechanisms are key targets in cancer therapy and active areas of research, leading to the identification of many chemotherapeutic agents and candidates, whose anticancer effects rely on inhibiting cell proliferation, inducing cell cycle arrest, and/or promoting apoptotic cell death [15,16,17]. On this basis, and owing to its significant cytotoxic activity against both LoVo and HCT-116 colon cancer cell lines, compound **3d** was selected for further mechanistic investigation to elucidate its mode of action by evaluating its ability to induce cell cycle arrest and/or trigger apoptosis.

##### Effect of Compound **3d** on Cell Cycle Progression

**Figure 1:**
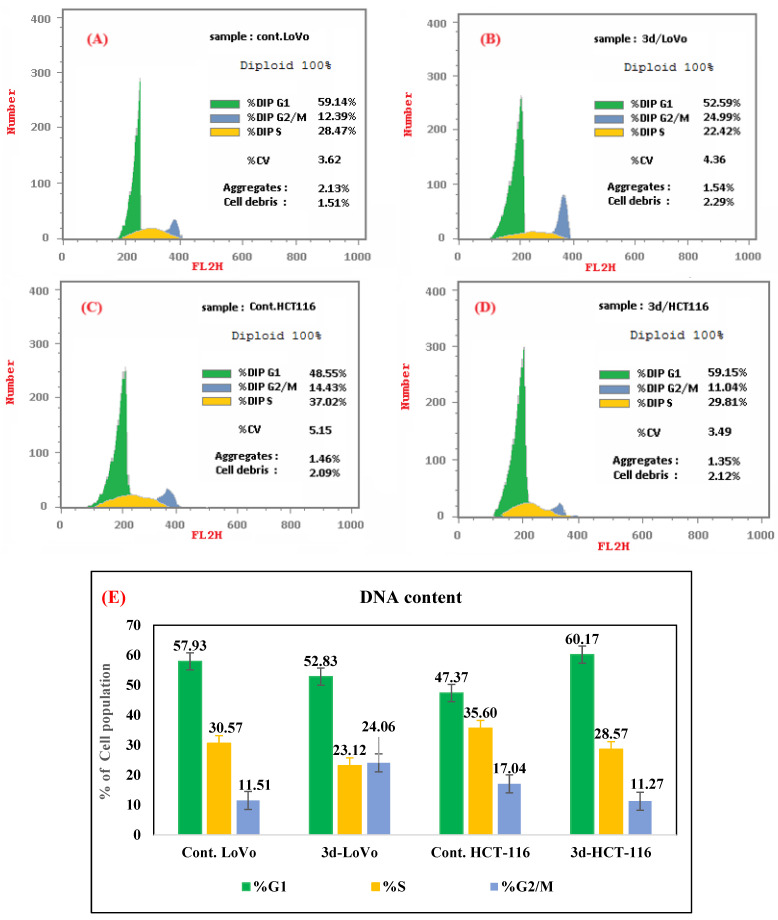
Effects of compound **3d** on the cell cycle distribution of LoVo and HCT-116 cells: (**A**) negative control LoVo cells, (**B**) **3d**-treated LoVo cells, (**C**) negative control HCT-116 cells, (**D**) **3d**-treated HCT-116 cells, (**E**) bar chart depicting mean cell cycle distribution (% ± SD, *n* = 2).

Cell proliferation depends on orderly progression through the phases of the cell cycle (G1−S−G2−M). Cytotoxic agents can impair the integrity of DNA, leading to checkpoint arrest at G1/S or G2/M, which subsequently blocks proliferation [43]. To evaluate the impact of compound **3d** on cell cycle distribution, colon carcinoma cells were treated with 30 µg/mL of **3d** for 48 h, followed by DNA content analysis using flow cytometry. As shown in DNA histograms (Figure 1A–D; representative example) and the bar chart summarizing the results of two replicates (Figure 1E; mean% ± SD, n = 2), treatment with **3d** increased the proportion of LoVo cells in the G2/M phase to 24.06 ± 1.32%, compared to 11.51 ± 1.24% in untreated control cells. In contrast, in HCT-116 cells, **3d** caused an accumulation in the G1 phase, reaching 60.17 ± 1.44% versus 47.37 ± 1.68% in untreated cells (Figure 1E). These results suggest that compound **3d** inhibits proliferation through the induction of G2/M arrest in LoVo cells and G1 arrest in HCT-116 cells.

##### Induction of Apoptosis by Compound **3d**

Apoptosis is a tightly regulated, multistep process orchestrated through two main signaling pathways: the intrinsic (mitochondrial) and extrinsic (death receptor-mediated) [44,45]. Both pathways are tightly regulated by molecular checkpoints, most notably the B-cell lymphoma 2 (BCL-2) protein family and caspases. The B-cell lymphoma 2 (BCL-2) family of proteins comprises pro-apoptotic (e.g., Bax, Bak, and Bok) and anti-apoptotic (e.g., Bcl-2 and Bcl-xL) members [46]. The second pivotal regulatory family, caspases—cysteine-aspartic proteases—are broadly categorized into initiator caspases (Casp-2, -8, -9, and -10) and executioner caspases (Casp-3, -6, and -7) [47]. It is well established that cancer cells have a compromised antioxidant defense system and require higher levels of reactive oxygen species (ROS) to promote mitogenic effects and cellular proliferation compared to normal cells [48]. However, when ROS accumulated beyond a critical threshold, oxidative stress arises—a key initiator of both apoptotic cell mechanisms. 

In the intrinsic pathway, ROS levels are augmented in mitochondria (due to external agents such as cytotoxic agents and UV radiation). This disrupts cellular redox homeostasis and damages mitochondrial components, which, in turn, promotes the activation of pro-apoptotic BCL-2 family proteins such as Bax. Activated Bax induces mitochondrial outer membrane permeabilization (MOMP), facilitating the release of apoptogenic factors like cytochrome c and apoptosis-inducing factor (AIF) into the cytosol. Cytochrome c then associates with apoptotic protease-activating factor 1 (Apaf-1) to activate initiator Casp-9, which subsequently activates executioner Casp-3. Activated Casp-3 cleaves cellular substrates, driving the morphological and biochemical hallmarks of apoptosis [45]. Conversely, anti-apoptotic proteins such as Bcl-2 can preserve mitochondrial integrity to inhibit this cascade [49]. Therefore, the Bax/Bcl-2 ratio is a critical determinant of mitochondrial-mediated apoptosis. 

In the extrinsic pathway, ROS modulates signaling molecules involved in this cascade [3,50], thereby enhancing the expression or activation of death receptors and promoting the binding of extracellular death ligands to their receptors on the cell surface, forming the death-inducing signaling complex (DISC). This complex activates initiators Casp-8 and -10, which regulate the expression of executioners Casp-3, -6 and -7. Therefore, both apoptosis pathways converge at Casp-3, the central executioner caspase.

In light of these findings, to elucidate whether the cytotoxic activity of compound **3d** is associated with apoptosis induction, we investigated multiple apoptotic markers [51,52].

- ROS Generation as an Upstream Apoptotic Trigger

To determine whether compound **3d** induces oxidative stress, intracellular ROS levels were quantified after treatment (30 µg/mL, 48 h) using a human ROS ELISA kit. As shown in Table S6, compound **3d** significantly increased ROS levels to 434.5 pg/mL in LoVo cells and 290.1 pg/mL in HCT-116 cells, compared to 168.6 pg/mL and 143.5 pg/mL in the respective controls. These results suggest that ROS accumulation serves as a likely upstream trigger of apoptosis.

- Modulation of Apoptosis-Related Gene Expression

**Figure 2:**
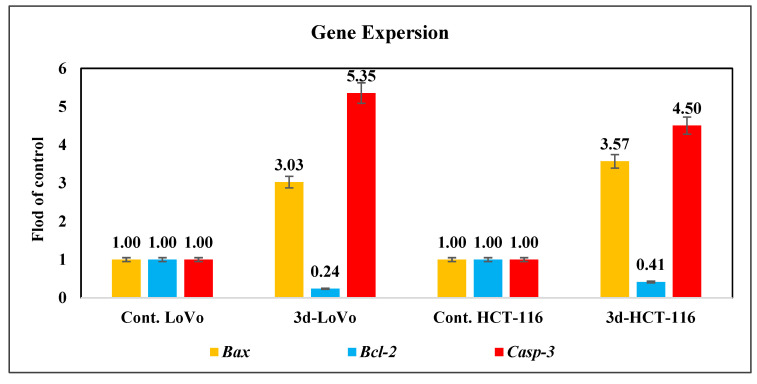
qRT-PCR quantification of *Bax*, *Bcl-2* and *Casp-3* genes expression in LoVo and HCT-116 cells treated with compound **3d** (30 µg/mL, 48 h). Data are expressed as fold changes relative to untreated controls and normalized to *GAPDH*.

To investigate molecular events downstream of ROS generation, we evaluated mRNA expression levels of key regulators of mitochondrial apoptosis—*Bax* and *Bcl-2*—using qRT-PCR. This analysis revealed that compound **3d** significantly upregulated *Bax* mRNA levels by 3.03 ± 0.41-fold in LoVo cells and 3.57 ± 0.18-fold in HCT-116 cells. Simultaneously, *Bcl-2* expression was significantly downregulated to 0.24 ± 0.03-fold and 0.41 ± 0.04-fold, respectively (Figure 2). As a result, the *Bax*/*Bcl-2* ratios were calculated to be 12.63 in LoVo cells and 8.71 in HCT-116 cells, reflecting a pronounced pro-apoptotic shift. This alteration in the *Bax*/*Bcl-2* ratio strongly supports the activation of the mitochondrial apoptotic pathway.

- Loss of Mitochondrial Membrane Potential (ΔΨm)

To assess mitochondrial integrity, TMRE staining was used to measure ΔΨm. Treatment with compound **3d** (30 µg/mL, 48 h) significantly reduced ΔΨm by 60.23 ± 1.87% in LoVo cells and 43.47 ± 0.81% in HCT-116 cells, supporting mitochondrial depolarization as a key mechanistic event consistent with the observed increase in ROS levels.

- Upregulation of Caspase-3 Expression

To confirm activation of the execution phase of apoptosis, *Casp-3* mRNA levels were evaluated by qRT-PCR. Treatment with compound **3d** significantly upregulated *Casp-3* expression by 5.35 ± 0.33-fold in LoVo cells and 4.50 ± 0.40-fold in HCT-116 cells (Figure 2), verifying the engagement of caspase-mediated apoptotic execution.

- Phosphatidylserine Externalization by Annexin V-FITC/PI Staining

Translocation of phosphatidylserine (PS) residues on the cell surface is an early apoptosis indicator, detectable through flow cytometric analysis and using double staining with propidium iodide (PI) dye and fluorescein isothiocyanate (FITC)-conjugated Annexin V (Annexin V-FITC) [53].

**Figure 3:**
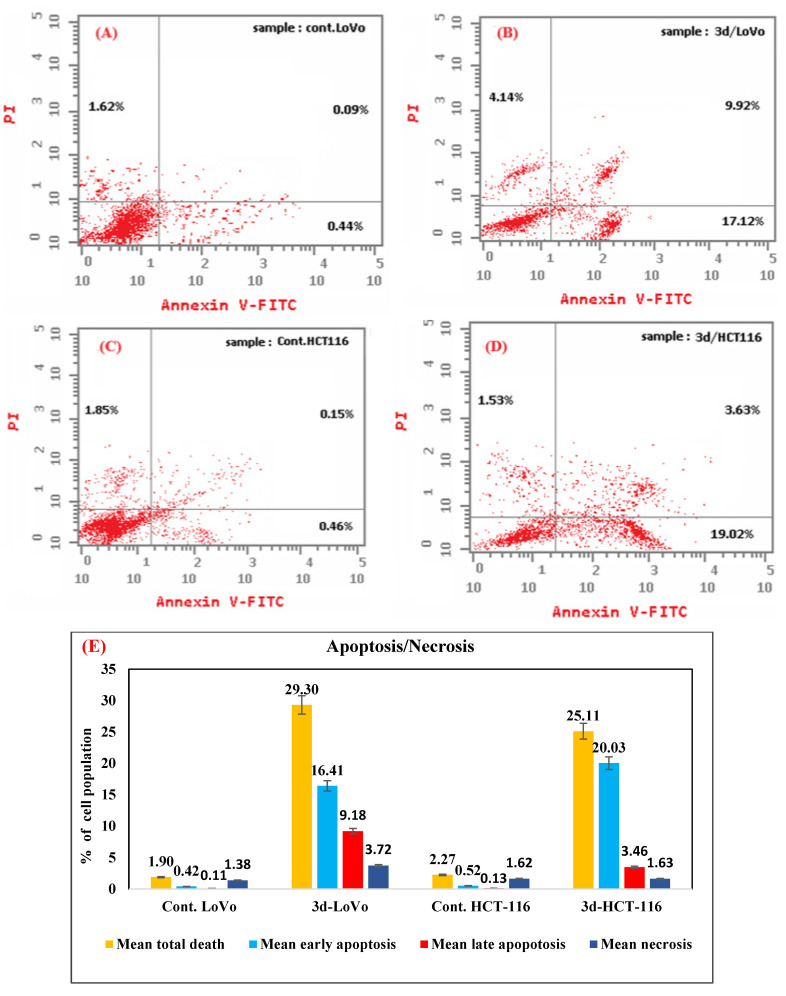
Flow cytometric analysis of apoptosis in LoVo and HCT-116 cells treated with compound **3d** (30 µg/mL, 48 h): (**A**) untreated LoVo cells (control), (**B**) **3d**-treated LoVo cells, (**C**) untreated HCT-116 cells (control), (**D**) **3d**-treated HCT-116 cells. (**E**) bar chart quantifying early apoptosis, late apoptosis, total apoptosis, and necrosis expressed as mean percentages ± SD of two independent experiments.

The results of this assay (Figure 3A–E) revealed increased apoptotic populations. In LoVo cells, early (Annexin V-FITC positive and PI negative) and late apoptotic cells (both Annexin-V and PI positive) increased to 17.12% and 9.92%, respectively, compared to 0.44% and 0.09% in the controls (Figure 3B vs. Figure 3A). Similarly, HCT-116 cells exhibited 19.02% early and 3.63% late apoptotic populations, compared to 0.46% and 0.15%, respectively, in untreated controls (Figure 3D vs. Figure 3C). Meanwhile, the necrotic population (Annexin-V−/PI+) accounted for 4.14% and 1.53% of cell death in LoVo and HCT-116 cells, respectively (Figure 3B and 3D). Additionally, total apoptosis reached 29.30% and 25.11% in LoVo and HCT-116 cells, respectively, compared to necrotic percentages of 1.38 and 1.63, (Figure 3E), confirming apoptosis as the predominant form of cell death.

**Figure 4:**
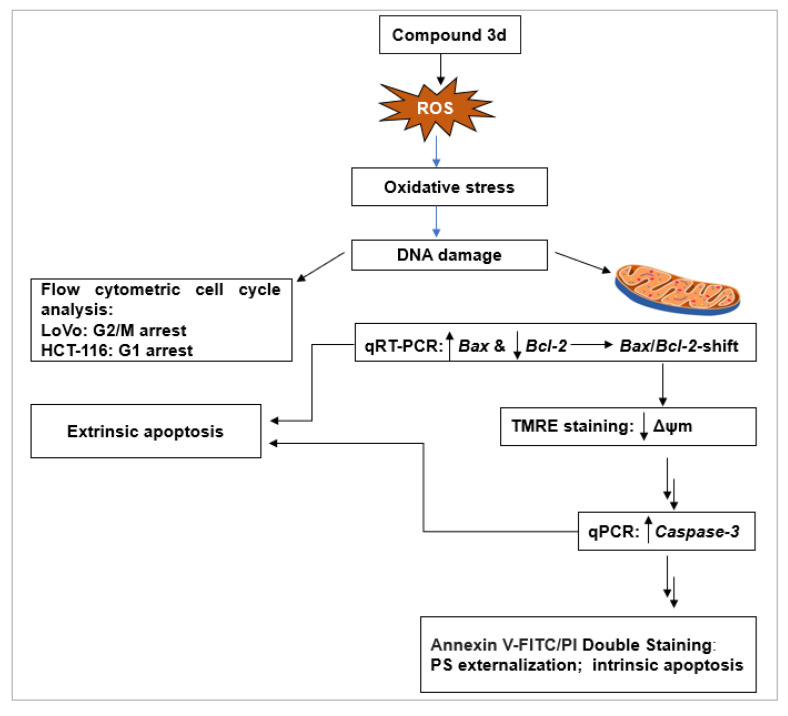
Mechanistic flowchart summarizing the sequential events through which compound **3d** exerts anticancer effects in colorectal carcinoma cells. Arrows indicate; pathway flow and potential crosstalk with extrinsic apoptosis. Abbreviations: ROS, reactive oxygen species; ΔΨm, mitochondrial membrane potential.

Collectively, the anticancer effects of compound **3d** are mediated through dual complementary mechanisms (Figure 4). First, it suppresses cell proliferation by inducing G2/M arrest in LoVo cells and G1 arrest in HCT-116 cells. Second, it promotes cell death primarily through apoptosis, with minimal necrosis observed. Apoptosis was characterized by hallmark features consistent with the intrinsic (mitochondrial) pathway, including increased ROS generation, mitochondrial membrane depolarization, upregulation of *caspase-3*, and a pronounced pro-apoptotic shift in the *Bax/Bcl-2* ratio—calculated as 12.63 in LoVo and 8.71 in HCT-116 cells. The concurrent upregulation of *Bax* and *caspase-3* may also suggest potential crosstalk with the extrinsic pathway [54]. Overall, the significant increase in Annexin V-positive cells, in conjunction with other apoptotic markers, confirms that apoptosis as the predominant mode of cell death induced by compound **3d** in LoVo and HCT-116 cells.

### 2.3. In Silico Studies

#### 2.3.1. Molecular Docking Studies

**Figure 5:**
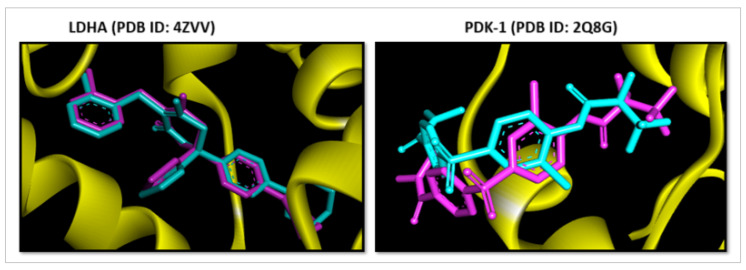
Validation of the docking process against LDHA and PDK-1 enzymes. Superimposition of the co-crystallized ligands (purple) and re-docked ligands (cyan).

The in vitro inhibitory efficiencies of compounds **3b** and **3d** against LDHA, and **3b** against PDK-1, as revealed by enzymatic assays were further substantiated through molecular docking simulations, which enabled the estimation of binding affinities and prediction of the binding modes within the active sites of the target enzymes. To validate the docking protocol, redocking experiments were performed using the co-crystallized ligands: GN0 for LDHA (PDB ID: 4ZVV) [55] and AZX for PDK-1 (PDB ID: 2Q8G) [56]. The computed root mean square deviation (RMSD) values were 0.79 Å for LDHA and 1.65 Å for PDK-1, both falling within the acceptable threshold (<2.0 Å) for reliable docking performance [57], thus confirming the suitability of the protocol (Figure 5). 

**Figure 6:**
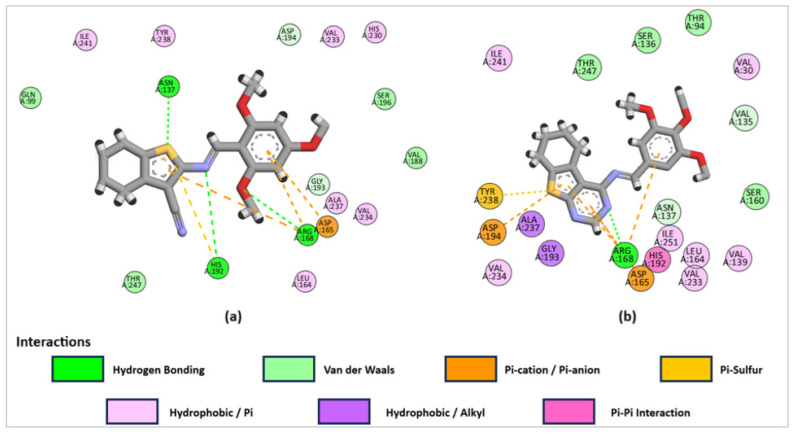
A 2D representation of the interactions with amino acid residues in the active binding site of LDHA: (**a**) Compound **3b**, (**b**) compound **3d**.

Docking results against LDHA revealed that both **3b** and **3d** (Figure 6) exhibited favorable binding energies of −8.1 and −7.9 kcal/mol, respectively, comparable to that of the native ligand GN0 (−8.8 kcal/mol). These compounds formed key binding interactions with residues Asn137 and Arg168. In compound 3b, one of the methoxy oxygen atoms formed a hydrogen bond with Arg168, while the imino nitrogen and thienyl sulfur bound to His192 and Asn137, respectively. For compound **3d**, the imino nitrogen at position-3 of the thienopyrimidine ring interacted via hydrogen bonding with Arg168. Additional intermolecular bonding through pi-anion and hydrophobic interactions were observed involving Asn137, His192, and Asp165 amino acid residues. These interactions suggest that both compounds occupy a similar binding pocket to GN0, engaging residues crucial for LDHA inhibition and complex stability. These results align with findings by Sharma, who identified the pharmacophoric features (aromatic rings and H-bond acceptors) and the residues essential for LDHA inhibition [58].

**Figure 7:**
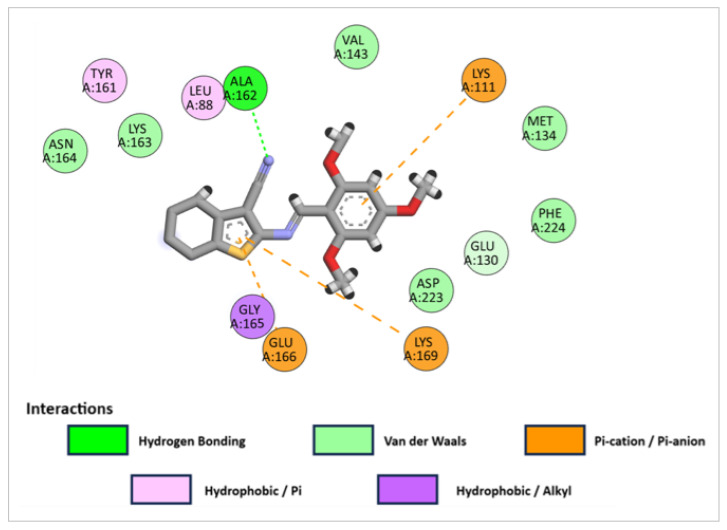
A 2D representation of the interactions between compound **3b** and amino acid residues in the active binding site of PDK-1.

In the case of PDK-1, compound **3b** also exhibited a favorable docking score (−7.7 kcal/mol), though slightly higher than that of the reference ligand AZX (−8.4 kcal/mol). It established a hydrogen bond between the nitrogen atom of its nitrile group and Ala162 residue; meanwhile its aryl rings engaged in pi-cation interactions with the amino acids Lys111, Glu166, and Lys169. These main interactions are supported by additional hydrophobic contacts (Figure 7).

#### 2.3.2. In Silico Predictions of the Physicochemical, Drug-Likeness, and ADMET Properties of Compounds **3b** and **3d**

The physicochemical properties, drug-likeness, absorption, distribution, metabolism, excretion, and toxicity (ADMET) characteristics of compounds **3b** and **3d** were predicted using the free webserver ADMETlab 2.0 (https://admetmesh.scbdd.com/, accessed on 7 February 2024) [59].

##### Analyses of Physicochemical Characteristics

**Figure 8:**
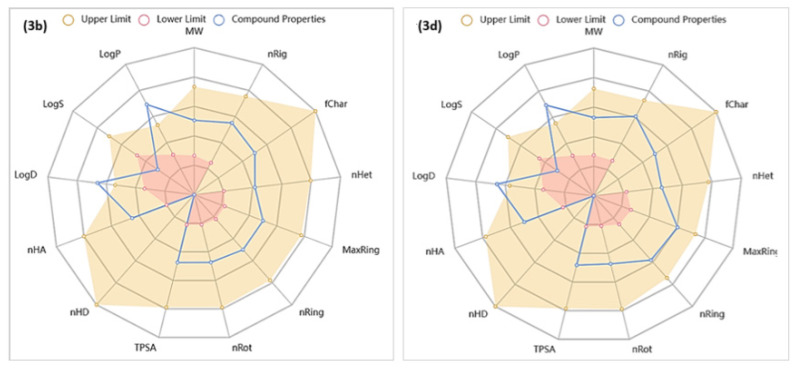
Radar chart representations of compounds **3b** and **3d**, exhibiting their physicochemical properties.

The physicochemical properties influencing the drug’s capability to penetrate membranes for transport throughout the body were generated. As shown in the radar charts (Figure 8), both compounds satisfied the number of hydrogen bond acceptor groups (nHA), number of hydrogen bond donating groups (nHD), topological polar surface area (TPSA), number of rotatable bonds (nRot), number of rings (nRing), number of atoms in the biggest ring (MaxRing), number of heteroatoms (nHet), formal charge (fChar), number of rigid bonds (nRig), and molecular weight (MW); however, they violated lipophilicity and solubility parameters.

The calculated LogP values were 4.03 (**3b**) and 3.21 (**3d**), whereas the acceptable range is 0 to 3 mol/L. This suggests that both compounds possess moderate to high lipophilicity, with **3b** being more lipophilic. It is reported that high lipophilicity increases the risk of poor aqueous solubility, poor absorption, in vivo toxicity, reduced in vitro receptor selectivity [60], and metabolic instability. The computed values for solubility descriptors LogS and LogD were −6.05/−4.24 (optimal range −4 to 0.5 mol/L) and 3.68/3.021 (proper range 1 to 3 log mol/L) for **3b**/**3d**, respectively. Based on these values, **3b** may be considered a poorly soluble compound, which may result in poor absorption from the gastrointestinal tract. Also, **3d** is still a poorly soluble candidate, but it is comparatively better than **3b**, suggesting that it may have more favorable absorption characteristics. Overall, the high lipophilicity of compounds **3b** and **3d** may favor membrane permeation, but it can also lead to poor solubility, which may negatively impact their oral bioavailability and overall pharmacokinetic profiles. Therefore, optimizing solubility without compromising permeability is essential for minimizing potential drawbacks and enhancing their drug-likeness.

##### Analyses of Drug-Likeness Characteristics for Compounds **3b** and **3d**

The likelihood of the studied derivatives being developed into drugs was assessed using a number of medicinal chemistry filters. According to quantitative estimate of drug-likeness (QED) scores [61], **3b** was predicted to be an attractive candidate, while **3d** was ranked as an unattractive molecule. Calculated synthetic accessibility scores (SA score) implied the ease of synthesis of both compounds [62]. With respect to the medicinal chemistry evolution in 2018 (MCE-18) filter, which is a measure for scoring molecules by novelty according to their sp^3^ complexity [63], compound **3d** showed a suitable value, whereas **3b** did not. The four physicochemical parameters of Lipinski’s rule of five [64]—including LogP (less than 5), MW (less than 500), nHA (not more than 10), and nHD (not more than 5)—which determine the suitability of a drug candidate to be orally administrated, were satisfied by both compounds except for the LogP values. Therefore, both compounds are expected to be absorbed through the intestinal walls. Finally, the pan assay interference compounds (PAINS) filter [65], which flags suspicious compounds by identifying substructural alerts that may give false positive results in biochemical assays, showed that both derivatives had no PAINS chemotypes.

##### Analyses of ADMET Characteristics for Compounds **3b** and **3d**

- Absorption

The Caco-2 (the human colon adenocarcinoma cell line) permeability [66] parameter is used to predict the human intestinal absorption (HIA) of a compound, which is an important feature for a drug to be properly absorbed through the intestinal membrane. The estimated values for **3b** and **3d** were optimal. The human oral bioavailability 30% (F_30%_) values were also predicted to be good for both compounds. Screening both compounds for permeability glycoprotein (P-gp) [67]—an efflux protein that recognizes xenobiotics and is considered a cause of drug resistance—indicated that both compounds had high probabilities of being P-gp substrates; thus, significant drug–drug interactions (DDIs) would be expected if co-administrated with other P-gp inhibitors.

- Distribution

Prediction of plasma protein binding (PPB) indicated that both derivatives had a high ability to bind to plasma proteins. Although this finding suggests a narrow therapeutic index, it can also be advantageous, as it indicates the prolonged duration of the drugs’ action [68]. The computed volume of distribution (VD) values were optimal for both compounds, indicating favorable distribution throughout the body’s tissues rather than plasma confinement. Additionally, the studied compounds are expected to penetrate the blood–brain barrier (BBB) due to their high lipophilicity; thus, they could potentially cause central nervous system (CNS) side effects.

- Metabolism

Drug metabolism includes two phases: phase I (functionalization reactions; oxidation, reduction, and hydrolysis) and phase II (conjugation reactions). The human cytochrome P450 enzyme (CYP) family is responsible for the phase I metabolism of about two thirds of the know drugs [69], particularly five isoenzymes: CYP1A2, CYP3A4, CYP2C9, CYP2C19 and CYP2D6. Prediction of the affinities of **3b** and **3d** for these five CYP isoenzymes showed that they would be substrates and might act as inhibitors; thus, both compounds are expected to cause DDIs.

- Excretion

Total clearance (CL) parameters predict the removal rate of a drug from systemic circulation by all means in mL/min/kg. CL is an important pharmacokinetic parameter, as it is a determinant of half-life time (t_1/2_), oral bioavailability and the efficacious dose of a drug [70]. Both compounds are predicted to have excellent clearance and t_1/2_ values exceeding 3 hours.

- Toxicity

Some toxicity properties were assessed, such as blocking of the human ether-a-go-go-related gene (hERG) [71], which plays a major role in regulating cardiac action potential. According to predictions, both compounds may block this gene, and therefore they may cause prolongation of QT interval and palpitations. Hepatotoxicity probability was predicted to be intermediate for both derivatives. The predicted FDA maximum daily dose (FDAMDD) value [72], which estimates the toxic dose threshold of chemicals in humans, indicates a high and a medium probability of compounds **3b** and **3d** being toxic, respectively.

## 3. Materials and Methods

### 3.1. Chemistry

#### 3.1.1. Instrumentation

Melting points (°C) were recorded on a Gallen Kamp melting point apparatus. The IR spectra were recorded on a Perkin Elmer FT spectrophotometer, Spectrum BX 1000 (PerkinElmer, Waltham, MA, USA) in wave number ν (cm^−1^) using KBr discs. Nuclear magnetic resonance (NMR) analyses were performed using an Eclipse 300 FT NMR spectrometer (Tokyo, Japan) operating at 300 MHz for ^1^H-NMR and at 75 MHz for ^13^C-NMR, using DMSO-*d*_6_ or CDCl_3_ as solvents. Chemical shifts were expressed in (ppm), and coupling constants (*J*) were expressed in Hz. Splitting patterns were designated as s (singlet), br. s (broad singlet), d (doublet), dd (doublet of doublets), t (triplet), q (quartet) and m (multiplet). Mass spectra were carried out on a direct probe controller inlet part of a single quadrupole mass analyzer (Thermo Scientific GCMS), model ISO Lt with Thermo X-Calibur Software (Thermo Fisher Scientific, Waltham, MA, USA).

##### General Procedure A for Synthesis of Various Schiff Bases

An equimolar mixture (0.01 mol) of the appropriate heterocyclic amine**—**namely, ethyl 2-amino-6-phenyl-4,5,6,7-tetrahydrobenzo[*b*]thiophene-3-carboxylate **1a** [25]; 2-amino-4,5,6,7-tetrahydrobenzo[*b*]thiophene-3-carbonitrile **1b** [26]; 5,6,7,8-tetrahydrobenzo[4,5]thieno[2,3-*d*]pyrimidin-4-amine **1c** [27]; ethyl 2-amino-4-methylthiazole-5-carboxylate **4** [28]; 3-amino-6,7-dimethoxy-2-methylquinazolin-4(3*H*)-one **15a** [30]; or 3-amino-6-fluoro-2-methylquinazolin-4(3*H*)-one **15b** [31]**—**and the corresponding aldehyde **2a**–**h**: 3-bromo-4-hydroxy-5-methoxy benzaldehyde; 2,4,6-trimethoxybenzaldehyde; 3,4,5-trimethoxybenzaldehyde; 2-hydroxy-1-naphthaldehyde, 4-(trifluoro-methyl)benzaldehyde; 4-hydroxy-3-methoxybenzaldehyde; isovanillin; or thiophene-2-carbaldehyde, respectively, in absolute ethanol (30 mL) containing a catalytic amount of glacial acetic acid (3 mL) was heated under reflux for 12 h. Afterwards, the excess solvent was evaporated, and the resulting solid was recrystallized from ethanol to afford the pure products.

#### 3.1.2. Synthesis of Thiophene Schiff Bases **3a**–**e**

They were prepared according to general procedure A using amines **1a**–**c** and aldehydes **2a**–**d**.

(*E*)-Ethyl2-((3-bromo-4-hydroxy-5-methoxybenzylidene)amino)-6-phenyl-4,5,6,7-tetrahydrobenzo[*b*]thiophene-3-carboxylate **3a**

Yellow powder; yield (65%); m.p.107–109 °C; IR (KBr): ν_max_ (cm^−1^) 3315 (OH), 2975 (CH-Ar), 2929 and 2847 (CH-aliphatic), 1676 (C=O), 1586 (C=N), 1500, 1436 and 1426 (C=C), 1355, 1289, 1156, 1044, 971, 855, 831, 791, 755, 678, 537; ^1^H-NMR (300 MHz, DMSO-*d*_6_): *δ*_H_ 1.24 (t, 3H, *J =* 7.1 Hz, CH_3_), 1.80–1.95 (m, 2H, CH_2_), 2.55–2.65 (m, 3H, CH_2_ and CH), 2.80–2.87 (m, 2H, CH_2_), 3.90 (s, 3H, OCH_3_), 4.14 (q, 2H, *J =* 7.1 Hz, CH_2_O), 7.19–7.29 (m, 6H, 6 × CH-Ar), 7.41 (s, 2H, CH-Ar), 7.71 (s, 1H, CH=N), 9.77 (s, 1H, OH); ^13^C-NMR (75 MHz, DMSO-*d*_6_): *δ*_C_ 14.60 (CH_3_), 27.09, 29.96, 31.95 (3 × CH_2_ and CH), 56.52 (OCH_3_), 58.91 (OCH_2_), 102.74, 109.44, 109.77, 115.29, 126.32, 127.01, 128.53, 128.87, 129.13, 131.34, 146.17 (7 × CH-Ar and 7 × C_q_-Ar), 148.85, 150.03 (2 × C_q_-O), 163.40 (CH=N), 165.30 (C=O); MS (EI, 70 eV): *m*/*z* (%) [M^+^ + 4] 517.61 (17.30) for C_25_H_24_BrNO_4_S, 498.40 (11.48), 485.56 (15.43), 454.41 (11.30), 410.36 (29.63), 382.13 (15.12), 321.27 (14.23), 292.62 (11.09), 270.15 (13.27), 235.09 (32.14), 223.32 (62.59), 196.15 (17.23), 131.13 (30.73), 102.23 (32.52), 76.18 (48.12), 64.19 (100.00), 54.70 (37.78), 44.24 (17.52).

(*E*)-2-((2,4,6-Trimethoxybenzylidene)amino)-4,5,6,7-tetrahydrobenzo[*b*]thiophene-3-carbonitrile **3b**

Yellowish green powder; yield (52%); m.p. 173–175 °C; IR (KBr): ν_max_ (cm^−1^) 3055 (CH-Ar), 2930 and 2830 (CH-aliphatic), 2214 (CN), 1599 (C=N), 1563, 1502 and14656 (C=C), 1413, 1331, 1230, 1209, 1157, 1118, 1033, 954, 888, 811, 640, 511; ^1^H-NMR (300 MHz, DMSO-*d*_6_): *δ*_H_ 1.78 (apparent s, 4H, 2 × CH_2_), 2.64 (apparent s, 4H, 2 × CH_2_), 3.87 (s, 9H, 3 × OCH_3_), 6.33 (s, 2H, 2 × Ar-H), 8.70 (s, 1H, CH=N); ^13^C-NMR (75 MHz, DMSO-*d*_6_): *δ*_C_ 21.95, 22.96, 24.18, 24.91 (4 × CH_2_), 56.12 and 56.60 (3 × OCH_3_), 91.63 (2 × CH-_trimethoxy phenyl_), 103.28, 105.31 (C_q_ and C_q_-CN), 114.96, 117.13 (CN and C_q-cyclohexene_), 134.30 (C_q_-S), 154.67 (N-C_q_-S), 162.99, 163.57, 165.56 (CH=N and 3 × C_q_-OCH_3_); MS (EI; *m*/*z*; %): [M^+^ + 2] 358.74 (58.73) for C_19_H_20_N_2_O_3_S, 342.57 (40.45), 322.03 (52.87), 297.42 (39.05), 278.53 (43.18), 245.48 (22.12), 231.42 (25.53), 217.35 (15.72), 201.59 (17.75), 188.78 (51.62), 172.39 (75.41), 146.71 (48.47), 93.66 (100.00), 76.39 (90.52), 65.90 (58.85), 41.03 (52.33).

(*E*)-2-Bromo-6-methoxy-4-(((5,6,7,8-tetrahydrobenzo[4,5]thieno[2,3-*d*]pyrimidin-4-yl) imino)methyl)phenol **3c**

Brown powder; yield (60%); m.p. 130–133 °C; IR (KBr): ν_max_ (cm^−1^) 3317 (OH), 3024 (CH-Ar), 2934 and 2854 (CH-aliphatic), 1675 (N-C_q_=N), 1587 (2 × HC=N), 1500, 1461, (C=C), 1427, 1358, 1289, 1158, 1044, 988, 911, 854, 830, 792, 791, 677 (C-Br), 578, 488; ^1^H-NMR (300 MHz, DMSO-*d*_6_): *δ*_H_ 1.72–1.74 (m, 4H, 2 × CH_2_), 2.65–2.85 (m, 4H, 2 × CH_2_), 3.88 (s, 3H, OCH_3_), 6.74 (br. s, 1H, OH), 7.36 (s, 1H, CH-Ar), 7.64 (s, 1H, CH-Ar), 7.95 (s, 1H, CH=N-_azomethine_), 9.72 (s, 1H, CH=N-_pyrimidine_); ^13^C-NMR (75 MHz, DMSO-*d*_6_) *δ*_C_: 21.78, 22.48, 25.33, 25.46 (4 × CH_2_), 56.33 (OCH_3_), 115.09, 126.85, 128.57, 128.91, 130.79, 131.10, 132.08, (2 × CH-Ar, C_q_-Br and 4 × C_q_-Ar), 144.69, 148.62, 149.84, 157.67, 158.04, 162.41 (2 × C_q_-O_3_, CH=N-_azomethine_, CH=N-_pyrimidine_, N-C_q_=N and S-C**_q_**=N); MS (EI, 70 eV): *m*/*z* (%) [M^+^ + 4] 421.03 (12.56) for C_18_H_16_BrN_3_O_2_S, 338.21 (25.20), 318.08 (35.19), 298.60 (89.08), 283.16 (23.51), 225.28 (35.19), 197.33 (100.00), 180.88 (48.71), 153.21 (71.21), 138.29 (64.60), 106.57 (65.13), 64.51 (77.28), 45.36 (41.56).

(*E*)-*N*-(5,6,7,8-Tetrahydrobenzo[4,5]thieno[2,3-*d*]pyrimidin-4-yl)-1-(3,4,5-trimethoxyphenyl)methanimine **3d**

Yellow powder; yield (82%); m.p. 153–155 °C; IR (KBr): ν_max_ (cm^−1^) 3013 (CH-Ar), 2939, 2844 and 2754 (CH-aliphatic), 1683 (HC=N), 1587, 1505, 1462 (C=C), 1426, 1392, 1329, 1236, 1127, 728, 989, 845, 785, 756, 728, 626, 591, 521, 461; ^1^H-NMR (300 MHz, DMSO-*d*_6_): *δ*_H_ 1.72–1.74 (m, 4H, 2 × CH_2_), 2.65–2.85 (m, 4H, 2 × CH_2_), 3.75 (s, 3H, OCH_3_), 3.84 (s, 6H, 2 × OCH_3_), 7.23 (s, 2H, 2 × CH_-trimethoxy phenyl_), 8.16 (s, 1H, CH=N), 9.85 (s, 1H, CH=N-_pyrimidine_); ^13^C-NMR (75 MHz, DMSO-*d*_6_): *δ*_C_ 21.49, 22.07, 22.33, 24.89 (4 × CH_2_), 56.12 and 60.28 (3 × OCH_3_), 106.80, 115.16, 126.97, 131.12, 131.76, 142.97 (2 × CH-Ar and 6 × C_q_-Ar), 152.95, 153.44, 158.20, 165.56, 171.70 (3 × C_q_-OCH_3_, HC=N-_azomethine_, HC=N-_pyrimidine_ and C**_q_**=N); MS (EI, 70 eV): *m*/*z* (%) [M^+^ + 2] 385.34 (21.36) for C_20_H_21_N_3_O_3_S 369.39 (16.45), 359.93 (51.25), 334.14 (22.35), 319.90 (25.82), 292.99 (16.64), 270.28 (14.19), 242.16 (24.80), 216.23 (23.12), 200.21 (38.44), 183.18 (24.69), 156.44 (63.94), 130.69 (68.76), 115.02 (46.24), 98.15 (61.09), 62.99 (57.78), 51.16 (100.00).

(*E*)-1-(((5,6,7,8-Tetrahydrobenzo[4,5]thieno[2,3-*d*]pyrimidin-4-yl)imino)methyl) naphthalene-2-ol **3e**

Yellowish green powder; yield (78%); m.p. 237–240 °C; IR (KBr): ν_max_ (cm^−1^) 3331 (br., OH), 3043 (CH-Ar), 2927 (CH-aliphatic), 2369, 2342, 1655 (N-C_q_=N), 1590 (2 × HC=N), 1512, 1463 and 1436 (C=C), 1374, 1311, 1247, 1176 (C_q_-O), 1076, 990, 965, 861, 785, 745, 713, 652, 559; ^1^H-NMR (300 MHz, DMSO-*d*_6_): *δ*_H_ 1.56–1.76 (m, 4H, 2 × CH_2_), 2.59–2.87 (m, 4H, 2 × CH_2_), 5.35 (br. s, 1H, OH), 7.18 (d, 1H, *J =* 8.7 Hz, CH-Ar), 7.34 (t, 1H, *J =* 7.2 Hz, CH-Ar), 7.55 (t, 1H, *J =* 7.2 Hz, CH-Ar), 7.79 (d, 1H, *J =* 7.8 Hz, CH-Ar), 7.98 (s, 1H, CH=N-_azomethine_), 8.04 (d, 1H, *J =* 9.0 Hz, CH-Ar), 8.19 (s, 1H, CH=N-_pyrimidine_), 8.94 (d, 1H, *J =* 8.1 Hz, CH-Ar); ^13^C-NMR (75 MHz, DMSO-*d*_6_): *δ*_C_ 21.65, 21.89, 24.49, 25.00 (4 × CH_2_), 112.19, 114.72, 119.61, 121.75, 122.37, 123.35, 126.79, 128.70, 130.64, 131.77, 137.82, 144.46 (6 × CH-Ar and 7 × C**_q_**-Ar), 152.54, 157.76, 162.09, 165.60 (C_q_-OH, N-C**_q_**=N, CH=N_-azomethine_ and HC=N_-pyrimidine_); MS (EI, 70 eV): *m*/*z* (%) [M^+^ + 2] 361.40 (0.80), [M^+^ + 1] 360.09 (8.56), [M^+^] 359.20 (28.10) for C_21_H_17_N_3_OS, 345.60 (21.16), 327.49 (16.06), 318.40 (89.72), 295.51 (26.68), 259.58 (16.79), 245.01 (12.03), 216.59 (64.13), 196.96 (11.58), 172.34 (15.17), 147.56 (22.36), 124.51 (85.69), 109.97 (100.00), 104.71 (55.63), 76.72 (60.02), 62.73 (27.47), 48.30 (7.51).

#### 3.1.3. Synthesis of Ethyl 2-(3,5-Dioxothiomorpholino)-4-methylthiazole-5-carboxylate **8**

To a stirred, ice-cooled solution of ethyl 2-amino-4-methylthiazole-5-carboxylate **4** (3.6 g, 0.02 mol) in dry CHCl_3_ (30 mL), triethylamine (2.8 mL, 0.02 mol) was added, followed by the dropwise addition of chloroacetyl chloride **5** (1.6 mL, 0.02 mol). The reaction mixture was stirred further at 0 °C for one hour, then allowed to warm to room temperature, and subsequently refluxed for 16 h. After evaporation of the solvent under reduced pressure, the resulting solid was washed with a saturated solution of NaHCO_3_, followed by water, and air-dried. Recrystallization from ethanol afforded the pure chloroacetamide intermediate **6**.

An equimolar mixture of chloroacetamide intermediate **6** and thioglycolic acid **7** (0.004 mol) in dry pyridine (15 mL) was refluxed for 16 h. After cooling to room temperature, the precipitated solid was filtered, dissolved in diethyl ether, and triturated with aqueous ethanol until precipitation occurred. The solid was collected by filtration and recrystallized from ethanol to afford compound **8** as a buff-colored powder; yield (55%); m.p. 180–183 °C; IR (KBr): ν_max_ (cm^−1^) 2926 (CH-aliphatic), 1705 (br. 3 × CO), 1547 (C=N), 1464 (C=C), 1372, 1318, 1266, 1161, 1095, 948, 760, 683, 592, 449; ^1^H-NMR (300 MHz, DMSO-*d*_6_): *δ*_H_ 1.27 (t, 3H, *J =* 7.1 Hz, CH_3_-CH_2_), 2.57 (s, 3H, CH_3_), 3.58 (s, 4H, 2 × CH_2_S), 4.24 (q, 2H, *J =* 7.1 Hz, CH_2_O); ^13^C-NMR (75 MHz, DMSO-*d*_6_): *δ*_C_ 14.20, 16.99 (2 × CH_3_), 33.79, 34.34 (2 × CH_2_S), 60.55 (CH_2_O), 114.25, 156.18, 159.40, 162.08, 168.57, 170.80 (3 × C_q_ and 3 × C=O); MS (EI, 70 eV): *m*/*z* (%) [M^+^ + 4] 304.55 (38.60) for C_11_H_12_N_2_O_4_S_2_, 232.06 (63.58), 222.22 (100.00), 206.85 (60.18), 132.17 (55.79).

#### 3.1.4. Synthesis of Thiazolyl Schiff Bases **9a**–**c**

These were prepared according to general procedure A using thiazolyl amine **4** and aldehydes **2a**, **2c** and **2d**, respectively.

Ethyl (*E*)-2-((3-bromo-4-hydroxy-5-methoxybenzylidene)amino)-4-methyl thiazole-5-carboxylate **9a**

Yellow Powder; yield (75%); m.p. 143–145 °C; IR (KBr): ν_max_ (cm^−1^) 3376 (OH), 3098 (CH-Ar), 2979 and 2933 (CH-aliphatic), 1680 (C=O), 1587 (CH=N and S-C_q_=N), 1504, 1462, 1428 (C=C), 1373, 1279 (C-O), 1157, 1100, 1043, 972, 853, 828, 791, 759, 677 (C-Br), 588, 493; ^1^H-NMR (300 MHz, DMSO-*d*_6_): *δ*_H_ 1.18 (t, 3H, *J =* 7.1 Hz, CH_3_-CH_2_-O), 2.37 (s, 3H, CH_3_), 3.86 (s, 3H, OCH_3_), 4.14 (q, 2H, *J =* 7.1 Hz, CH_3_-CH_2_-O), 7.38 (d, 1H, *J =* 1.8 Hz, CH-Ar), 7.65 (apparent d, 1H, *J =* 1.5 Hz, CH-Ar), 7.72 (s, 1H, CH=N), 9.74 (s, 1H, OH); ^13^C-NMR (75 MHz, DMSO-*d*_6_): *δ*_C_ 14.33, 17.01 (2 × CH_3_), 56.36 (OCH_3_), 59.86.(OCH_2_), 107.63, 109.29, 109.67, 128.54, 128.94 (2 × CH-Ar, C_q_-S, C_q_-Ar and C_q_-Br), 148.62, 149.80, 158.73, 158.86, 161.93, 170.21 (N-C_q_-CH_3_, 2 × C_q_-O, CH=N, N=C_q_-S and C=O); MS (EI, 70 eV): *m*/*z* (%) [M^+^ + 4] 401.94 (8.33) for C_15_H_15_BrN_2_O_4_S, 384.87 (12.31), 355.44 (25.54), 290.12 (26.20), 243.66 (54.01), 193.16 (28.22), 159.36 (40.18), 144.21 (84.24), 129.80 (69.64), 113.32 (100.00), 91.38 (86.76), 84.04 (94.60), 51.27 (71.52), 43.14 (45.23).

Ethyl(*E*)-4-methyl-2-((3,4,5-trimethoxybenzylidene)amino)thiazole-5-carboxylate **9b**

Yellow Powder; yield (60%); m.p. 110–113 °C; IR (KBr): ν_max_ (cm^−1^) 3086 (CH-Ar), 2977, 2939 (CH-aliphatic), 1680 (C=O), 1586 (CH=N and C_q_=N), 1514, 1466, 1426 (C=C), 1328, 1279 (C-O), 1125, 1094, 990, 844, 755, 628, 524, 423; ^1^H-NMR (300 MHz, DMSO-*d*_6_): *δ*_H_ 1.22 (t, 3H, *J =* 7.1 Hz, CH_3_-CH_2_-O), 2.37 (s, 3H, CH_3_), 3.77 (s, 3H, OCH_3_), 3.86 (s, 6H, 2 × OCH_3_), 4.15 (q, 2H, *J =* 7.1 Hz, CH_3_-CH_2_-O), 7.23 (s, 2H, 2 × CH-Ar), 7.70 (s, 1H, CH=N); ^13^C-NMR (75 MHz, DMSO-*d*_6_): *δ*_C_ 14.24, 17.21 (2 × CH_3_), 55.97 (2 × OCH_3_), 59.92, 60.12 (OCH_3_ and OCH_2_), 106.67 (2 × CH-Ar), 107.42 (C_q_-S), 131.62, 142.84, 153.29, 159.24, 161.91, 170.21 (C_q_-Ar, N-C_q_-CH_3_, 3 × C_q_-OCH_3_, CH=N, S-C_q_=N and C=O); MS (EI, 70 eV): *m*/*z* (%) [M^+^ + 2] 366.33 (4.87) for C_17_H_20_N_2_O_5_S, 336.86 (19.60), 361.41 (17.33), 292. (8.97), 270.38 (15.79), 255.85 (16.42), 219.69 (7.39), 193.86 (11.61), 177.30 (16.45), 139.85 (24.95), 125.16 (19.41), 92.02 (24.62), 77.62 (49.20), 64.14 (100), 50.33 (34.70).

Ethyl(*E*)-2-(((2-hydroxynaphthalen-1-yl)methylene)amino)-4-methylthiazole-5-carboxylate **9c**

Buff powder; yield (60%); m.p. 174–176 °C; IR (KBr): ν_max_ (cm^−1^) 3375 (OH), 3088 (CH-Ar), 2982, 2923 (CH-aliphatic), 1707 (C=O), 1676, 1624 (CH=N and S-C_q_=N), 1549, 1515, 1463 (C=C), 1372, 1318, 1265 (C-O), 1210, 1151, 1090, 813, 752, 713, 589, 530, 421; ^1^H-NMR (300 MHz, DMSO-*d*_6_): *δ*_H_ 1.23 (t, 3H, *J =* 7.1 Hz, CH_3_-CH_2_-O), 2.36 (s, 3H, CH_3_), 4.11 (q, 2H, *J =* 7.1 Hz, CH_3_-CH_2_-O), 7.15–7.26 (m, 1H, CH-Ar), 7.38–7.46 (m, 1H, CH-Ar), 7.60 (t, 1H, *J =* 7.2 Hz, CH-Ar), 7.70 (s, 2H, CH=N and OH), 7.86 (d, 1H, *J =* 7.8 Hz, CH-Ar), 8.10 (d, 1H, *J =* 9.0 Hz, CH-Ar), 8.92 (d, 1H, *J =* 8.7 Hz, CH-Ar); ^13^C-NMR (75 MHz, DMSO-*d*_6_): *δ*_C_ 14.27, 17.05 (2 × CH_3_), 59.71 (OCH_2_), 107.43, 112.35, 118.67, 121.99, 124.16, 127.53, 128.77, 129.19, 131.72, 138.35 (6 × CH-Ar, C_q_-S and 3 × C_q_-Ar), 159.14, 161.91, 163.88, 170.19 (CH=N, N-C_q_-CH_3_, N=C_q_-S, C=O and C_q_-OH); MS (EI, 70 eV): *m*/*z* (%) [M^+^ + 3] 343.61 (10.13), [M^+^ + 2] 342.35 (36.41), [M^+^ + 1] (35.12) for C_18_H_16_N_2_O_3_S, 321.81 (74.11), 301.97 (34.88), 271.94 (30.74), 252.88 (41.26), 238.88 (59.64), 226.69 (78.45), 194.41 (37.70), 167.62 (42.94), 147.92 (78.10), 139.90 (100.00), 119.61 (40.05), 87.81 (70.39), 60.36 (84.63), 42.49 (90.30).

#### 3.1.5. General Procedure B for the Synthesis of 3-Substituted-6,7-dimethoxy-2-methyl quinazolin-4(3*H*)-one Derivatives **13** and **14**

Equimolar quantities (0.001 mol) of 6,7-dimethoxy-2-methyl-4*H*-benzo[*d*][1,3]oxazin-4-one **10a** [30] and 4-(benzo[*d*]thiazol-2-yl)aniline **11** [29] or cyclohexylamine **12** were fused in an oil bath at 200 °C for 2 h. The reaction mixture was allowed to cool to 100 °C, then concentrated hydrochloric acid (3 mL, 37%) was added. After further cooling to room temperature, the mixture was neutralized with aqueous sodium hydroxide (25%). The resulting precipitate was collected by filtration, washed with water, air dried, and recrystallized from ethanol to afford compounds **13** and **14**, respectively.

3-(4-(Benzo[*d*]thiazol-2-yl)phenyl)-6,7-dimethoxy-2-methylquinazolin-4(3*H*)-one **13**

Green powder; yield (60%); m.p. 154–157 °C; IR (KBr): ν_max_ (cm^−1^) 3058, 3002 (CH-Ar), 2927, 2833 (CH-aliphatic), 1668 (C=O), 1605 (C=N), 1497 and 1465 (C=C), 1435, 1390, 1266, 1104, 1025, 969, 848, 758, 679, 523, 509; ^1^H-NMR (300 MHz, DMSO-*d*_6_): *δ*_H_ 2.23 (s, 3H, CH_3_), 3.94 (s, 3H, OCH_3_), 3.98 (s, 3H, OCH_3_), 6.82–6.94 (m, 1H, Ar-H), 7.08 (s, 1H, CH-_quinazoline_), 7.36–7.50 (m, 4H, 4 × Ar-H), 7.56 (s, 1H, CH-_quinazoline_), 7.88 (d, 1H, *J =* 8.1 Hz, Ar-H), 8.08 (d, 1H, *J =* 7.8 Hz, Ar-H), 8.26 (d, 2H, *J =* 8.7 Hz, 2 × Ar-H); ^13^C-NMR (75 MHz, DMSO-*d*_6_): *δ*_C_ 23.70 (CH_3_), 55.82 (2 × OCH_3_), 105.43, 106.89 (2 × CH-_quinazolinone_), 113.29, 121.27, 122.14, 123.00, 125.13, 125.72, 126.09, 128.51, 134.02, 134.73, 143.21, 148.53 (8 × CH-Ar and 6 × C_q_-Ar), 151.91, 153.57, 154.81, 161.13, 165.90 (2 × C_q_-OCH_3_, C=O and 2 × C_q_=N); MS (EI, 70 eV): *m*/*z* (%) [M^+^+ 2] 431.86 (19.55) for C_24_H_19_N_3_O_3_S, 337.23 (51.58), 269.57 (84.30), 91.49 (40.71), 78.25 (100.00), 51.63 (83.87).

3-Cyclohexyl-6,7-dimethoxy-2-methylquinazolin-4(3*H*)-one **14**

White powder; yield (70%); m.p. 100–103 °C; IR (KBr): ν_max_ (cm^−1^) 3025 (CH-Ar), 2926, 2852 (CH-aliphatic), 1659 (C=O), 1614 (C=N), 1572, 1502 (C=C), 1457, 1398, 1321, 1246, 1166, 1026, 978, 845, 781, 609, 453; ^1^H-NMR (300 MHz, CDCl_3_): *δ*_H_ 1.12–1.18 (m, 2H, CH_2_), 1.33–1.49 (m, 4H, 2 × CH_2_), 1.72–1.76 (m, 4H, 2 × CH_2_), 2.58 (s, 3H, CH_3_), 3.42 (app. s, 1H, CH-N), 3.84 (s, 3H, OCH_3_), 3.87 (s, 3H, OCH_3_), 6.97 (s, 1H, Ar-H), 7.35 (s, 1H, Ar-H); ^13^C-NMR (75 MHz, CDCl_3_): *δ*_C_ 23.78 (CH_3_), 25.09, 25.92, 28.21 (3 × CH_2_), 55.60, 55.89 (5 × OCH_3_), 59.83 (N-CH), 105.04, 106.96, 114.55, 142.89 (2 × CH-Ar and 2 × C_q_-Ar), 148.21, 153.38, 154.45, 161.04 (2 × C_q_-OCH_3_, C_q_=N and C=O); MS (EI, 70 eV): *m*/*z* (%) [M^+^] 301.91 (17.37) for C_17_H_22_N_2_O_3_, 239.47 (66.06), 107.94 (91.96), 100.29 (80.20), 75.25 (90.44), 69.08 (86.71), 53.83 (100.00).

#### 3.1.6. Synthesis of Ethyl (6,7-Dimethoxy-2-methyl-4-oxoquinazolin-3(4*H*)-yl)carbamate **17**

A mixture of 3-amino-6,7-dimethoxy-2-methylquinazolin-4(3*H*)-one **10a** (0.0028 mol) and ethyl chloroformate **16** (0.038 mol) was heated under reflux for 10 h. The excess reagent was removed under reduced pressure, and the resulting residue was triturated with diethyl ether to induce solidification. The precipitated solid was filtered, washed with water, air-dried, and recrystallized from benzene to afford the *title compound* as an off-white powder; yield (80%); m.p. 130–133 °C; IR (KBr): ν_max_ (cm^−1^) 3520 (NH), 3066 (CH-Ar), 2980, 2930 (CH-aliphatic), 1733, 1689 (2 × C=O), 1611 (C=N), 1505, 1464 (C=C), 1387, 1273, 1154, 1099, 864, 776, 688, 538; ^1^H-NMR (300 MHz, DMSO-*d*_6_) *δ*_H_: 1.21 (t, 3H, *J =* 7.1 Hz, CH_2_-CH_3_), 2.40 (s, 3H, CH_3_), 3.87 (s, 3H, OCH_3_), 3.90 (s, 3H, OCH_3_), 4.22 (q, 2H, *J =* 7.1 Hz, O-CH_2_-CH_3_), 7.05–7.16 (m, 1H, Ar-H), 7.40–7.42 (m, 1H, Ar-H), 10.39 (s, 1H, NH); ^13^C-NMR (75 MHz, DMSO-*d*_6_) *δ*_C_: 14.14 (CH_3_-CH_2_-O), 20.82 (CH_3_), 55.53, 55.91 (2 × OCH_3_), 61.56 (CH_2_O), 105.35, 107.95, 113.22, 142.55(2 × CH-Ar and 2 × C_q_-Ar), 148.60, 152.66, 154.76 (2 × C_q_-O and C_q_=N), 155.48, 158.55 (2 × C=O); MS (EI, 70 eV): *m*/*z* (%) [M^+^] 307.28 (5.96) for C_14_H_17_N_3_O_5_], 271.00 (35.41), 256.06 (35.00), 76.35 (42.35), 74.59 (100.00).

#### 3.1.7. Synthesis of Quinazolinone Based-Schiff Bases **18a**–**h**

These were prepared according general procedure A using amines **10a**,**b** and aldehydes **2b** and **2d**–**h**.

(*E*)-6,7-Dimethoxy-2-methyl-3-((2,4,6-trimethoxybenzylidene)amino)quinazolin-4(3*H*)-one **18a**

Yellow powder; yield (73%); m.p. 200–203 °C; IR (KBr): ν_max_ (cm^−1^) 3035 (CH-Ar), 2927 (CH-aliphatic), 2369, 1669 (C=O), 1610 (CH=N and C_q_=N), 1503 and 1458 (C=C), 1336, 1245, 1207 (C-O), 1131, 1027, 861, 802, 775, 637, 469; ^1^H-NMR (300 MHz, DMSO-*d*_6_): *δ*_H_ 2.44 (s, 3H, CH_3_), 3.63 (s, 9H, 3 × OCH_3_), 3.88 (s, 6H, 2 × OCH_3_), 6.22 (s, 2H, 2 × CH-_trimethoxy phenyl_), 7.09 (s, 1H, CH-_quinazolinone_), 7.40 (s, 1H, CH-_quinazolinone_), 8.84 (s, 1H, CH=N); ^13^C-NMR (75 MHz, DMSO-*d*_6_): *δ*_C_ 24.40 (CH_3_), 55.31, 55.68, 55.88 and 56.22 (5 × OCH_3_), 91.31(2 × CH-_quinazolinone_), 104.70, 107.45, 108.81, 113.61 (2 × CH-Ar, C_q-ipso_ and C_q_-C=O), 144.08, 147.92, 153.88, 155.53, 156.72, 159.01, 160.78 (C_q_=N, C_q_-N, -CH=N and 5 × O-C_q_), 161.73 (C=O); MS (EI, 70 eV): *m*/*z* (%) [M^+^] 413.27 (17.47) for C_21_H_23_N_3_O_6_, 406.96 (69.19), 398.89 (56.11), 386.69 (31.89), 377.44 (49.14), 337.29 (54.02), 350.60 (30.03), 337.29 (54.02), 325.76 (25.79), 297.19 (27.65) 272.82(17.65), 251.33 (59.99), 226.28 (100.00), 208.35 (31.73), 187.44 (97.92), 147.89 (49.86), 101.62 (48.17), 82.10 (38.26), 66.59 (30.50), 57.17 (28.28), 46.22 (24.82).

(*E*)-3-(((2-Hydroxynaphthalen-1-yl)methylene)amino)-6,7-dimethoxy-2-methylquin- azolin-4(3*H*)-one **18b**

Yellowish green powder; yield (75%); m.p. 205–207 °C; IR (KBr): ν_max_ (cm^−1^) 3308 (OH), 3020 (CH-Ar), 2927 (CH-aliphatic), 2371, 2341, 1667 (C=O), 1615 (CH=N and C_q_=N), 1600, 1502, 1464 (C=C), 1396, 1331, 1277, 1245, 1207 (C-O), 1021, 973, 864, 826, 798, 772, 739, 684, 605, 552, 428; ^1^H-NMR (300 MHz, DMSO-*d*_6_): *δ*_H_ 2.54 (s, 3H, CH_3_), 3.87 (s, 6H, 2 × OCH_3_), 7.02 (s, 1H, CH-_quinazolinone_), 7.09–7.48 (m, 4H, CH-_quinazolinone_, 2 × CH-Ar and CH=N), 7.59 (t, 1H, *J =* 7.4 Hz, CH-Ar), 7.89 (d, 1H, *J =* 7.2 Hz, CH-Ar), 8.04 (d, 1H, *J =* 9.0 Hz, CH-Ar), 8.80 (d, 1H, *J =* 8.1 Hz, CH-Ar), 9.67 (s, 1H, OH); ^13^C-NMR (75 MHz, DMSO-*d*_6_): *δ*_C_ 21.64 (CH_3_), 55.68 and 55.85 (2 × OCH_3_), 99.22, 104.74, 107.28, 112.76, 113.81, 119.18, 123.14, 128.89, 135.81, 139.16, 142.88, 148.30, 151.35, (8 × CH-Ar and 5 × C_q_-Ar), 154.58, 157.10, 159.43, 161.93, 169.00, 172.27 (3 × C_q_-O, CH=N, C_q_=N and C=O); MS (EI, 70 eV): *m*/*z* (%) [M^+^ + 3] 392.49 (3.60) for C_22_H_19_N_3_O_4_, 387.24 (70.29), 378.84 (8.91), 367.80 (21.98), 346.55 (23.79), 331.82 (22.02), 297.63 (12.21), 280.96 (11.59), 244.77 (8.58), 220.18 (63.36), 204.47 (13.31), 168.99 (31.47), 151.80 (20.15), 136.21 (81.96), 127.13 (100.00), 105.86 (22.75), 92.17 (47.14), 64.22 (87.35), 52.80 (73.61).

(*E*)-6,7-Dimethoxy-2-methyl-3-((4-(trifluoromethyl)benzylidene)amino)quinazolin-4(3*H*)-one **18c**

Yellow powder; yield (70%); m.p. 217–219 °C; IR (KBr): ν_max_ (cm^−1^) 3074 and 3012 (CH-Ar), 2936 (CH-aliphatic), 2371, 2341, 1670 (C=O), 1612 (CH=N and C_q_=N), 1509, 1471 and 1437 (C=C), 1394, 1325 (C-F), 1278, 1246, 1212, 1169, 1111, 1067, 1019, 985, 958, 870, 842, 806, 771, 680, 652, 601, 552, 515, 478; ^1^H-NMR (300 MHz, CDCl_3_): *δ*_H_ 2.65 (s, 3H, CH_3_), 3.98 (s, 6H, 2 × OCH_3_), 7.08 (s, 1H, CH-_quinazolinone_), 7.56 (s, 1H, CH-_quinazolinone_), 7.75 (d, 2H, *J =* 8.1 Hz, 2 × Ar-H), 7.97 (d, 2H, *J =* 7.8 Hz, 2 × Ar-H), 9.27 (s, 1H, CH=N); ^13^C-NMR (75 MHz, CDCl_3_): *δ*_C_ 22.57 (CH_3_), 56.12, 56.74 (2 × OCH_3_), 99.11, 105.91, 107.28, 114.43, 121.71, 125.72, 128.71, 133.48, 136.27, 142.48, 148.78 (6 × CH-Ar and 5 × C_q_-Ar), 152.69, 155.07, 158.22 (C_q_=N, CH=N and 2 × C_q_-O), 163.10 (C=O); MS (EI, 70 eV): *m*/*z* (%) [M^+^] 391.03 (4.37) for C_19_H_16_F_3_N_3_O_3_, 384.43 (10.21), 366.31 (5.93), 343.19 (100.00), 323.16 (13.07), 296.26 (12.21), 286.20 (9.93), 245.18 (14.02), 233.18 (10.08), 206.17 (20.75), 189.16 (38.35), 148.14 (93.61), 115.13 (17.48), 59.12 (13.13).

(*E*)-3-((4-Hydroxy-3-methoxybenzylidene)amino)-6,7-dimethoxy-2-methylquinazoli-*N*-4(3*H*)-one **18d**

Dark orange powder; yield (85%); m.p. > 300 °C; IR (KBr): ν_max_ (cm^−1^) 3451 (OH), 3142 and 3002 (CH-Ar), 2989 (CH-aliphatic), 2367, 1649 (C=O), 1574 (CH=N and C_q_=N), 1505, 1462 (C=C), 1399, 1334, 1300, 1251, 1210, 1128 (C-O), 1027, 975, 842, 810, 770, 675, 612, 548, 436; ^1^H-NMR (300 MHz, DMSO-*d*_6_): *δ*_H_ 2.45 (s, 3H, CH_3_), 3.90 (s, 9H, 3 × OCH_3_), 6.94–6.97 (m, 1H, CH-Ar), 7.07 (s, 1H, CH-_quinazolinone_), 7.30–7.44 (m, 2H, 2 × CH-Ar), 7.55 (s, 1H, CH-_quinazolinone_), 8.68 (s, 1H, CH=N), 10.04 (br. s, 1H, OH); ^13^C-NMR (75 MHz, DMSO-*d*_6_): *δ*_C_ 21.69 (CH_3_), 55.35, 55.61 (3 × OCH_3_), 105.10, 106.98, 107.15, 110.21, 113.57, 115.31, 123.42, 124.18 (5 × CH-Ar and 3 × C_q_-Ar), 142.21, 147.91, 151.09, 151.38, 154.18, 156.57 (4 × C_q_-O, C_q_=N and CH=N), 168.59 (C=O); MS (EI, 70 eV): *m*/*z* (%) [M^+^ + 3] 372.04 (1.85), [M^+^ + 2] 371.04 (10.91), [M^+^] 369.03 (32.42) for C_19_H_19_N_3_O_5_, 327.05 (30.05), 259.07 (4.35), 217.04 (100.00), 176.05 (6.34), 146.03 (1.78), 120.05 (3.28), 42.05 (17.41).

(*E*)-6-Fluoro-2-methyl-3-((2,4,6-trimethoxybenzylidene)amino)quinazolin-4(3*H*)-one **18e**

Off-white powder; yield (80%); m.p. 220–222 °C; IR (KBr): ν_max_ (cm^−1^) 3072 (CH-Ar), 2926 (CH-aliphatic), 1674 (C=O), 1610 (CH=N and C_q_=N), 1480, 1413 (C=C), 1333, 1227 (C-O), 1133 (C-F), 1037, 950, 876, 827, 799, 641, 486; ^1^H-NMR (300 MHz, CDCl_3_): *δ*_H_ 2.57 (s, 3H, CH_3_), 3.89 (s, 9H, 3 × OCH_3_), 6.13 (s, 2H, 2 × CH-_trimethoxy phenyl_), 7.40 (dd, 1H, *J* = 8.1, 3.1 Hz, CH-Ar), 7.60–7.65 (apparent m, 1H, CH-Ar), 7.86–7.91 (apparent m, 1H, CH-Ar), 8.93 (s, 1H, CH=N); ^13^C-NMR (75 MHz, CDCl_3_): *δ*_C_ 22.53 (CH_3_), 55.33 and 55.99 (3 × OCH_3_), 90.53 (2 × CH-_trimethoxy phenyl_), 102.82, 111.79, 122.36, 122.68, 128.95, 143.33 (3 × CH-Ar and 3 × C_q_-Ar), 153.15, 157.60, 158.52, 161.79, 162.17, 164.94 (3 × C_q_-O, C_q_=N, CH=N, C=O and C-F); MS (EI, 70 eV): *m*/*z* (%) [M^+^] 371.21 (20.10) for C_19_H_18_FN_3_O_4_, 356.84 (16.73), 342.27 (23,12), 326.27 (21.85), 297.94 (11.17), 281.57 (25.34), 252.84 (86.17), 235.23 (28.44), 215.02 (23.88), 195.68 (27.22), 151.81 (45.41), 124.28 (21.12), 104.74 (100.00), 77.07 (89.61), 48.16 (47.46).

(*E*)-6-Fluoro-3-(((2-hydroxynaphthalen-1-yl)methylene)amino)-2-methylquinazolin-4(3*H*)-one **18f**

Yellowish orange powder; yield (88%); m.p. 207–209 °C; IR (KBr): ν_max_ (cm^−1^) 3446 (OH), 3071 (CH-Ar), 2370, 2341, 2925 (CH-aliphatic), 2369, 2341, 1679 (C=O), 1599 (CH=N and C_q_=N), 1485 (C=C), 1319 (C-F), 1276, 1242, 1187 (C-O), 1096, 1035, 961, 892, 824, 771, 741, 672, 594, 560, 531, 428; ^1^H-NMR (300 MHz, DMSO-*d*_6_): *δ*_H_ 2.51 (s, 3H, CH_3_), 7.33 (d, 1H, *J =* 9.0 Hz, CH-Ar), 7.45 (t, 1H, *J =* 7.5 Hz, CH-Ar), 7.63 (t, 1H, *J =* 7.8 Hz, CH-Ar), 7.69–7.86 (m, 3H, 2 × CH-Ar and CH=N), 7.85 (apparent dd, 1H, *J =* 8.7, 2.4 Hz, CH-Ar), 7.93 (d, 1H, *J =* 7.8 Hz, Ar-H), 8.11 (d, 1H, *J =* 9.0 Hz, Ar-H), 8.82 (d, 1H, *J =* 8.4 Hz, Ar-H), 9.67 (s, 1H, OH); ^13^C-NMR (75 MHz, DMSO-*d*_6_): *δ*_C_ 22.26 (CH_3_), 108.30, 111.06, 111.37, 118.55, 123.31, 124.07, 128.03, 128.72, 129.04, 129.37, 132.05, 136.29, 143.02 (9 × CH-Ar and 5 × C_q_-Ar), 152.83, 156.98, 158.23, 160.66, 169.70 (CH=N, C_q_=N, C_q_-O, C_q_-F and C=O); MS (EI, 70 eV): *m*/*z* (%) [M^+^] 347.00 (16.59) for C_20_H_14_FN_3_O_2_, 320.34 (54.51), 298.98 (29.25), 275.72 (27.16), 267.46 (23.16), 244.82 (24.16), 222.86 (20.75), 190.87 (100.00), 186.10 (68.87), 177.91 (33.65), 165.55 (22.57), 150.57 (45.77), 125.90 (46.98), 102.68 (47.54), 84.16 (19.41), 51.82 (54.71).

(*E*)-6-Fluoro-3-((3-hydroxy-4-methoxybenzylidene)amino)-2-methylquinazolin-4(3*H*)-one **18g**

Yellowish white powder; yield (72%); m.p. 183–185 °C; IR (KBr): ν_max_ (cm^−1^) 3301 (OH), 30,188 and 3071 (CH-Ar), 2930 (CH-aliphatic), 2371, 1675 (C=O), 1637 (CH=N and C_q_=N), 1598, 1513, 1483 (C=C), 1442, 1380, 1350, 1279 (C-F), 1240, 1194, 1158 (C-O), 1109, 1027, 964, 993, 841, 7729, 706, 644, 601, 565, 474; ^1^H-NMR (300 MHz, DMSO-*d*_6_): *δ*_H_ 2.48 (s, 3H, CH_3_), 3.86 (s, 3H, OCH_3_), 7.10 (apparent dd, 1H, *J =* 8.4, 2.4 Hz, CH-Ar), 7.30 (apparent dd, 1H, *J =* 8.3, 2.0 Hz, CH-Ar), 7.47 (d, 1H, *J =* 8.3, 2.1 Hz, CH-Ar), 7.68–7.88 (m, 3H, 3 × CH-Ar), 8.72 (s, 1H, CH=N), 9.58 (s, 1H, OH); ^13^C-NMR (75 MHz, DMSO-*d*_6_): *δ*_C_ 22.47 (CH_3_), 56.12 (OCH_3_), 111.21, 111.51, 112.22, 113.66, 123.02, 123.34, 123.58, 125.24, 129.95 (6 × CH-Ar and 3 × C_q_-Ar) 143.67, 147.40, 153.27, 152.47, 161.64, 170.07 (CH=N, C_q_-F, C_q_=N, 2 × C_q_-O and C=O); MS (EI, 70 eV): *m*/*z* (%) [M^+^ + 1] 327.90 (11.78) for C_17_H_14_FN_3_O_3_, 311.93 (76.65), 281.67 (33.96), 228.95 (6.91), 207.57 (69.90), 177.71 (12.14), 138.21 (50.05), 101.14 (40.93), 83.47 (42.48), 66.09 (100.00).

(*E*)-6-Fluoro-2-methyl-3-((thiophen-2-ylmethylene)amino)quinazolin-4(3*H*)-one **18h**

Off-white powder; yield (75%); m.p. 159–160 °C; IR (KBr): ν_max_ (cm^−1^) 3002 (CH-Ar), 2925 (CH-aliphatic), 2372, 2342, 1676 (C=O), 1600 (CH=N and C_q_=N), 1486, 1430 (C=C), 1374, 1342 (C-F), 1302, 1272, 1243, 1134, 1188, 1134, 1040, 981, 886, 837, 814, 780, 711, 625, 596, 557, 497, 461; ^1^H-NMR (300 MHz, DMSO-*d*_6_): *δ*_H_ 2.51 (s, 3H, CH_3_), 7.30 (dd, 1H, *J* = 4.8, 3.6 Hz, CH-Ar), 7.69–7.84 (m, 4H, 4 × CH-Ar), 7.98 (d, 1H, *J =* 5.1 Hz, CH-Ar), 9.13 (s, 1H, CH=N); ^13^C-NMR (75 MHz, DMSO-*d*_6_): *δ*_C_ 22.39 (CH_3_), 111.56, 122.55, 123.09, 123.40, 128.94, 130.06, 133.43, 136.50, 143.53, 153.18, 157.27 (6 × CH-Ar, 3 × C_q_-Ar, C_q_=N and CH=N), 161.75, 163.72 (C=O and C_q_-F); MS (EI, 70 eV): *m*/*z* (%) [M^+^ + 2] 289.54 (28.27) for C_14_H_10_FN_3_OS, 264.19 (77.05), 258.21 (42.35), 235.92 (63.12), 218.42 (91.49), 210.31 (57.75), 198.35 (63.28), 186.87 (31.56), 171.33 (71.67), 162.99 (100.00), 150.01 (25.26), 138.23 (23.79), 78.89 (31.41), 41.62 (21.99).

#### 3.1.8. Synthesis of Ethyl (*Z*)-2-(3-(4-Acetoxy-3-methoxyphenyl)-2-benzamido-acrylamido)- 4-methylthiazole-5-carboxylate **20**

Equimolar quantities of (*Z*)-2-methoxy-4-((5-oxo-2-phenyloxazol-4(5*H*)-ylidene)methyl)phenyl acetate **19** [32] and ethyl 2-amino-4-methylthiazole-5-carboxylate **4** (0.01 mol) were dissolved in ethanol (30 mL), and the mixture was refluxed for 6 h. After evaporation of the excess solvent under reduced pressure, the resulting solid was recrystallized from ethanol to give the *title compound* as a buff-colored powder; yield (55%); m.p. 138–140 °C; IR (KBr): ν_max_ (cm^−1^) 3372, 3297 (2 × NH and OH), 3079 (CH-Ar), 2980 (CH-aliphatic), 1764 (COO), 1670 (br. 2 × CONH), 1598 (C=N), 1514 (br. C=C), 1373, 1278, 1123, 1092, 913, 831, 716, 589, 525, 424; ^1^H-NMR (300 MHz, DMSO-*d*_6_): *δ*_H_ 1.23 (t, 3H, *J =* 7.1 Hz, CH_3_-CH_2_), 2.26 (s, 3H, CH_3_), 3.68 (s, 3H, OCH_3_), 4.15 (AB q, 2H, *J =* 7.1 Hz, CH_2_O), 7.15 (d, 1H, *J =* 8.0 Hz, Ar-H), 7.32 (dd, 1H, *J =* 8.0, 4.0 Hz, Ar-H), 7.44 (s, 1H, C=CH), 7.49–7.62 (m, 4H, 4 × Ar-H), 7.75 (s, 1H, OH, D_2_O-exchangeable), 8.02 (d, 2H, *J =* 8.0 Hz, 2 × Ar-H), 10.17 (s, 2H, 2 × NH, D_2_O-exchangeable); ^13^C-NMR (75 MHz, DMSO-*d*_6_): *δ*_C_ 14.36, 20.35 (2 × CH_3_), 55.62 (OCH_3_), 60.65.(CH_2_), 107.54, 114.01, 122.67, 123.08, 127.02, 127.68, 128.54, 129.00, 132.00, 132.38, 132.44, 133.50, 140.18, 150.73 (9 × CH-Ar and 6 × C_q_-Ar), 162.06, 164.98, 166.31, 168.40, 170.37 (2 × C_q_-O, and 3 × C=O); MS (EI, 70 eV): *m*/*z* (%) [M^+^ + 2**]** 482.25 (29.12) for C_24_H_23_N_3_O_6_S], 413.96 (47.16), 376.30 (38.77), 340.42 (69.10), 70.38 (100.00).

#### 3.1.9. Synthesis of *N*-(2-(4-Hydroxy-3-methoxyphenyl)-1-(5-thioxo-4,5-dihydro-1,3,4-oxa-diazol-2-yl)vinyl)benzamide **22**

Carbon disulfide (1.25 mmol) and potassium hydroxide (1 mmol) were added to a solution of hydrazide **21** (1 mmol) [32] in ethanol (15 mL). The resulting reaction mixture was heated under reflux at 75 °C for 14 h. After evaporation of the excess ethanol, the remaining residue was acidified to pH 2 with HCl (37%), and the precipitated solid was collected by filtration, washed with water and recrystallized from ethanol to afford the *title compound* as a pale yellow powder; yield (55%); m.p. 190–193 °C; IR (KBr): ν_max_ (cm^−1^) 3245 (OH), 3061 (CH-Ar), 2931 (CH-aliphatic), 1652 (C=O), 1578 (C=N), 1511, 1480 (C=C), 1281 (C=S), 1169, 1030, 942, 808, 708, 616; ^1^H-NMR (300 MHz, DMSO-*d*_6_): *δ*_H_ 3.58 (s, 3H, OCH_3_), 6.82 (dd, 1H, *J =* 8.1, 4.2 Hz, CH-Ar), 7.21 (app. dd, 1H, *J =* 6.9, 1.5 Hz, CH-Ar), 7.31 (s, 1H, CH=C), 7.39 (s, 1H, CH-Ar), 7.53–7.62 (m, 3H, 3 × Ar-H), 8.03–8.19 (m, 2H, 2 × CH-Ar), 9.67, 10.26 (2s, 3H, OH and 2 × NH, D_2_O-exchangeable); ^13^C-NMR (75 MHz, DMSO-*d*_6_): *δ*_C_ 55.46 (OCH_3_), 113.64, 115.83, 116.10, 124.73, 124.85, 127.87, 128.51, 128.88, 129.44, 132.45, 133.20 (8 × CH-Ar, CH=C_q_-_arylidene_ and 3 × C_q_-Ar), 147.61, 148.92, 160.67 (C_q_-OH, C_q_-OCH_3_ and N=C_q_-O), 166.32 (C=O), 177.49 (C=S); MS (EI, 70 eV): *m*/*z* (%) [M^+^ + 2] 371.00 (28.12) for C_18_H_15_N_3_O_4_S, 352.47 (58.99), 349.42 (83.16), 297.41 (98.07), 264.22 (57.68), 137.40 (63.64), 101.49 (100.00).

#### 3.1.10. Synthesis of *N*-(1-(4-Hydroxy-3-methoxyphenyl)-3-oxo-3-(2-(phenylcarbamothionyl)hydrazinyl)prop-1-en-2-yl)benzamide **23**

Equimolar quantities of hydrazide **21** and phenyl isothiocyanate (0.003 mol) were dissolved in ethanol (15 mL) and heated under reflux for 5 h. After evaporation of the excess solvent under reduced pressure, the resulting solid was recrystallized from ethanol to afford the *title compound* as a yellow powder; yield (88%); m.p. 175–177 °C; IR (KBr): ν_max_ (cm^−1^) 3350 and 3228 (br. 4 × NH and OH), 3060 (CH-Ar), 2929 (CH-aliphatic), 1650 (2 × C=O), 1597, 1513 (C=C), 1285, 1130, 1033, 931, 814, 742, 694, 615, 499; ^1^H-NMR (300 MHz, DMSO-*d*_6_): *δ*_H_ 3.57 (s, 3H, OCH_3_), 4.50 (br. s, 1H, OH), 6.81 (d, 1H, *J =* 8.4 Hz, Ar-H), 7.08–7.17 (m, 3H, 3 × Ar-H), 7.27–7.38 (m, 5H, 5 × Ar-H), 7.50–7.63 (m, 3H, 3 × Ar-H), 7.82 (d, 1H, *J =* 7.6 Hz, Ar-H), 8.11 (d, 1H, *J =* 8.0 Hz, Ar-H), 9.32, 9.56, 9.84, 11.05 (4s, 4H, 4 × NH, D_2_O-exchangeable); ^13^C-NMR (75 MHz, DMSO-*d*_6_): *δ*_C_ 55.19 (OCH_3_), 113.26, 115.52, 123.51, 124.42, 124.68, 124.86, 125.03, 127.99, 128.16, 128.37, 130.00, 132.00, 132.50, 139.10 (13 × CH-Ar, CH-arylidene and 4 × C_q_-Ar), 147.32, 148.11 (C_q_-OH, C_q_-OCH_3_), 164.46 (C=O), 167.36 (C=O), 179.98 (C=S); MS (EI, 70 eV): *m*/*z* (%) [M^+^ + 2] 464.59 (72.07) for C_24_H_22_N_4_O_4_S, 452.30 (75.28), 437.99 (89.10), 170.48 (88.65), 164.22 (100.00), 116.83 (76.92), 99.56 (82.08)**.**

#### 3.1.11. Synthesis of *N*-(3-(2-(1-(2-((2,4-Dimethylphenyl)amino)-2-oxoethyl)-2-oxoindolin-3-ylidene)hydrazinyl)-1-(4-hydroxy-3-methoxyphenyl)-3-oxoprop-1-en-2-yl)benzamide **25**

Equimolar quantities of hydrazide **21** and *N*-(2,4-dimethyl phenyl)-2-(2,3-dioxoindolin-1-yl)acetamide **24** (0.01 mol) were dissolved in absolute ethanol (30 mL) containing catalytic amounts of glacial AcOH (3 mL) and conc. H_2_SO_4_ acid (0.5 mL), and the mixture was refluxed for 16 h. After evaporation of the excess solvent, the resulting solid was recrystallized from ethanol to afford the *title compound* as an orange powder; yield (70%); m.p. 173–175 °C; IR (KBr): ν_max_ (cm^−1^) 3384 and 3246 (3 × NH and OH), 3055 (CH-Ar), 2928, 2850 (CH-aliphatic), 1720 (CO), 1661 (3 × CO-NH), 1590 (C=N), 1515, 1466 (C=C), 1381, 1218, 1165, 1031, 917, 806, 742, 693, 616, 540; ^1^H-NMR (300 MHz, DMSO-*d*_6_): *δ*_H_ 2.17 (s, 3H, CH_3_), 2.22 (s, 3H, CH_3_), 3.76 (br. s, 1H, OH), 3.86 (s, 3H, OCH_3_), 4.65 (s, 2H, CH_2_N), 6.89–7.30 (m, 9H, 8 × Ar-H and CH=C), 7.44–7.63 (m, 3H, 3 × Ar-H), 7.90–8.20 (m, 3H, 3 × Ar-H), 8.39 (d, 1H, *J =* 008.4 Hz, Ar-H), 9.66 (s br., 2H, 2 × NH), 10.48 (s, 1H, NH); ^13^C-NMR (75 MHz, DMSO-*d*_6_): *δ*_C_ 17.89, 20.58 (2 × CH_3_), 42.02 (CH_2_N), 55.59 (OCH_3_), 109.03, 115.77, 115.98, 117.45, 122.00, 122.15, 125.19, 125.50, 126.00, 126.62, 127.09, 127.50, 128.39, 128.80, 129.32, 131.00, 132.00, 133.30, 134.50, 134.70, 139.75, 143.45 (15 × CH-Ar, CH-_arylidene_ and 8 × C_q_-Ar), 147.91, 150.31 (C_q_-OH, C_q_-OCH_3_), 159.12, 161.06, 165.64, 170.15 (4 × C=O); MS (EI, 70 eV): *m*/*z* (%) [M^+^] 617.31 (20.41) for C_35_H_31_N_5_O_6_ 435.23 (39.63), 332.70 (71.87), 216.41 (100.00), 140.10 (46.12), 112.55 (54.91), 102.09 (53.22).

### 3.2. Evaluation of Biological Activities

Detailed procedures are available in Supplementary Materials.

#### 3.2.1. In Vitro DPPH Radical Scavenging Assay

The antioxidant efficiencies of target compounds were determined using the 1,1-diphenyl-2-picrylhydrazyl (DPPH) radical scavenging method as detailed by Bersuder et al. [36].

#### 3.2.2. In Vitro PDK-1 Inhibition Assay

Principally, the PDK-1 inhibitory activities were evaluated using the non-radioactive Kinase-Glo™ Luminescent Kinase Kit (Promega Corporation, Madison, WI, USA) following the instructions of the manufacturer.

#### 3.2.3. In Vitro LDHA Inhibitory Assay

The LDHA inhibitory efficiencies were investigated on Spectramax M2 spectrofluorometer via measuring the amounts of the consumed NADH^40^ using an NAD^+^/NADH quantification kit.

#### 3.2.4. Cell Culture and Viability Assay

The antiproliferative activities of the newly synthesized compounds **3a–e**, **8**, **9a–c**, **13**, **14**, **17**, **18a–h**, **20**, **22**, **23** and **25** were examined on human colon HCT-116 and LoVo cell lines as well as HUVEC (American Type Culture Collection, Manassas, VA, USA).

#### 3.2.5. Cell Cycle Assay

Flow cytometric analysis using a Propidium Iodide Flow Cytometry Kit for Cell Cycle Analysis (Abcam, Cambridge, UK), was used in order to analyze the changes in cell cycle distribution induced in HCT 116 and LoVo cultures upon treatment with compound **3d**.

#### 3.2.6. Annexin-V-FITC Assay

An Annexin V-FITC Apoptosis Detection Kit (BioVision Research Products, Mountain View, CA, USA) was used to analyze the distribution of early and late apoptotic cells, as well as necrotic cells after treatment with compound **3d**.

#### 3.2.7. Analysis of Reactive Oxygen Species (ROS) Levels

A Human ROS ELISA Kit was used to measure the intracellular accumulation of ROS in the **3d**-treated LoVo and HCT-116 cells.

#### 3.2.8. Mitochondrial Transmembrane Potential (MMP) Measurement

The mitochondrial transmembrane potential was measured using a tetramethylrhodamine, ethyl ester (TMRE) mitochondrial membrane potential assay kit.

#### 3.2.9. Quantification of the Expression Levels of *Bax*, *Bcl-2* and *Caspase-3* Genes

The expression levels of these genes were determined using a quantitative real-time PCR technique.

### 3.3. In Silico Studies

#### 3.3.1. Molecular Docking Simulations

Protein structure preparation*:* Molecular docking simulations were conducted using AutoDock Vina 1.1.2. [73,74]. The required protein files in PDB format were downloaded from the Protein Data Bank ((https://www.rcsb.org/) [75]. The crystal structure of lactate dehydrogenase A (LDHA) was obtained using PDB ID 4ZVV [55], which is co-crystallized with the ligand GN0 ((2*R*)-5-(2-chlorophenyl)sulfanyl-2-(4-morpholin-4-ylphenyl)-4-hydroxy-2-thiophen-3-yl-1,3-dihydropyridin-6-one). For pyruvate dehydrogenase kinase isoform 1 (PDK-1), the structure with PDB ID **2Q8G** was selected, co-crystallized with AZX ligand; 4-[3-chloro-4-[[(2*R*)-3,3,3-trifluoro-2-hydroxy-2-methyl-propanoyl]amino] phenyl]sulfonyl-*N*,*N*-dimethyl-benz-amide) was used [56]. For both structures, protein preparation was performed using MGLTools 1.5.7. as follows: All water molecules were removed from the protein structures to eliminate potential interference with the docking process. Polar hydrogen atoms were added to account for potential hydrogen bonding interactions. Kollman united atom charges were assigned to all atoms in the protein structures.

The co-crystallized ligands were removed to define and expose the binding site location. Non-polar hydrogens were merged, and Gasteiger partial atomic charges were calculated and added. The prepared protein structures were then saved in PDBQT format as required for AutoDock Vina calculations [76].

Ligand preparation: Ligand structures were initially sketched using MarvinSketch 19.17.0 software (ChemAxon, Budapest, Hungary) and then saved in MOL format. Geometry optimization and energy minimization of ligands were performed using MMFF94 force field in Open Babel v3.1.1 before conversion to PDBQT format using MGLTools 1.5.7. Each ligand was optimized as follows: All non-polar hydrogen atoms were merged, and Gasteiger partial charges were calculated and assigned to each atom. Rotatable bonds were defined to allow flexibility during the docking process. Energy minimization was performed using the universal force field (UFF) with 5000 steps of steepest descent followed by conjugate gradient minimization until the energy difference between steps was less than 0.1 kcal/mol. The optimized ligands’ structures were saved in the required pdbqt format for subsequent docking studies.

Docking calculations: Molecular docking simulations were performed using AutoDock Vina 1.1.2. [73,74]. For LDHA (4ZVV). The grid box was centered on the GN0 binding site (cocrystal ligand) with dimensions of 25 × 25 × 25 Å to ensure complete coverage of the binding pocket. Similarly, for PDK-1 (2Q8G), the grid box was centered on the binding site of AZX with identical dimensions. The grid spacing was set to 0.375 Å for both proteins. The docking parameters were configured as follows: Exhaustiveness was set to 32 to balance accuracy and computational time, energy range was set to a maximum of 4 kcal/mol, and the number of binding modes was set to 9. The search algorithm was set to employ the default Broyden–Fletcher–Goldfarb–Shanno (BFGS) method for local optimization. For each ligand–protein pair, a minimum of three independent docking runs were performed to ensure reproducibility of the results.

Visualization of analysis: Docked poses and protein–ligand interactions were visualized and analyzed using BIOVIA Discovery Studio Visualizer v21.1.0.20298 [77].

#### 3.3.2. Physicochemical, Drug-Likeness and ADMET Studies

The physicochemical, drug-likeness and ADMET predictions were carried out by using the free webserver [59] ADMETlab 2.0 (https://admetmesh.scbdd.com/), which was accessed on 7 February 2024; the tested compounds were imported through their coding smiles.

## 4. Conclusions

A series of 24 novel nitrogen-, oxygen-, and sulfur-containing heterocyclic derivatives were synthesized and evaluated for their antioxidant potential, PDK-1 and LDHA inhibitory activity, and cytotoxic effects against LoVo and HCT-116 colon carcinoma cell lines, alongside safety profiling in normal HUVECs. Among them, compounds **3b** and **3d** emerged as the most promising multi-target agents. Compound **3b** exhibited strong dual inhibition of PDK-1 and LDHA, while **3d** demonstrated potent DPPH radical scavenging activity and LDHA inhibition. Molecular docking simulations confirmed the high binding affinities of both compounds to the active sites of LDHA and/or PDK-1, supporting the observed biochemical inhibition. In cytotoxicity assays conducted at 100 µg/mL for 48 h, compounds **3b** and **3d** significantly reduced the viability of LoVo and HCT-116 colon cancer cells below 29%, while preserving approximately 90% viability in normal HUVECs. Their IC_50_ values ranged from 156.60 to 190.30 µM across the two colon cancer cell lines. The antitumor effects of compound **3d** (30 µg/mL, 48 h) were mechanistically linked to cell cycle arrest (G2/M in LoVo and G1 in HCT-116) and apoptosis induction, as evidenced by increased ROS generation, disruption of mitochondrial membrane potential, downregulation of *Bcl-2*, and upregulation of *Bax* and *caspase-3*. In silico ADMET predictions revealed moderate to high lipophilicity and poor aqueous solubility for both compounds, particularly **3b**, suggesting the need for structural optimization to improve solubility and oral bioavailability. Notably, both **3b** and **3d** showed only one violation of Lipinski’s rule of five (i.e., LogP), and neither contained PAINS-associated substructures, supporting their drug-likeness and the validity of their bioactivities. Taken together, these findings position compounds **3b** and **3d** as promising lead candidates for further development as multi-target, anti-colorectal cancer agents.

## Data Availability

The data presented in this study are available in the Section 3 and the Supplementary Materials.

## Supplementary Materials

The following supporting information can be downloaded at: https://www.mdpi.com/article/10.3390/ph18060801/s1, Table S1: Energy minimization calculations for compounds **3a**–**s**, **9a**–**c**, 1**8a**–**h**, **20**, **21**, **22**, **23** and **25**; Table S2: Evaluation of DPPH radical scavenging activity of the newly synthesized compounds **3a**–**e**, **8**, **9a**–**c**, **13**, **14**, **17**, **18a**–**h**, **20**, **22**, **23 **and **25**. Data expressed are as IC_50_ values (mM) ± SD from three independent experiments. BHT was used as reference antioxidant; Table S3. The mean inhibitory efficiencies (%) of the newly synthesized compounds **3a**–**e**, **8**, **9a**–**c**, **13**, **14**, **17**, **18a**–**h**, **20**, **22**, **23 **and **25**; against PDK-1 and LDHA, determined at a concentration of 100 µg/mL ± SD from three replicates. Sodium oxamate (SO), and sodium dichloroacetate (SDA), both at 1000 µM, were used as the reference inhibitors for LDHA and PDK-1, respectively. The mean half-maximal inhibitory concentrations were calculated for compounds showing >80% inhibition of LDHA and/or PDK-1. Results are expressed in µM ± SD from three independent experiments; Table S4: Cytotoxic effects of the newly synthesized compounds **3a**–**e**, **8**, **9a**–**c**, **13**, **14**, **17**, **18a**–**h**, **20**, **22**, **23**, and **25** on LoVo and HCT-116 colon carcinoma cells, as well as HUVECs. Results are presented as the mean percentage of viable cells remained after 48 h exposure to 100 µg/mL of each compound, based on LDH release assay data from three independent determinations ± SD. Assay medium and 0.1% Triton X-100 were used as negative and positive controls, respectively; Table S5: Mean half-maximal inhibitory concentrations (IC_50_ values) of compounds that reduced cell viability to less than 41.00% in HCT-116 and/or LoVo colon carcinoma cells. Results are expressed in µM ± SD from three independent determinations. 5-Fluorouracil (5-FU) was used as the reference drug; Table S6: Reactive oxygen species (ROS) production levels in HCT-116 and LoVo colon carcinoma cells following treatment with 30 µg/mL of compound **3d** for 48 h. Results are expressed as mean values (pg/mL) ± SD from two independent experiments. Table S7: Primer sequences for *Bax*, *Bcl-2*, *Caspase-3*, and *GAPDH* (control). Section S3.2. Evaluation of Biological Activities. S3.2.1. In vitro DPPH radical scavenging assay; S3.2.2. In vitro PDK-1 inhibition assay; S3.2.3. In vitro LDHA inhibitory assay; S3.2.4. Cell culture and viability assay; S3.2.5. Cell cycle assay; S3.2.6. Annexin-V-FITC assay; S3.2.7. Analysis of reactive oxygen species (ROS) levels; S3.2.8. Mitochondrial transmembrane potential (MMP) measurement; S3.2.9. Quantification of the expression levels of *Bax*, *Bcl-2* and *Casp-3* genes, and ^1^H-NMR and ^13^C-NMR spectra of some selected Examples.
