# Supplementary material for: New Nitrogen-, Oxygen-, and Sulfur-Containing Heterocyclic Compounds as Anti-Colon Cancer Agents: Synthesis, Multitargeted Evaluations, Molecular Docking Simulations and ADMET Predictions
Source: Pharmaceuticals (Basel). 2025 May 27;18(6):801. doi: 10.3390/ph18060801 (PMC12196476; doi:10.3390/ph18060801)

New Nitrogen-, Oxygen-, and Sulfur-Containing Heterocyclic Compounds as Anti-Colon Cancer Agents: Synthesis, Multitargeted Evaluations, Molecular Docking Simulations and ADMET Predictions

Nahed Nasser Eid El-Sayed [1,*], Najeh Krayem [2], Hamed Ahmed Derbala [3], Shimaa Kamal [3], Syde Nasir Abbas Bukhari [4], Mohamed K. El-Ashrey [5,6], Zainab M. Almarhoon [7], Seham Soliman Alterary [7] and Abir Ben Bacha [8*]

[1] Egyptian Drug Authority, 51 Wezaret El-Zerra St., Giza 35521, Egypt
[2] Laboratoire de Biochimie et de Génie Enzymatique des Lipases, ENIS, Université de Sfax, Route de Soukra 3038, Sfax BP 1173, Tunisia; krayemnajeh@yahoo.fr
[3] Chemistry Department, Faculty of Science, Ain Shams University, Abbassia, Cairo 11566, Egypt; hamed_derbala@sci.asu.edu.eg (H.A.D.); dr.shimo4189@yahoo.com (S.K.)
[4] Department of Pharmaceutical Chemistry, College of Pharmacy, Jouf University, Sakaka 72388, Saudi Arabia; sbukhari@ju.edu.sa
[5] Pharmaceutical Chemistry Department, Faculty of Pharmacy, Cairo University, Kasr Elini St., 11562, Cairo, Egypt; mohamed.elashrey@pharma.cu.edu.eg
[6] Medicinal Chemistry Department, Faculty of Pharmacy, King Salman International University, 46612, South Sinai, Egypt
[7] Department of Chemistry, College of Science, King Saud University, P.O. Box 2455, Riyadh 11451, Saudi Arabia; zalmarhoon@ksu.edu.sa; salterary@ksu.edu.sa (S.S.A.)
[8] Biochemistry Department, College of Sciences, King Saud University, P.O. Box 22452, Riyadh 11495, Saudi Arabia
* Correspondence: nahed.elsayed@edaegypt.gov.eg (N.N.E.E.-S.); aalghanouchi@ksu.edu.sa (A.B.B.)

Supplementary Materials

Table S1. Energy minimization calculations for compounds **3a–e**, **9a–c**, **18a–h**, **20**, **21**, **22**, **23**, and **25**.

***E*-isomer**	***Z*-isomers**
**3a**<br>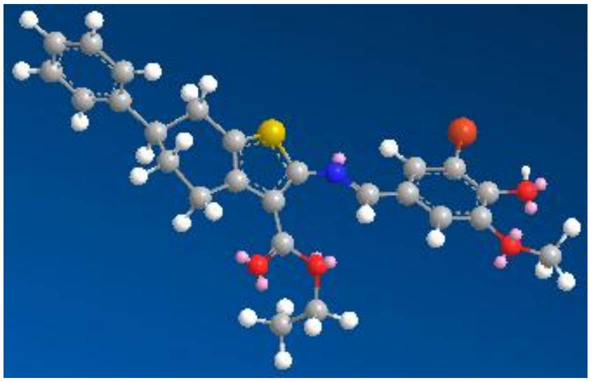<br>**<u>Total Energy: 55.9270 kcal/mol</u>**	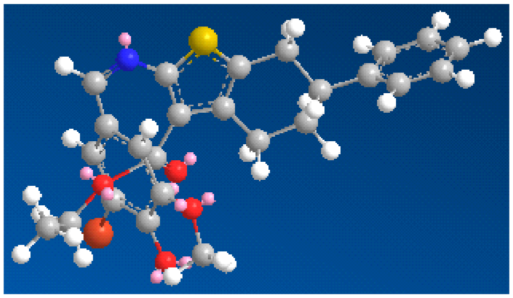<br>**<u>Total Energy: 754.2032 kcal/mol</u>**
**3b**<br>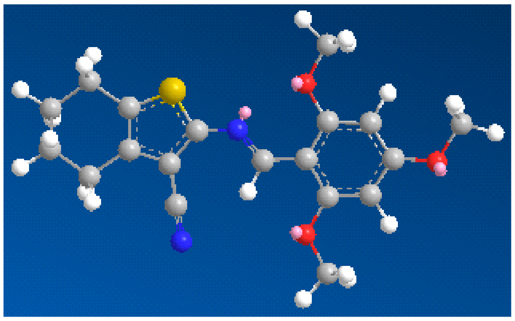<br>**<u>Total Energy: 49.9832 kcal/mol</u>**	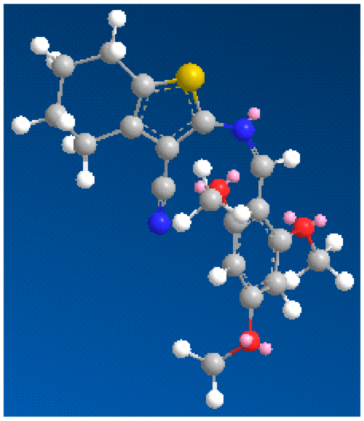<br>**<u>63.7157 kcal/mol Total Energy</u>**
**3c**<br>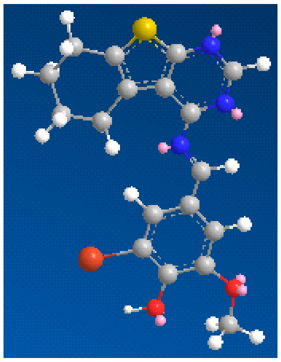<br>**<u>Total Energy: 34.8187 kcal/mol</u>**	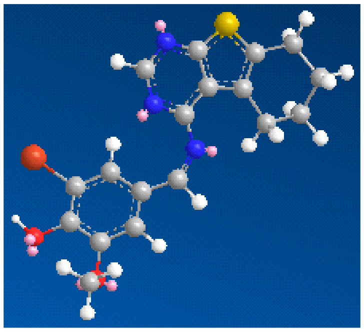<br>**<u>Total Energy: 47.1526 kcal/mol</u>**

**3d** 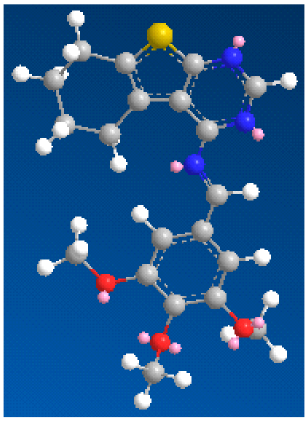 **Total Energy: 46.2193 kcal/mol**	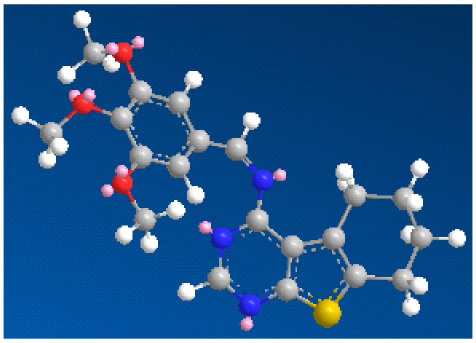 **Total Energy: 61.0695 kcal/mol**
**3e** 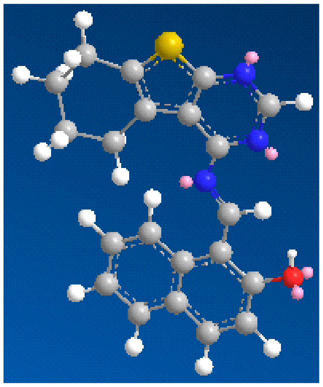 **Total Energy: 36.8572 kcal/mol**	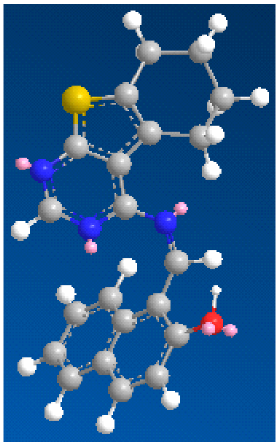 **Total Energy: 48.3510 kcal/mol**
**9a** 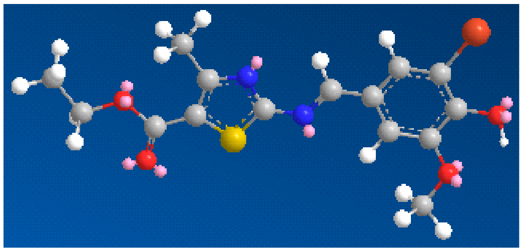 **Total Energy: 29.5580 kcal/mol**	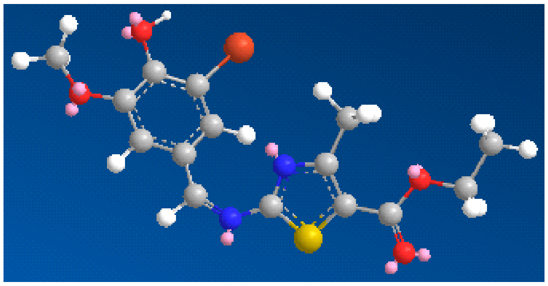 **Total Energy: 45.7924 kcal/mol**
**9b** 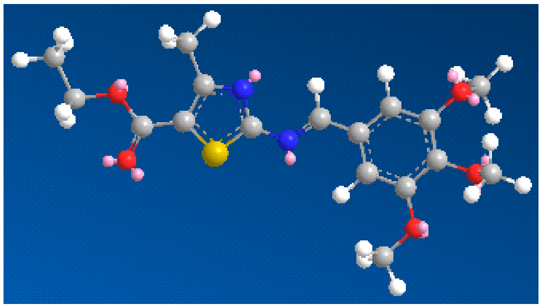 **Total Energy: 45.7132 Kal/mol**	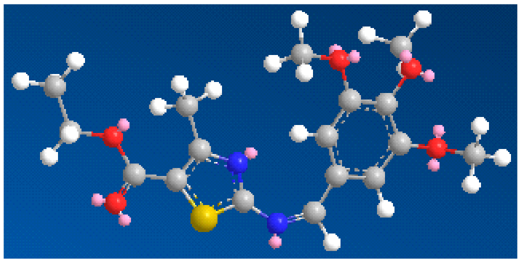 **Total Energy: 57.3441 kcal/mol**

9c<br>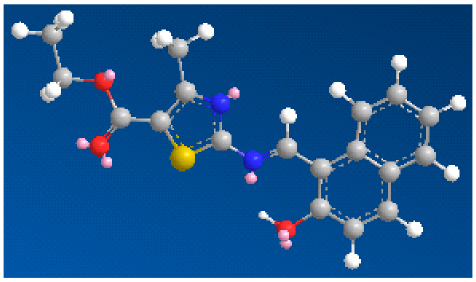<br>Total Energy: 30.8139 kcal/mol	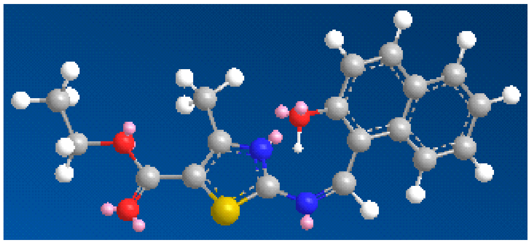<br>Total Energy: 45.7724 kcal/mol
18a<br>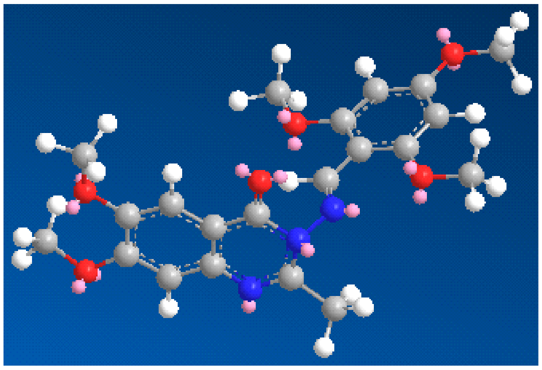<br>Total Energy: 57.3767 kcal/mol	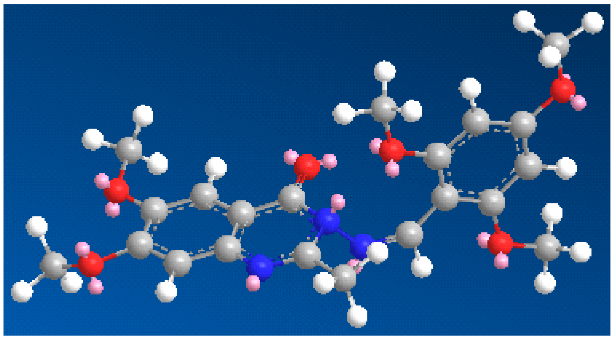<br>Total Energy: 57.3936 kcal/mol
18b<br>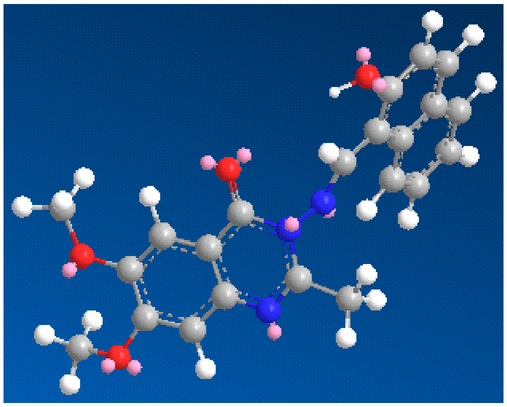<br>Total Energy: 30.4318 kcal/mol	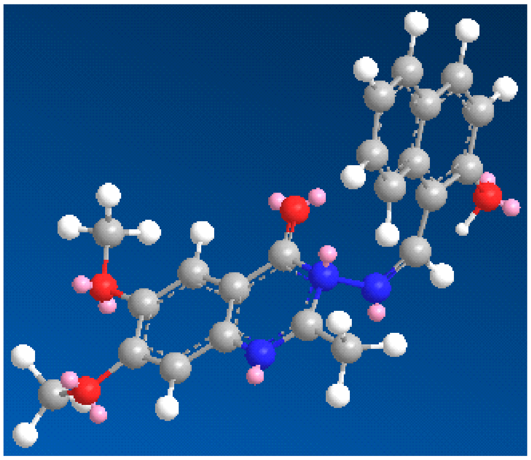<br>Total Energy: 41.7276 kcal/mol

18c 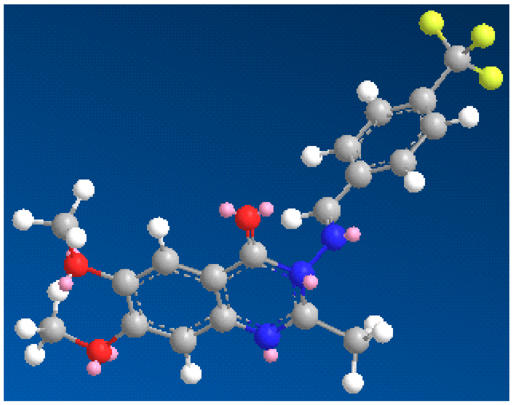 **Total Energy: 33.7195 kcal/mol**	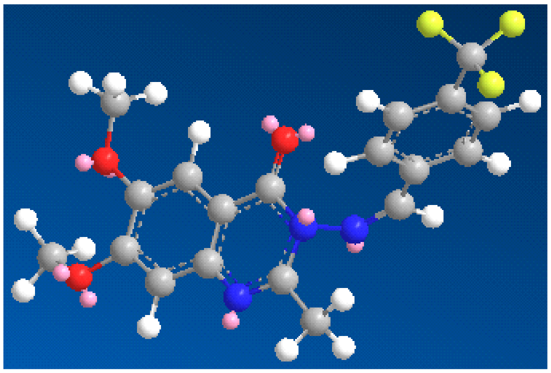 **Total Energy: 34.5982 kcal/mol**
18d 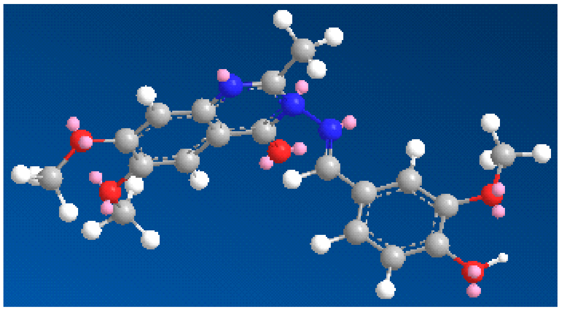 **Total Energy: 27.9704 kcal/mol**	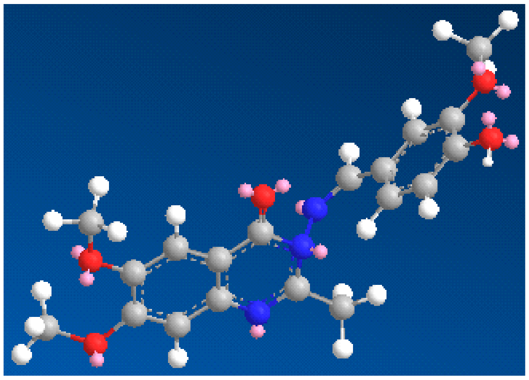 **Total Energy: 33.3043 kcal/mol**
18e 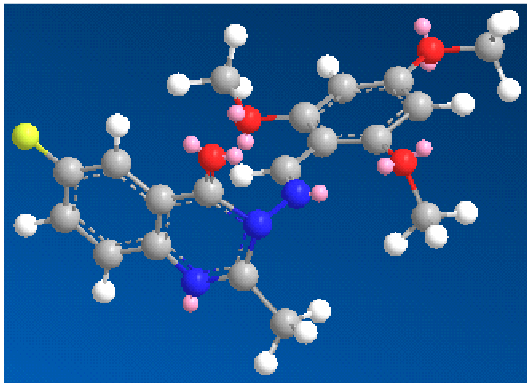 **Total Energy: – 31.6658 kcal/mol**	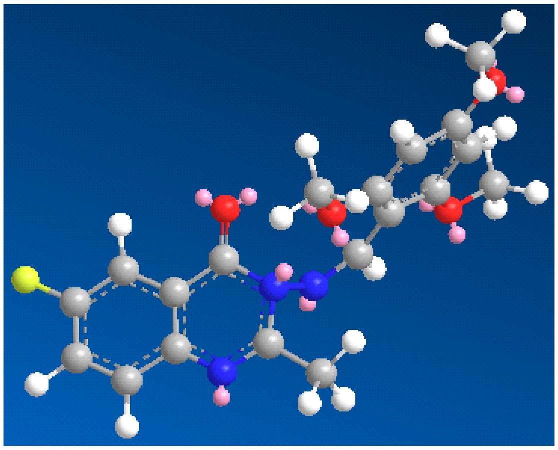 **Total Energy: 45.0155 kcal/mol**

18f 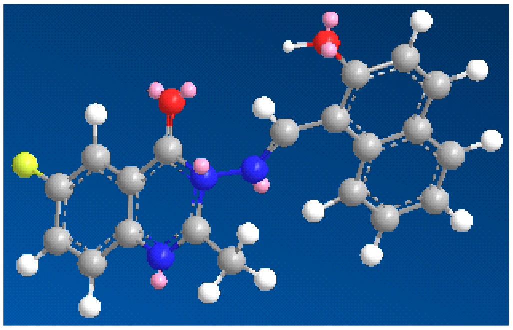 Total Energy: 18.1044 kcal/mol	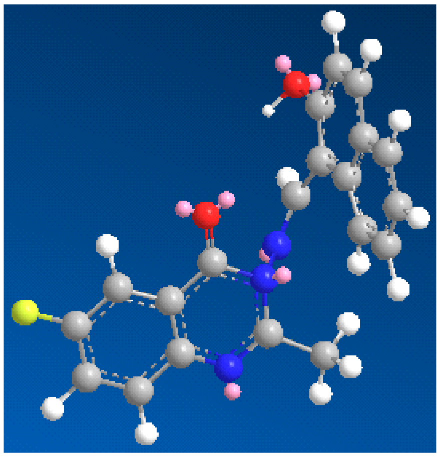 Total Energy: 29.4190 kcal/mol
18g 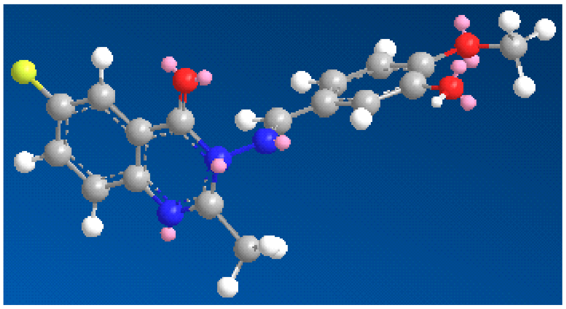 Total Energy: 20.6756 kcal/mol	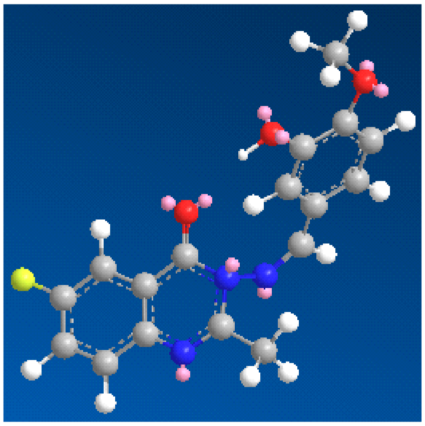 Total Energy: 21.5053 kcal/mol
18h 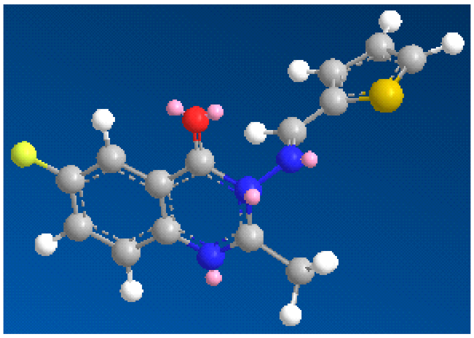 Total Energy: 27.9994 kcal/mol	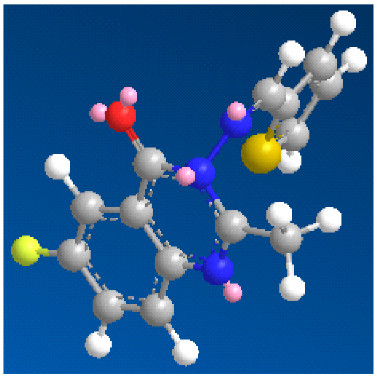 Total Energy: 29.0530 kcal/mol

<table>
<tr>
<td>20
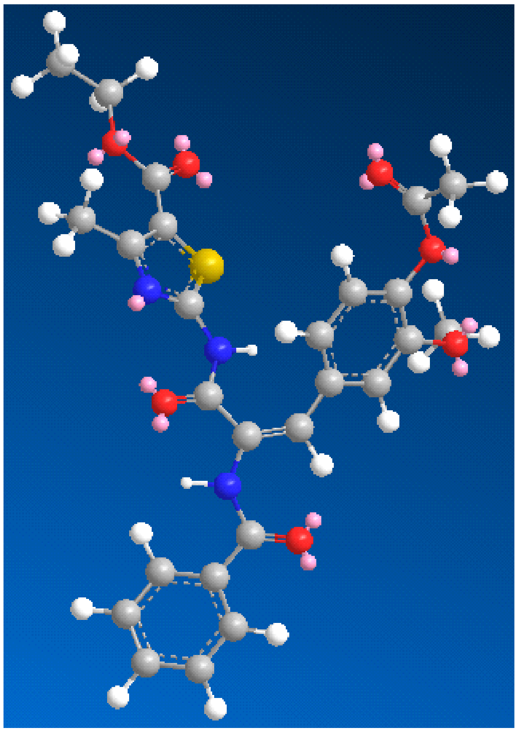
<u>Total Energy: 42.2314 kcal/mol</u></td>
<td>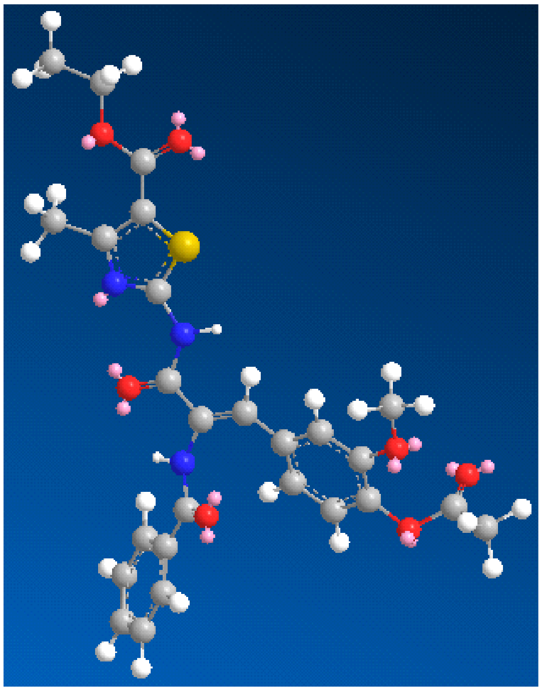
<u>Total Energy: 41.1108 kcal/mol</u></td>
</tr>
<tr>
<td>21
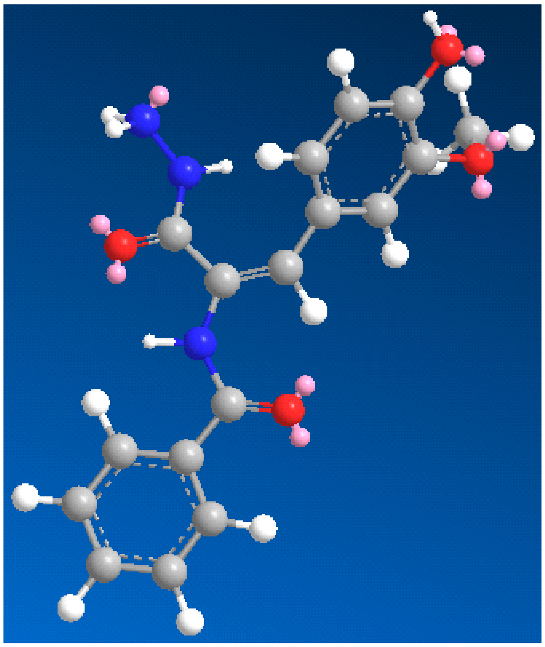
<u>Total Energy: 12.4754 kcal/mol</u></td>
<td>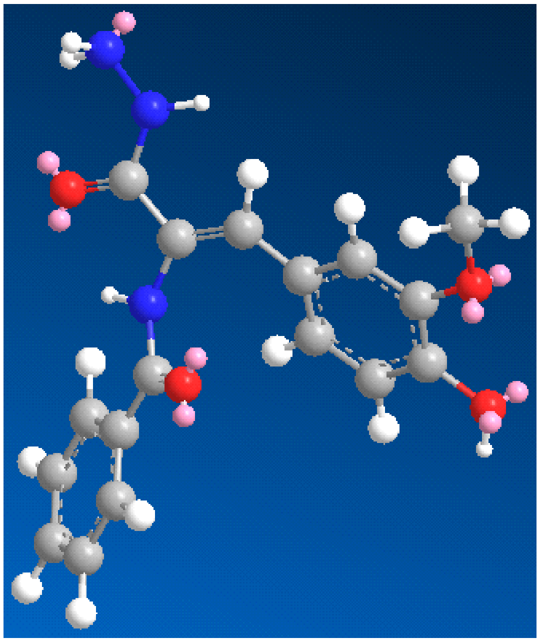
<u>Total Energy: 10.7629 kcal/mol\</u></td>
</tr>
</table>

22<br>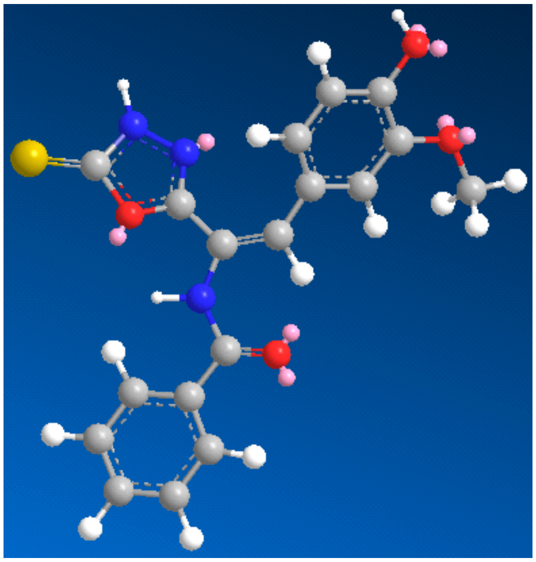<br>**Total Energy: 34.8653 kcal/mol**	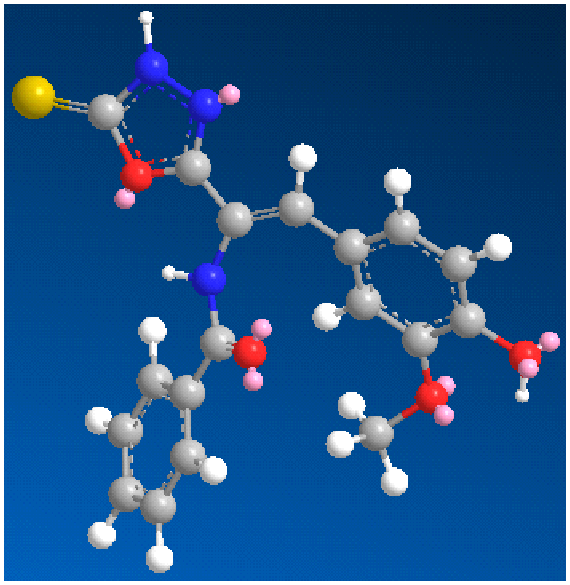<br>**Total Energy: 28.8336 kcal/mol**
23<br>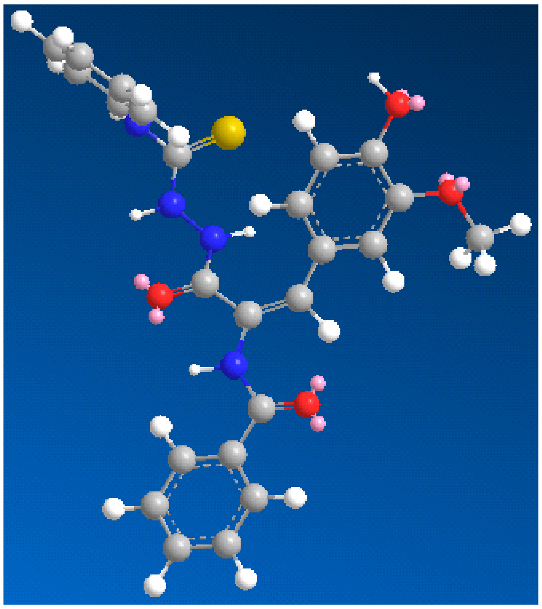<br>**Total Energy: 5.9225 kcal/mol**	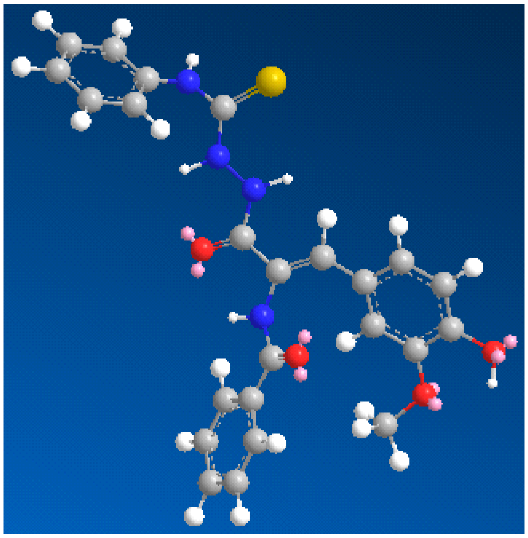<br>**Total Energy: 0.9826 kcal/mol**

<table>
<tr><td>25
(*Z*,*E*)
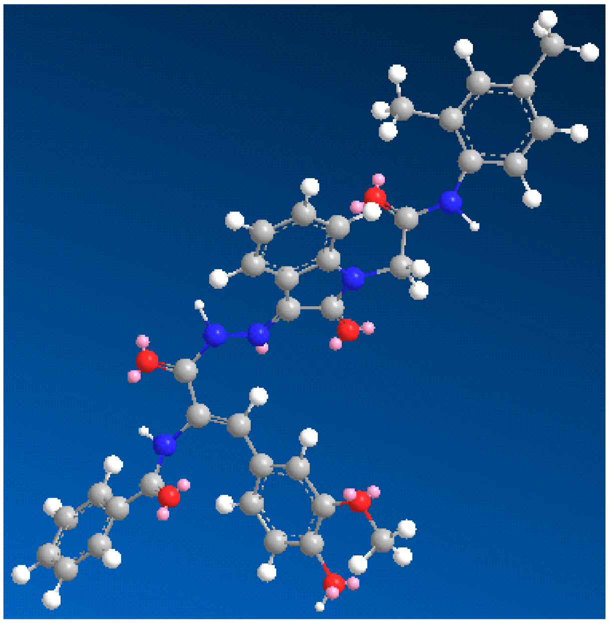
Total Energy: 30.1796 kcal/mol</td><td>(*Z*,*Z*)
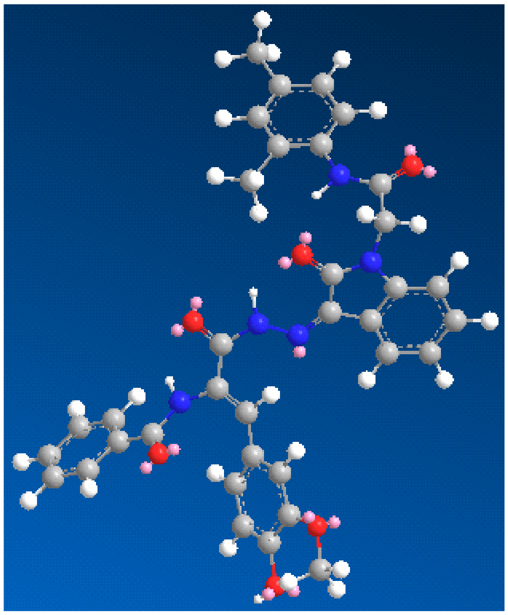
Total Energy: 14.5928 kcal/mol</td></tr>
</table>

Table S2. Evaluation of DPPH radical scavenging activity of the newly synthesized compounds **3a–e**, **8**, **9a–c**, **13**, **14**, **17**, **18a–h**, **20**, **22**, **23**, and **25**. Data are expressed as IC_{50} values (mM) ± SD from three independent experiments. BHT was used as reference antioxidant.

**Comp. #**	**Mean IC_{50} (mM) for initial DPPH radicals ± SD**
**3a**	0.214 ± 0.027
**3b**	0.522 ± 0.025
**3c**	0.476±0.038
**3d**	0.164 ± 0.008
**3e**	0.890±0.039
**8**	1.517±0.070
**9a**	1.553 ± 0.035
**9b**	1.811 ± 0.115
**9c**	0.881 ± 0.041
**13**	1.820 ± 0.188
**14**	0.327 ±0.007
**17**	0.307 ±0.010
**18a**	1.384 ± 0.039
**18b**	0.188 ± 0.010
**18c**	0.606 ± 0.015
**18d**	0.658 ± 0.016
**18e**	0.781 ± 0.046
**18f**	0.288 ± 0.029
**18g**	0.238 ± 0.006
**18h**	0.379 ± 0.004
**20**	0.745 ± 0.027
**22**	0.650 ± 0.076
**23**	1.211 ± 0.030
**25**	0.162 ± 0.023
**BHT**	0.245 ± 0.027

Table S3. Mean inhibitory efficiencies (%) of the newly synthesized compounds **3a–e**, **8**, **9a–c**, **13**, **14**, **17**, **18a–h**, **20**, **22**, **23**, and **25** against PDK-1 and LDHA, determined at a concentration of 100 μg/mL ± SD from three replicates. Sodium dichloroacetate (SDA) and Sodium oxamate (SO), both at 1000 μM, were used as the reference inhibitors for PDK-1 and LDHA, respectively. The mean half-maximal inhibitory concentrations were calculated for compounds showing >80% inhibition of PDK-1 and/or LDHA. Results are expressed in μM ± SD from three independent experiments.

Comp. #	Mean Inhibition Percent ± SD		Mean IC_{50} μM ± SD	
	PDK-1	LDHA	PDK-1	LDHA
**3a**	25.15 ± 3.04	25.75 ± 2.48		
**3b**	94.65 ± 2.33	82.75 ± 3.18	147.29 ± 5.95	166.79 ± 10.11
**3c**	7.60 ± 0.85	40.00 ± 1.41		
**3d**	41.80 ± 3.39	85.90 ± 2.97	305.11 ± 11.07	149.03 ± 7.93
**3e**	31.25 ± 3.18	28.30 ± 1.84		
**8**	16.55 ± 2.19	23.25 ± 1.06		
**9a**	26.85 ± 2.62	40.55 ± 2.19		
**9b**	30.10 ± 1.56	33.40 ± 1.98		
**9c**	48.75 ± 1.77	67.35 ± 3.32		
**13**	40.55 ± 2.76	40.25 ± 3.18		
**14**	80.50 ± 4.95	37.00 ± 2.83	192.08 ± 5.57	378.01 ± 3.98
**17**	91.00± 2.83	5.75 ± 0.35	168.86 ± 1.81	1059.71 ± 7.64
**18a**	66.67 ± 1.53	16.55 ± 0.78		
**18b**	72.67 ± 2.08	58.55 ± 3.89		
**18c**	44.67 ± 1.53	21.10 ± 0.99		
**18d**	65.00 ± 3.46	12.20 ± 1.98		
**18e**	30.00 ± 1.00	36.75 ± 1.91		
**18f**	52.00± 3.61	14.40 ± 1.41		
**18g**	66.00±1.73	22.20 ± 1.13		
**18h**	37.00±3.61	18.85 ± 1.77		
**20**	28.25±1.06	16.90 ± 1.27		
**22**	39.50±2.12	27.25 ± 2.48		
**23**	11.00±1.41	34.35 ± 3.32		
**25**	13.30±1.84	40.50 ± 3.54		
**SDA**	100	-----------	170.62 ± 7.03	
**SO**	---------------	100		140.50 ± 7.64

Table S4. Cytotoxic effects of the newly synthesized compounds **3a–e**, **8**, **9a–c**, **13**, **14**, **17**, **18a–h**, **20**, **22**, **23**, and **25** on LoVo and HCT-116 colon carcinoma cells, as well as HUVECs. Results are presented as the mean percentage of viable cells remained after 48 h exposure to 100 μg/mL of each compound, based on LDH release assay data from three independent determinations ± SD. Assay medium and 0.1% Triton X-100 were used as negative and positive controls, respectively.

Comp. #	Mean % of viable cells ± SD		
	**LoVo**	**HCT-116**	**HUVEC**
**3a**	57.67 ± 1.53	60.67 ± 3.06	85.00 ± 1.73
**3b**	28.33 ± 1.41	20.67 ± 2.08	90.67 ± 1.15
**3c**	95.67 ± 1.53	89.33 ± 4.16	93.00 ± 2.65
**3d**	19.67 ± 2.83	21.67 ± 2.08	90.00 ± 2.00
**3e**	62.33 ± 2.52	57.00 ± 1.73	96.67 ± 1.16
**8**	13.50 ± 0.71	26.50 ± 2.12	84.63 ± 2.54
**9a**	65.33 ± 3.54	72.00 ± 2.65	70.33 ± 1.53
**9b**	60.33 ± 2.83	54.00 ± 4.58	81.67 ± 2.52
**9c**	60.33 ± 2.12	64.33 ± 3.06	80.67 ± 3.22
**13**	86.50 ± 2.12	71.50 ± 2.12	84.00 ± 2.33
**14**	13.00 ± 1.14	12.50 ± 0.71	95.00 ± 1.82
**17**	10.50 ± 0.71	20.50 ± 2.12	96.00 ± 2.56
**18a**	48.33 ± 2.83	40.67 ± 4.16	85.00 ± 1.00
**18b**	63.00 ± 2.65	58.33 ± 2.08	85.33 ± 3.06
**18c**	63.00 ± 3.00	71.33 ± 6.51	91.33 ± 2.08
**18d**	30.67 ± 4.24	38.00 ± 2.00	86.33 ± 1.53
**18e**	88.67 ± 3.21	87.33 ± 2.08	83.00 ± 3.61
**18f**	73.00 ± 3.46	83.00 ± 3.00	83.00 ± 1.73
**18g**	74.67 ± 2.52	58.67 ± 3.06	88.00 ± 2.65
**18h**	33.00 ± 4.24	37.00 ± 2.00	78.00 ± 2.00
**20**	51.00 ± 2.83	49.00 ± 2.83	73.00 ± 2.75
**22**	29.50 ± 2.12	40.50 ± 3.54	79.00 ± 2.00
**23**	57.50 ± 2.12	23.00 ± 2.83	80.00 ± 1.92
**25**	71.50 ± 3.54	79.00 ± 2.83	87.94 ± 2.33
**0.1%Triton-X100**	0.00	0.00	Not determined
**Assay medium**	99.50 ± 0.71	100.00 ± 0.00	Not determined

Table S5. Mean half-maximal inhibitory concentrations (IC_{50} values) of compounds that reduced cell viability < 41.00% in HCT-116 and/or LoVo colon carcinoma cells. Results are expressed in μM ± SD from three independent determinations. 5-Fluorouracil (5-FU) was used as the reference drug.

Comp. #	Mean IC_{50} Values in μM ± SD	
	**LoVo cells**	**HCT-116 cells**
**3b**	190.30 ± 5.67	170.21 ± 9.85
**3d**	156.60 ± 5.22	160.96 ± 5.43
**8**	320.35± 7.44	409.99 ± 13.47
**14**	292.09 ± 1.17	293.35 ± 5.38
**17**	277.00 ± 0.21	302.22 ± 10.47
**18a**	132.23 ± 7.40	210.43 ± 2.42
**18d**	202.15 ± 7.82	238.24 ± 8.12
**18h**	479.17 ± 19.18	497.72 ±12.57
**22**	437.33 ± 1.51	510.84 ± 5.61
**23**	444.39 ± 9.17	215.39 ± 1.25
**5-FU**	19.99 ± 3.21	26.98 ± 1.87

Table S6. Reactive oxygen species (ROS) production levels in HCT-116 and LoVo colon carcinoma cells following treatment with 30 μg/mL of compound **3d** for 48 h. Results are expressed as mean values (pg/mL) ± SD from two independent experiments.

Comp.#	[ROS] pg/mL ± SD	Fold of control
**Control LoVo**	168.6 ± 6.5	1
**3d/LoVo**	434.5 ± 20.3	2.6
**Control HCT-116**	143.5 ± 6.8	1
**3d/HCT-116**	290.1 ± 12.7	2.0

S3.2. Evaluation of Biological Activities.

S3.2.1. In vitro DPPH radical scavenging assay.

The antioxidant efficiencies of compounds—**3a**–**e**, **8**, **9a**–**c**, **13**, **14**, **17**, **18a**–**h**, **20**, **22**, **23**, and **25**—were determined using the 1,1-diphenyl-2-picrylhydrazyl (DPPH) radical scavenging method as detailed by Bersuder *et al.* [36]. Fundamentally, the purple color of the stabilized DPPH radical fades in the presence of an antioxidant, thus, the change of absorbance can be followed spectrophotometrically at 519 nm. In the assay, equal volumes (0.5 mL) of DPPH ethanolic solution and each compound (0-2 mg/mL in DMSO) or butylated hydroxytoluene (BHT) as the reference were mixed well by shaking. The negative control consisted of the same amount of DMSO and DPPH solution. After, incubation all mixtures at room temperature in the dark for one hour, the absorbance of the residual DPPH radicals was measured at 519 nm using the UV‑vis spectrophotometer. The DPPH radical scavenging activity of each compound (%) was calculated using the formula:

Scavenging activity = $(1 - A_{compound}/A_{Control}) * 100$, where $A_{compound}$ and $A_{control}$ the absorbance values of each tested compound (or BHT) and the negative control, respectively. The half maximal inhibitory concentration (IC_{50}) for each compound in mg/mL was deduced by graphing the dose-response curve relating the scavenging activity (%) to the compound concentration. Data points represent the mean ± standard deviation (SD) of three independent determinations.

S3.2.2. In vitro PDK-1 inhibition assay.

The PDK-1 inhibitory activities of the test compounds were evaluated using the non-radioactive Kinase-Glo™ Luminescent Kinase kit (Promega Corporation, Madison, WI, USA), following the manufacturer's protocol. Briefly, 5 µL of assay buffer—containing 10 × 250 mM Tris-HCl, 50 mM $MgCl_2$, 5 mM ethyleneglycol-bis(β-aminoethylether)-*N*,*N*,*N'*,*N'*-tetraacetic acid (EGTA), 10 mM ethylenedi- aminetetraacetic acid (EDTA), and 10 mM dithiothreitol (DTT)—was added to each well of a 96-well plate, along with 25 µL of distilled water. Subsequently, 5 µL of each test compound at various concentrations (50, 100, 200 and 300 µg/mL in 1% DMSO) was added to the respective wells, except for the two control wells, which received 5 µL of either PBS buffer or 1% DMSO. Then, 5 µL of pyruvate dehydrogenase E1 **(**PDH E1) protein (10 µM) and 5 µL of ATP (10 µM) were added to each well. To initiate phosphorylation, 5 µL of PDK-1 (20 µM) in protein buffer (1 mM $MgCl_2$, 2000 mM KCl, 40 mM K_3PO_4) was added to all the wells except the control wells, which received 5 µL of protein buffer instead. The plate was then mixed thoroughly and incubated at 37 °C for 30 minutes. After incubation, 50 µL of kinase-Glo® reagent was added to each well, followed by a 10-minute incubation at room temperature with gentle shaking to stabilize the luminescent signal. Luminescence was then measured using a microplate reader.
The IC_{50} value—defined as the concentration of a compound required to reduce luminescence by 50%, compared to untreated control wells—was determined for each compound from the corresponding dose–response curve generated by plotting PDK-1 inhibition (%) versus compound concentration. Sodium dichloroacetate (SDA, 1000 µM) was used as a reference PDK-1inhibitor.

S3.2.3. In vitro LDHA inhibitory assay.

The LDHA inhibitory activity of the test compounds was investigated using Spectramax M2 spectrofluorometer by measuring the amounts of the consumed NADH [42] employing NAD^+/NADH quantification kit. Briefly, the assay medium containing various concentrations of each test compound (50, 100, 200 and 300 µg/mL) was incubated in assay medium composed of 20 mM of HEPES-K^+ buffer (pH 7.2), 20 µM of NADH, 2 mM of pyruvate and 10 ng of purified recombinant human LDHA protein for 10 minutes. The activity of LDHA would result in reduction of pyruvate into lactate with concomitant oxidation of NADH to NAD^+. Since NADH (reduced form) absorbs at 340 nm whereas NAD^+ (oxidized form) does not, thus, the enzymatic activity of LDHA, could be inversely quantified by monitoring the change in the intensity of the fluorescence at 340 nm over a 10-minute period. The rate of the fluorescence change **(**slope) in treated samples was compared to that obtained from negative control experiment (100 % enzymatic activity) to determine the percentage of inhibition. The IC_{50} value—defined as the compound concentration required to reduce the luminescence by 50%, compared to untreated control wells—was determined for each compound from the corresponding dose–response curve generated by plotting LDHA inhibition (%) against compound concentration. Sodium oxamate (SO, 1000 µM) was used as the reference LDHA inhibitor.

S3.2.4. Cell culture and viability assay.

The cytotoxic activities of the newly synthesized compounds (**3a**-**e**, **8**, **9a**-**c**, **13**, **14**, **17**, **18a**-**h**, **20**, **22**, **23**, and **25**) were examined on human colon cancer cell lines HCT-116 and Lovo (American Type Culture Collection, USA). Various concentrations (50, 100, 200, and 400 µg/mL) of test compounds were prepared in Dulbecco's Modified Eagles Medium (PAN-Biotech, Barcelona, Spain), supplemented with 10 % Fetal Bovine Serum (FBS, Sigma Aldrich; St. Quentin-Fallavier, France), and were then added to cells and incubated for 48 h in a 5 % CO_2-humidified incubator at 37 °C. Afterwards, the supernatant aliquots were collected and the activity of the lactate dehydrogenase—released from the damaged cells [42]—was measured using an ELISA end-point assay (Benchmark Plus, Bio-Rad, CA, USA). The assay medium and 0.1 % Triton X-100 in the assay

medium served used as negative and positive controls, respectively. Percentages of viable cells were calculated based on relative optical density (OD) values (at 550 nm) for compound-treated wells (final concentration of 100 μg/mL), compared to the negative control, and expressed as mean value (%) ± SD from two replicates. Graphing dose-response curves representing the compound concentration versus the percentages of viable cells was also conducted to deduce the half-maximal concentration providing 50% growth inhibition (IC_{50}) for the promising candidates. Additionally, the safety profiles of the studied compounds were assessed against normal cells (HUVECs) at final concentration of 100 μg/mL. HUVECs were grown in Dulbecco's Modified Eagle's Medium supplemented with 10% FBS, 2 mM glutamine (Sigma Aldrich), 100 units/mL penicillin and 100 mg/mL streptomycin (Life Technologies; Paisley, UK).

S3.2.5. Cell cycle assay.

Flow cytometric analysis using Propidium Iodide Flow Cytometry Kit for Cell Cycle Analysis (Abcam-UK), was used in order to analyze the changes in cell cycle distribution induced in HCT-116 and LoVo cultures upon treatment with compound **3d**. HCT-116 and LoVo carcinoma cells (5×10^4 cells/mL) were treated with DMSO as a negative control, and **3d** compound at 30 μg/mL for 48 h. The pellets remained after centrifugation were washed twice with PBS. Then, cells were fixed by mixing 700 mL of 90% cold ethanol and stained with propidium iodide (PI) for 1 h at 37 °C. RNase A (10 mg/mL) was added in order to limit the ability of PI to bind only to DNA molecules. Then, the stained cells were analyzed for DNA content by BD FACSC flow cytometer.

S3.2.6. Annexin-V-FITC assay.

Annexin V-FITC Apoptosis Detection Kit (BioVision Research Products, Mountain View, CA, USA) was used to analyze the distribution of early and late apoptotic cells, as well as necrotic cells, after treatment with compound **3d**. HCT-116 and LoVo cells were treated with vehicle (DMSO) and **3d** at 30 μg/mL for 48 h. After treatment, cells were harvested and, washed with PBS, and resuspended in Annexin-V binding buffer (BioVision Research Products, USA). The cells were then stained with Annexin V-FITC and propidium iodide (PI). Using the FITC signal detector (usually FL1) for Annexin V and the PI staining detected through by the phycoerythrin emission signal detector (through quadrant statistics for necrotic and apoptotic cell populations) the fluorescent intensities of stained cancerous cells were determined.

S3.2.7. Analysis of reactive oxygen species (ROS) levels.

Human ROS ELISA kit was used in order to measure the intracellular accumulation of ROS in the **3d-**treated LoVo and HCT-116 cells. 100 μl of Anti-Human ROS specific antibody has been pre-coated onto 96-well plate. Human ROS present in the standards/homogenates treated cells with 30 μg/mL of compound **3d** for 48 h, bind to the capture antibody (Anti-Human ROS specific antibody). Subsequently, 100 μl of biotinylated anti-human ROS detection antibody was added to form an Ab-Ag-Ab sandwich and incubated for 1 h at 37 °C. After a washing step, 100 μl of streptavidin-HRP was added for 30 min at 37 °C. The unbound conjugate was removed with wash buffer. Then, 90 μL of HRP substrate, tetramethylbenzidine (TMB) was added followed by incubation in the dark for 15–30 minutes at 37 °C. HRP substrate, TMB, results in the production of a blue colored product that changes to yellow after the addition of 50 μL of acidic Stop Solution. The density of yellow color was quantified by measuring the absorbance at 540 nm, which is directly proportional to the amount of Human ROS captured on the plate.

S3.2.8. Mitochondrial transmembrane potential (MMP) measurement.

The change in mitochondrial transmembrane potential ($\Delta\Psi_m$) of HCT-116 and LoVo colon carcinoma cells treated with compound **3d** was assessed using the TMRE Mitochondrial Membrane

Potential Assay kit (ab113852, Abcam, UK). Cells were incubated with 30 μg/mL of compound **3d** for 48 h. Carbonyl cyanide 4-(trifluoromethoxy)phenylhydrazone (FCCP), an uncoupler that eliminates the mitochondrial membrane potential and prevents staining by TMRE (tetramethylrhodamine, ethyl ester), was used as positive control. After that, TMRE was added to the cells in media and incubated for 15–30 minutes at 37 °C, followed by centrifugation and resuspension of pellet cells in PBS/0.2% BSA buffer. Live cells were examined in the flow cytometer for TMRE staining. TMRE was excited using a 488 nm laser and detected in the FL2 channel at 575 nm.

S3.2.9. Quantification of the Expression Levels of *Bax*, *Bcl-2* and *Caspase-3* Genes

- Cell culture

  Human colon cancer cell lines (HCT-116 and LoVo) were cultured for 24 h in a 25 mL flask at a density of 1 * 10^6 cells/flask as described in section S3.2.4. Cells were treated with 30 μg/mL of compound **3d** in 2 mL of fresh medium containing 1% FBS, or with 0.25 % DMSO as a negative control, for 48 h. Afterwards, cells were washed with ice-cold phosphate-buffered saline (PBS) and adherent cells were detached using trypsin solution.

- Primers used

  Table S7 lists primers amplifying 100-200 bp of *Bax*, *Bcl-2* and *Caspase-3* genes. These primers were designed using primer blast software (https://www.ncbi.nlm.nih.gov/tools/primer-blast/, accessed on 9-01-2023).

Table S7. Primer sequences for *Bax, Bcl-2*, *Caspase-3*, and *GAPDH* (control)

**Gene**	**Primer sequence (5′ to 3′)**
Bax	F 5′- TCAGGATGCGTCCACCAAGAAG-3′,<br>R 5'- TGTGTCCACGGCGGCAATCATC-3'.
Bcl-2	F 5′-ATCGCCCTGTGGATGACTGAGT -3′,<br>R 5'- GCCAGGAGAAATCAAACAGAGGC-3'.
Caspase 3	F 5′- GGAAGCGAATCAATGGACTCTGG-3′,<br>R 5'-GCATCGACATCTGTACCAGACC -3'.
GAPDH	F 5′- GTCTCCTCTGACTTCAACAGCG-3′<br>R 5′- ACCACCCTGTTGCTGTAGCCAA-3′

- Reverse-Transcription PCR (RT-PCR)

  The total RNA was extracted from untreated and treated cells using the RNeasy Mini Kit (Qiagen, DE). A Nanodrop 8000 spectrophotometer (Thermo Fisher Scientific, USA) was used to check the RNA purity and quality. The Hyperscript Kit (GeneAll, KR) and random hexamers (GeneAll, KR) were used as described in the manufacturer's protocol to reverse-transcript 1μg of RNA from each sample into cDNA.

- Quantitative real-time PCR

  The mRNA expression of *Bax*, *Bcl-2*, and *Caspase-3* were measured using QuantStudio 7Flex Detection System (Applied Biosystems, USA). The iScript One-Step RT-PCR Kit with SYBR Green (Cat no: 170-8892; Bio-Rad, CA, USA) was used to carry out the reactions for the synthesis of cDNA. Each cDNA sample (5 μl) was added in a 20 μL PCR mixture

containing the corresponding primer (Table S7) at 10 pM, 12.5 μL of 2 X iScript One-Step RT-PCR Kit with SYBR Green, and 7 μL RNase/DNase-free water (Qiagen, DE). The thermal cycling conditions for *Bax*, Bcl-2, and *Caspase-3* genes were established as 5 min at 95 °C, followed by 40 cycles of 30s at 95 °C and 30s at 60°C, and final 10s at 95 °C. The presence of a single melting temperature peak verified the specificity of each primer. The expression of the housekeeping gene *GAPDH*, was used as an endogenous control.

^{1}H-NMR and ^{13}C-NMR spectra of some selected Examples

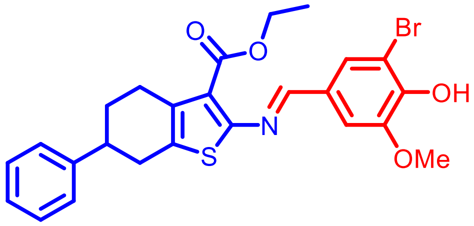

Compound 3a (DMSO-d_6)

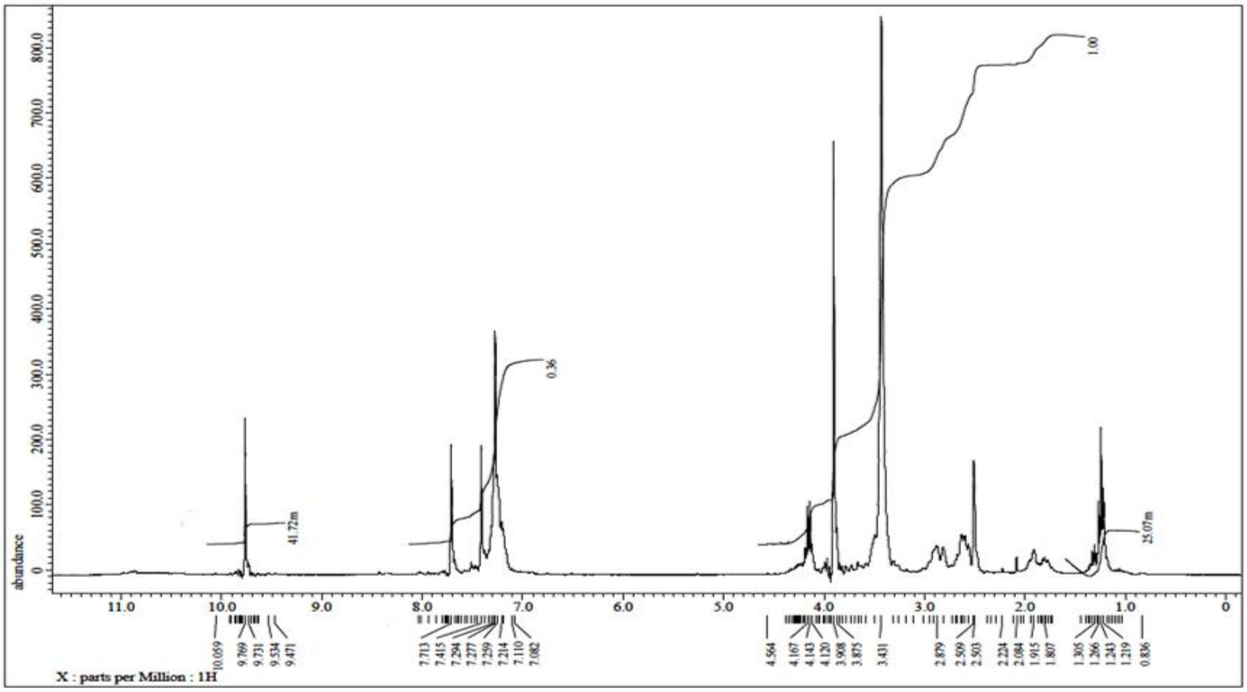

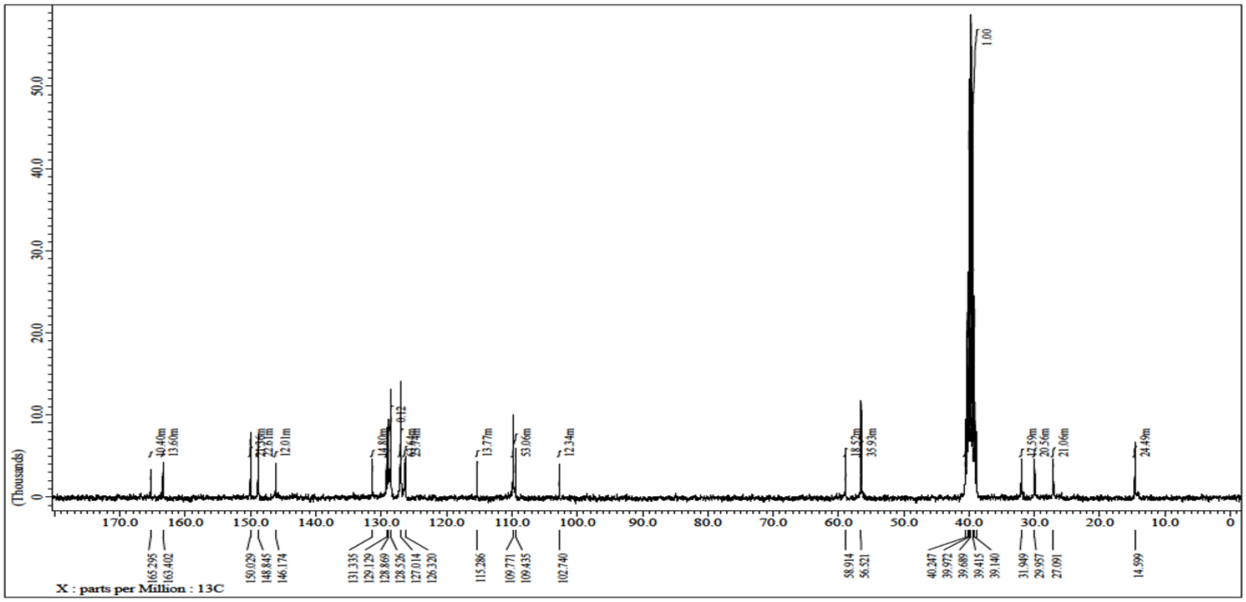

Compound 3b (DMSO-d_6)

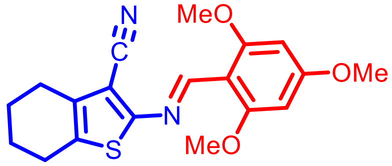

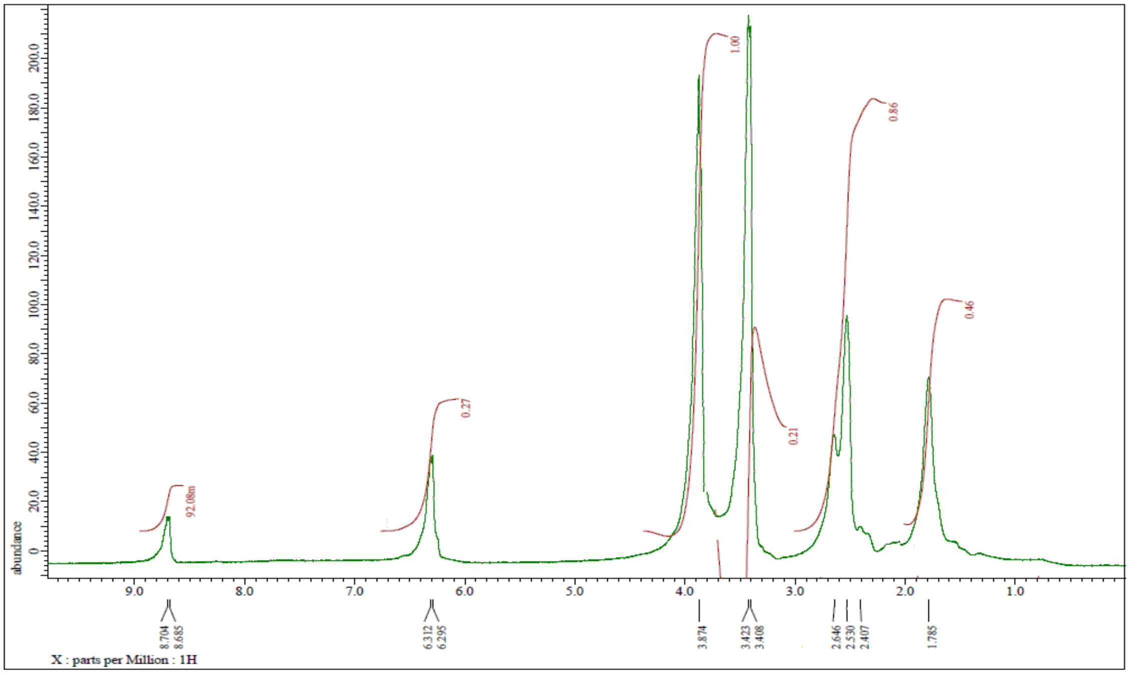

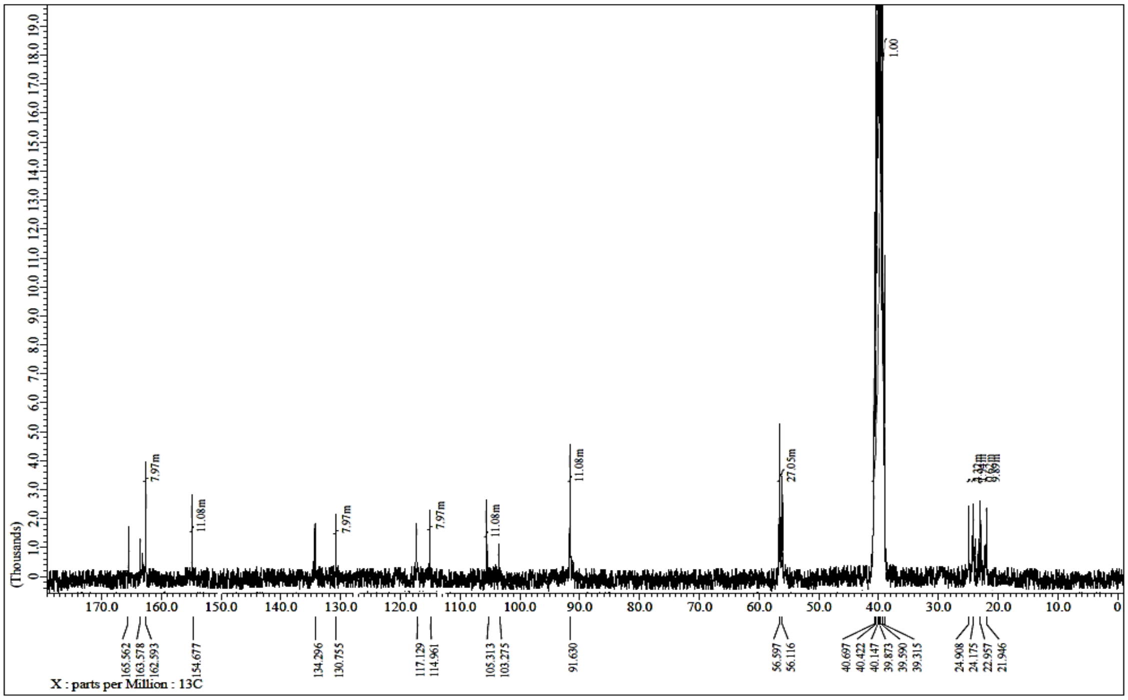

Compound 3c (DMSO-d_6)

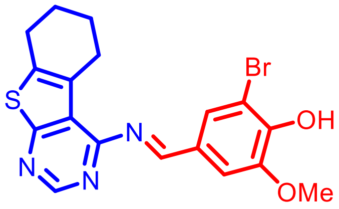

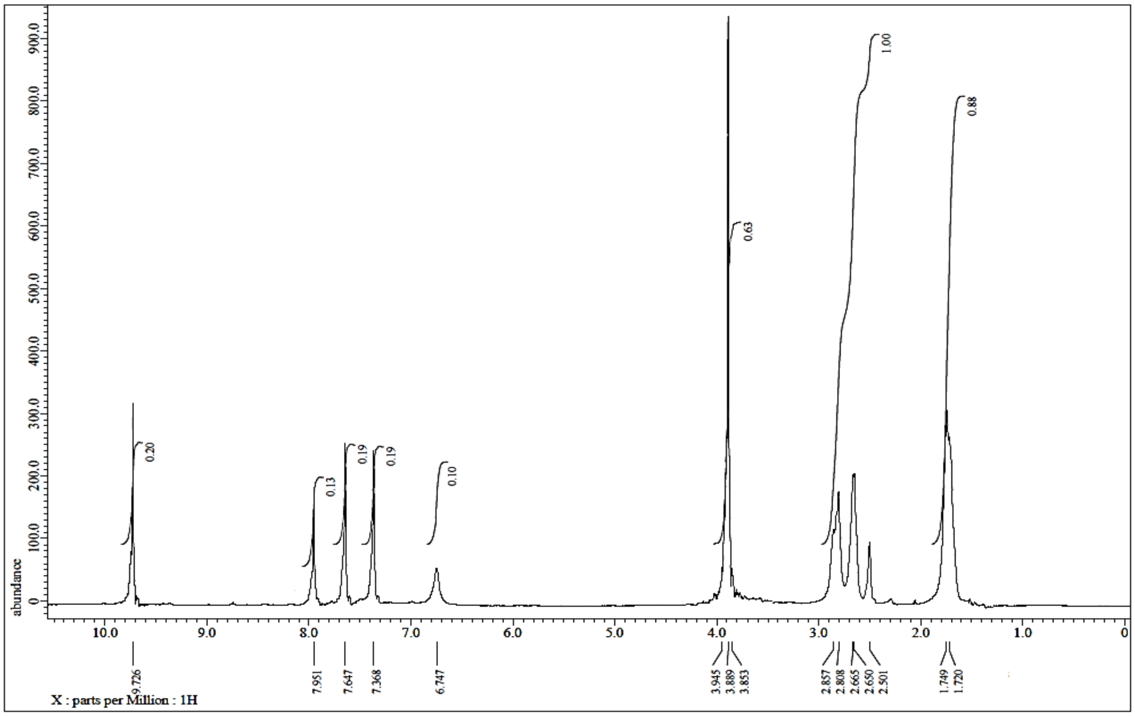

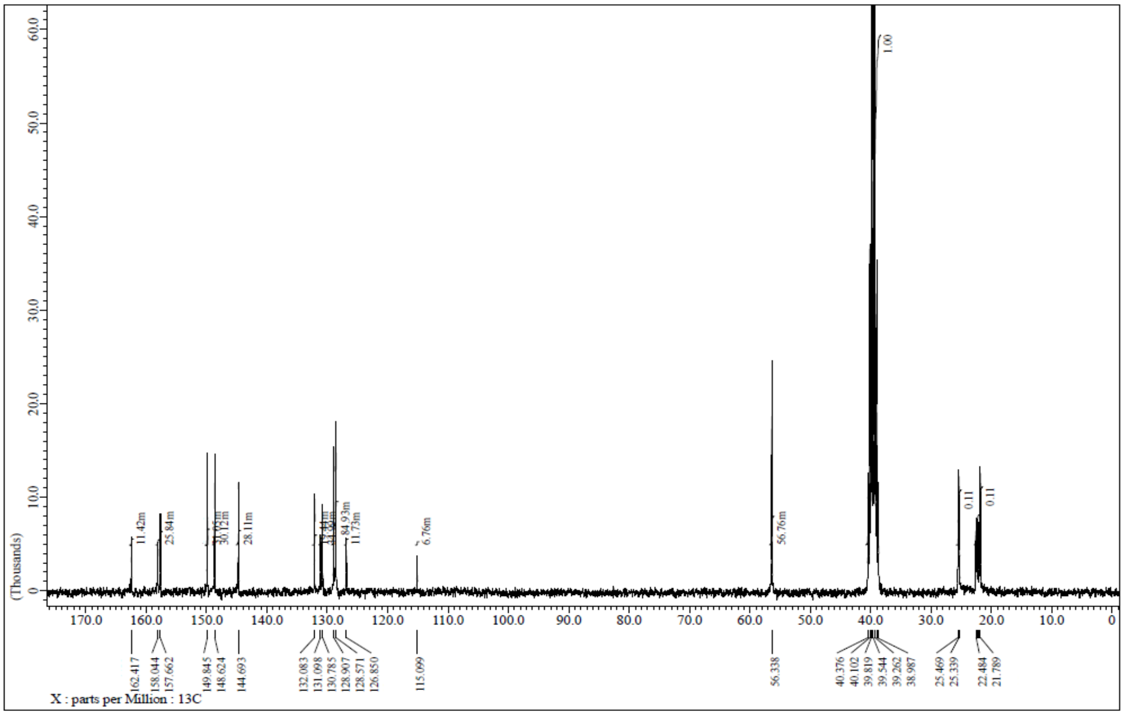

Compound 3d (DMSO-*d*6)

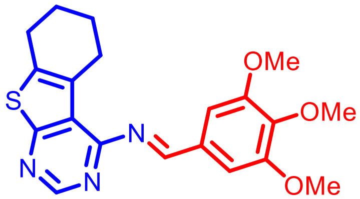

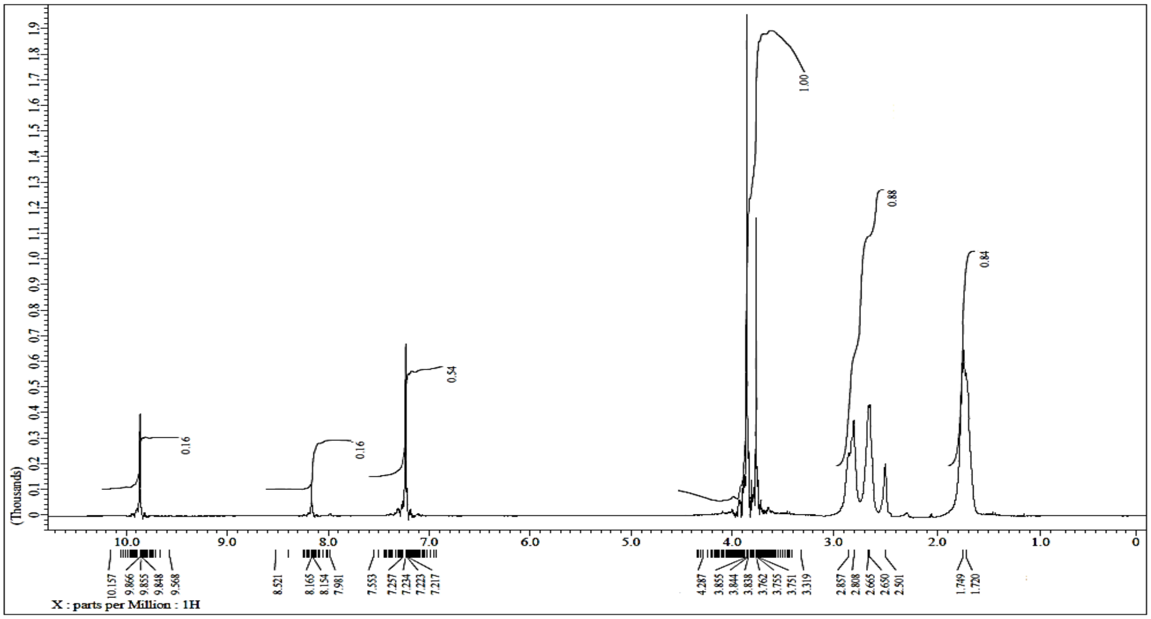

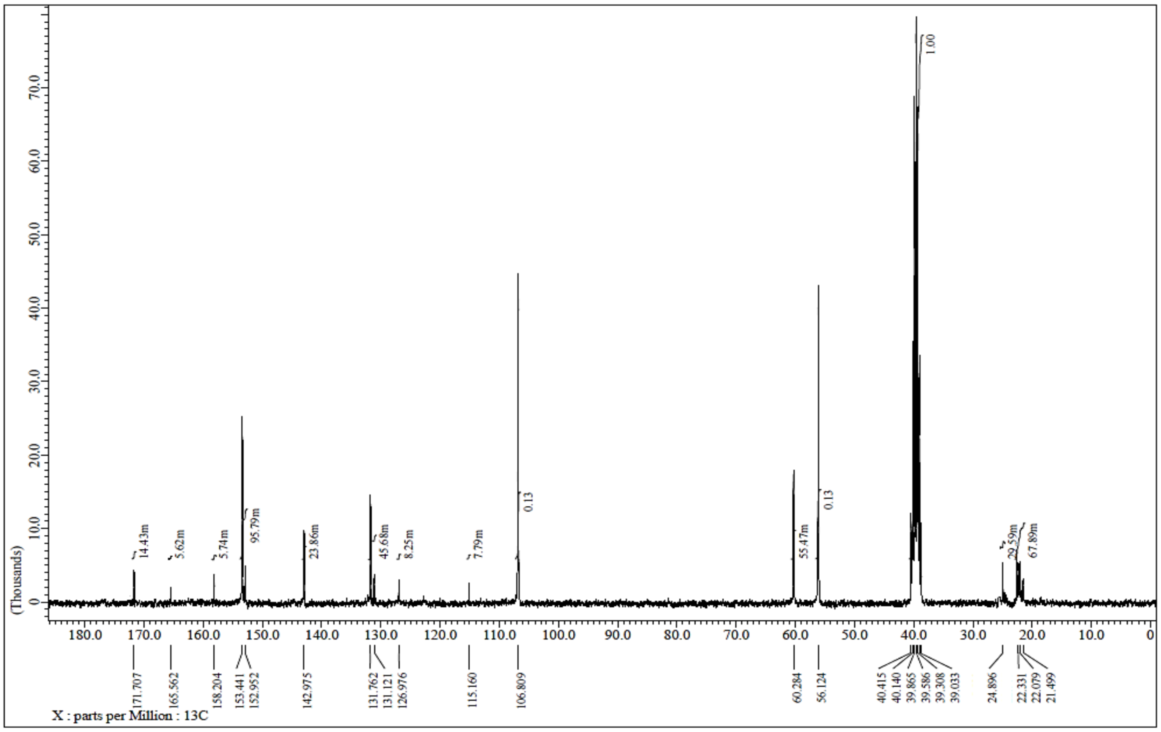

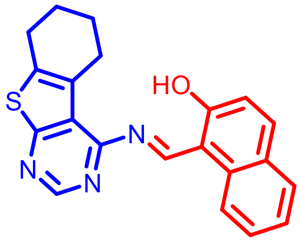

Compound 3e (DMSO-*d*$_6$)

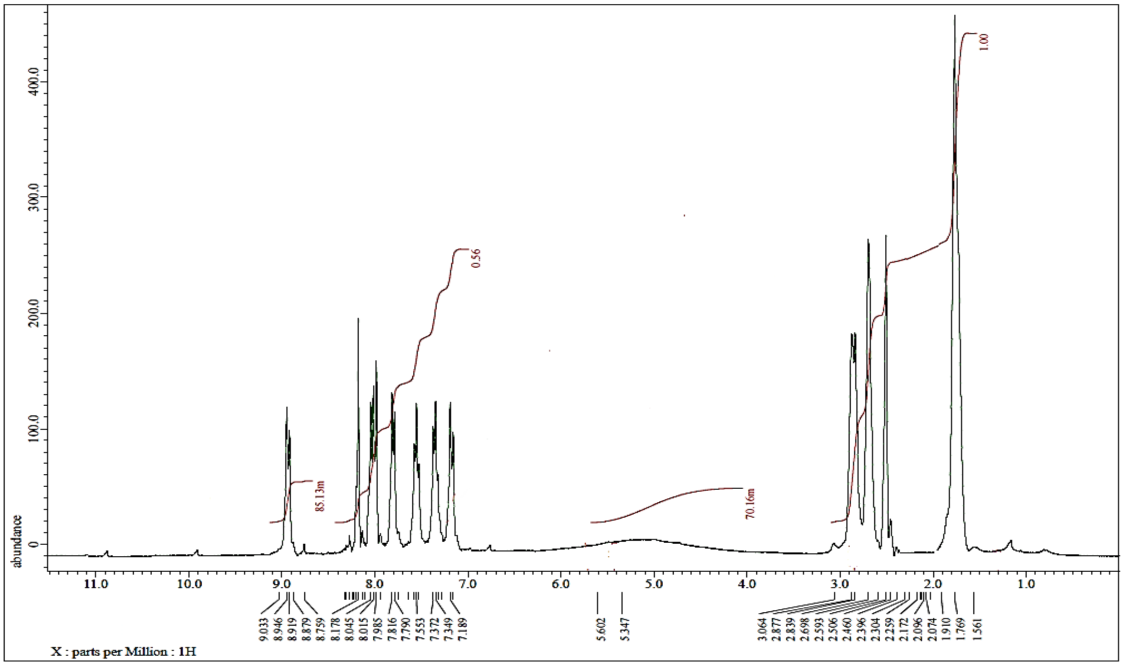

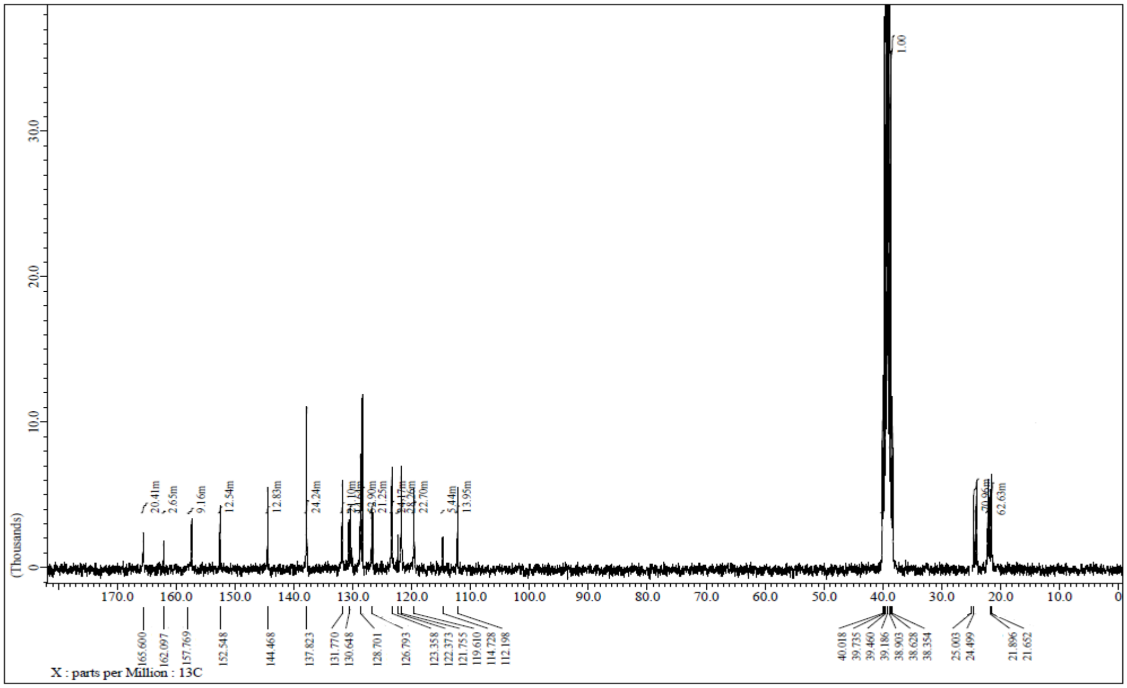

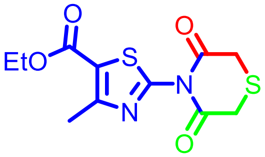

Compound 8 (DMSO-d_6)

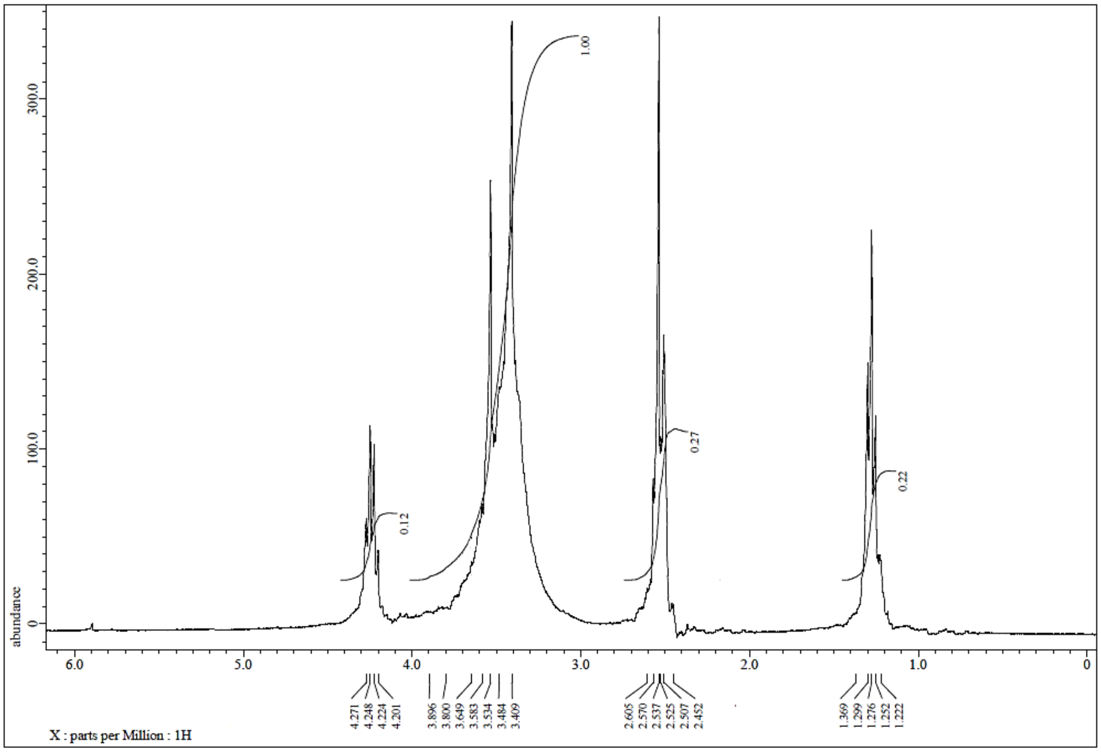

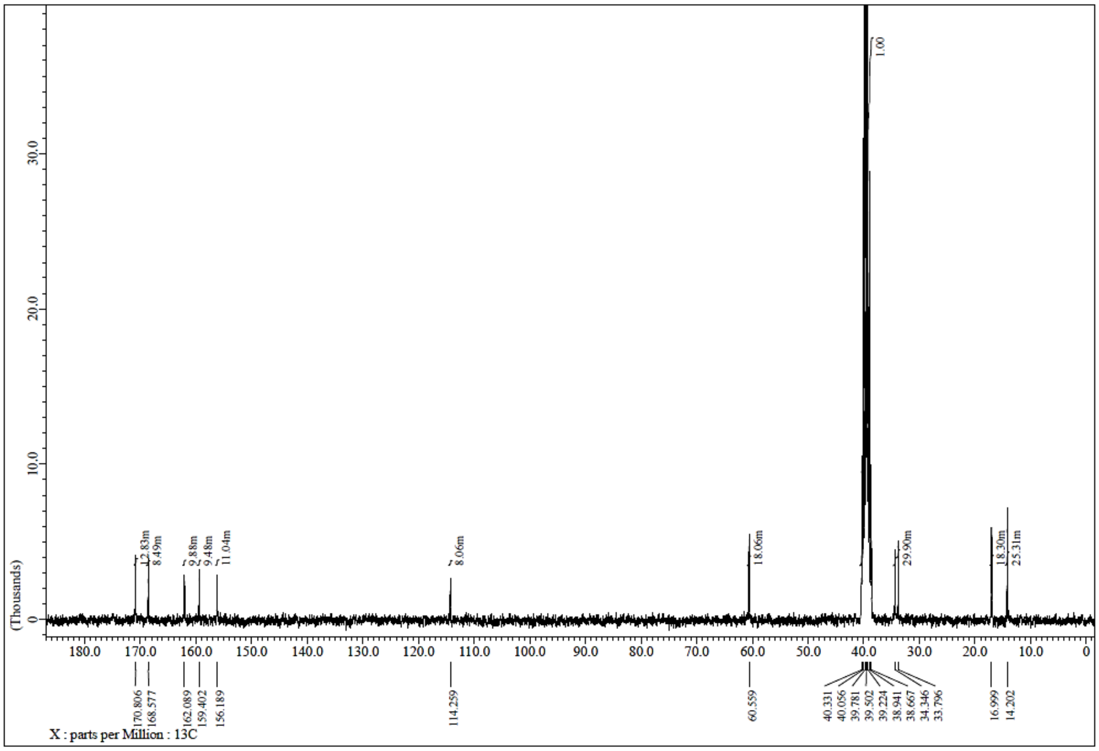

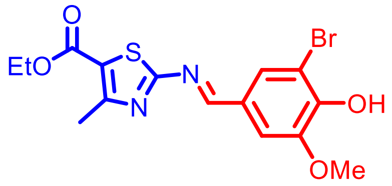

Compound 9a (DMSO-d_6)

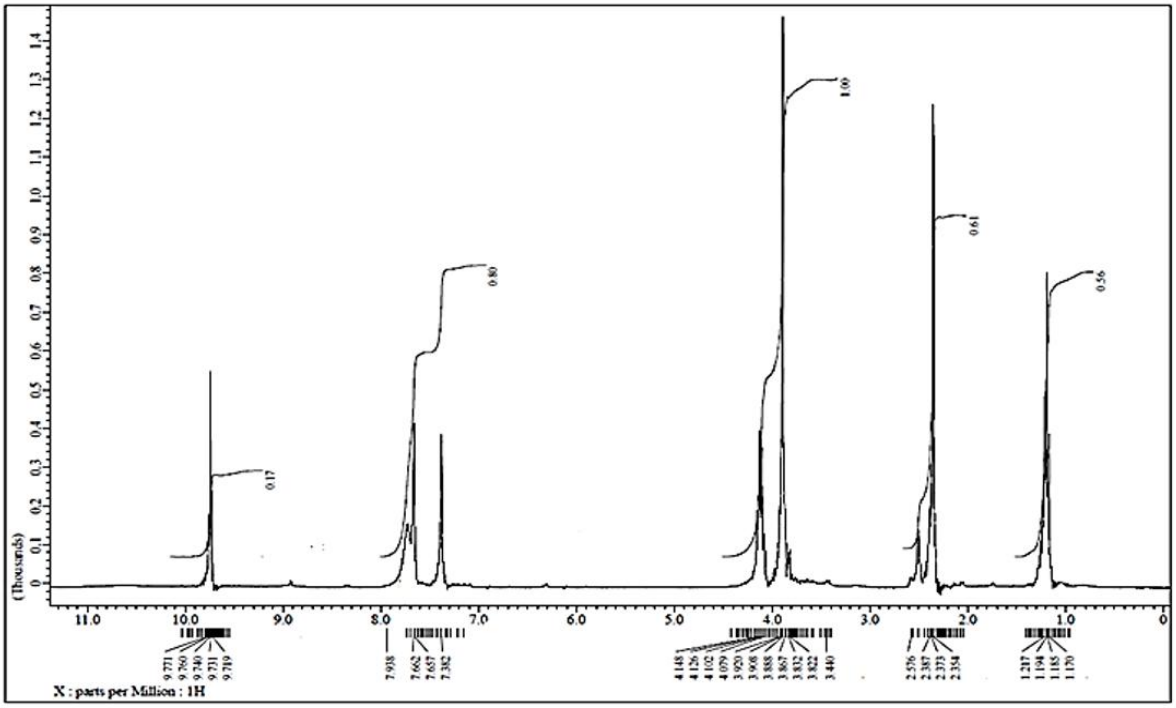

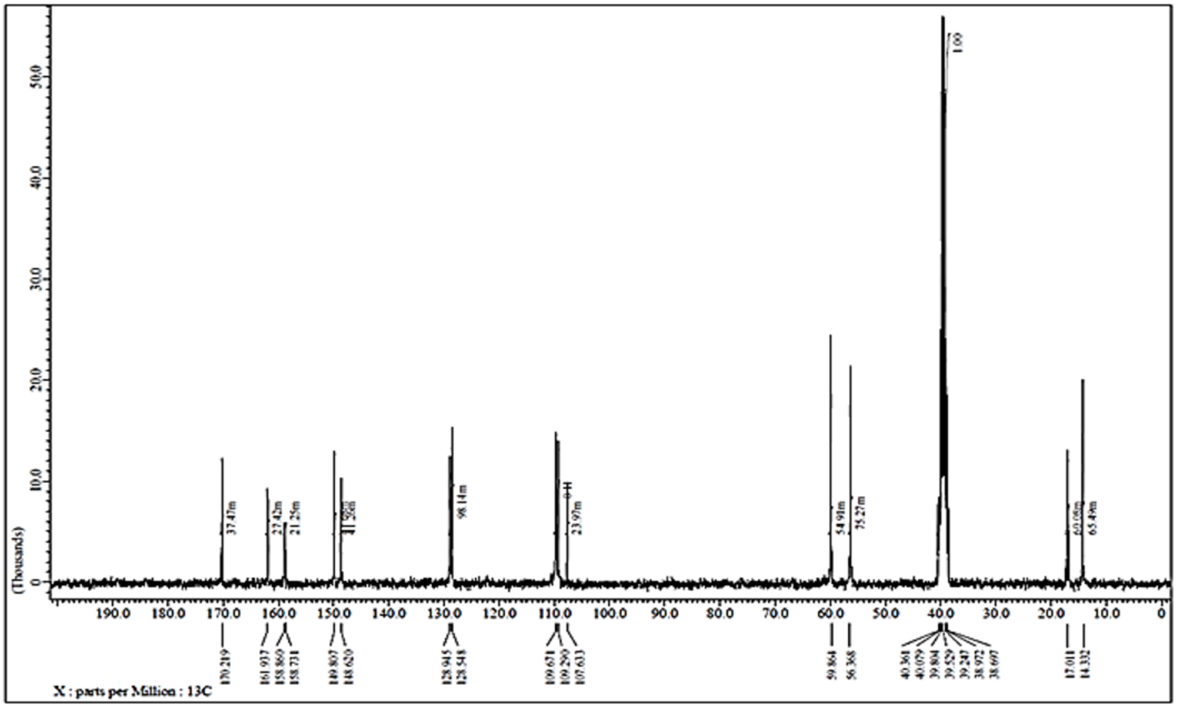

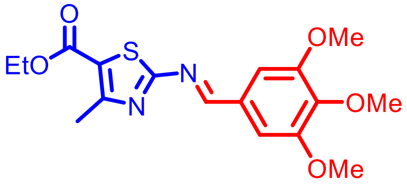

Compound 9b (DMSO-d_6)

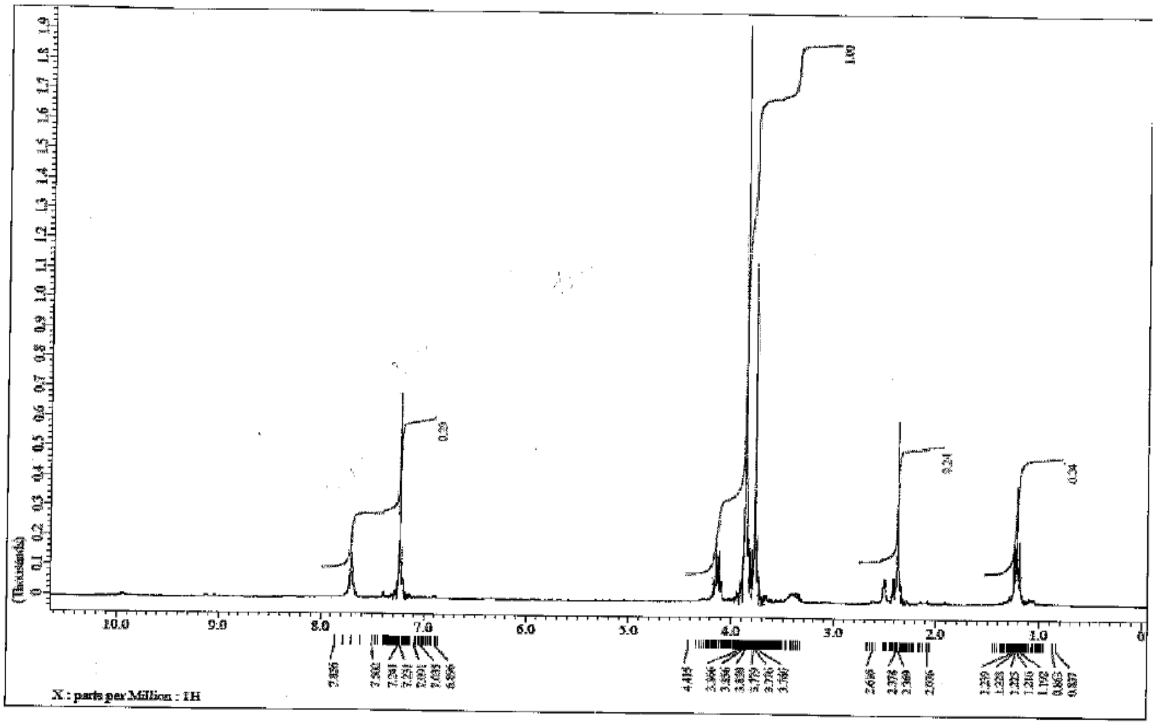

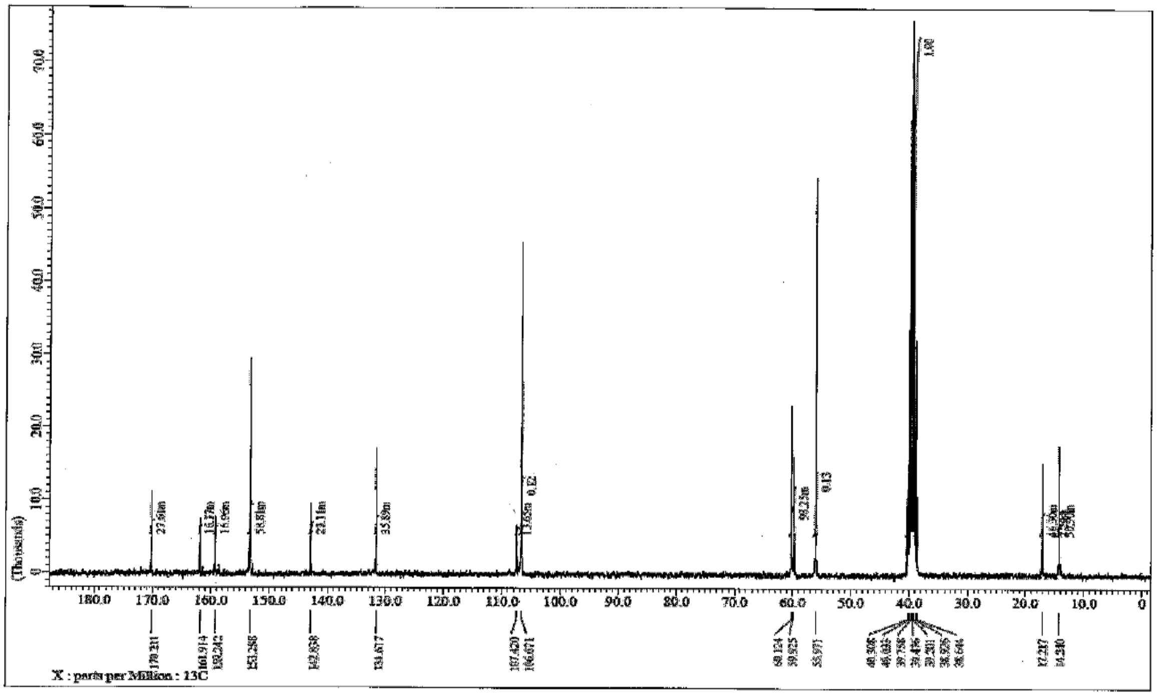

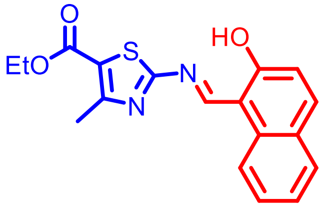

Compound 9c (DMSO-d_6)

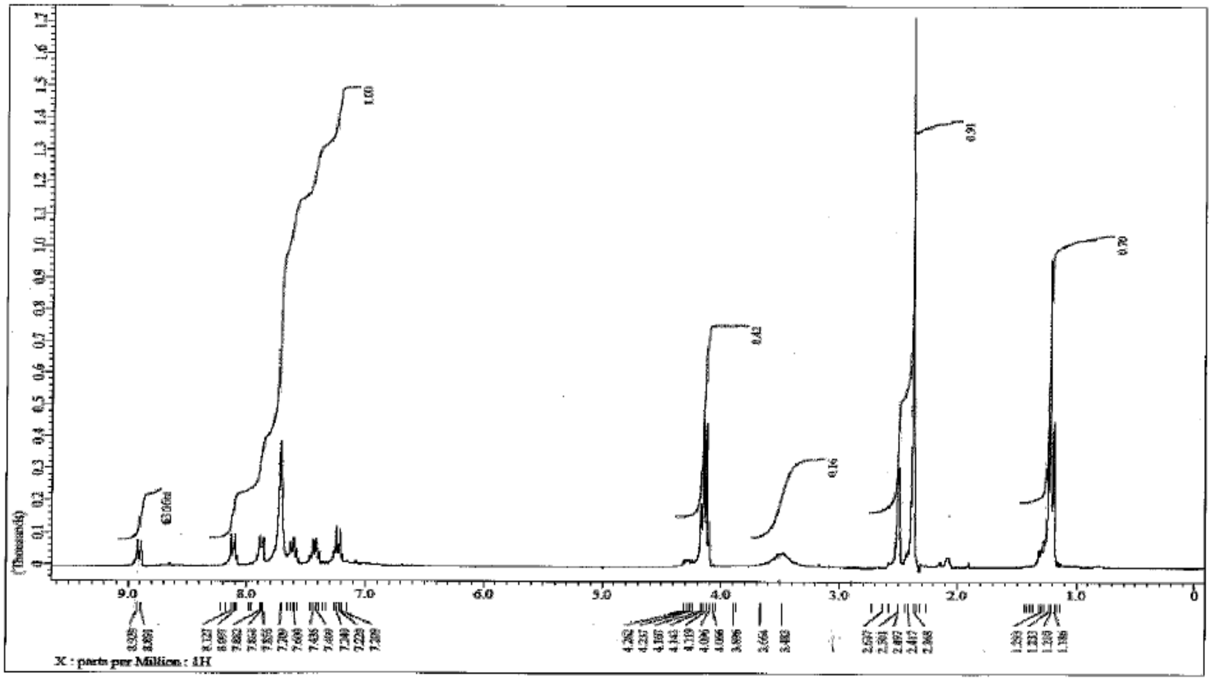

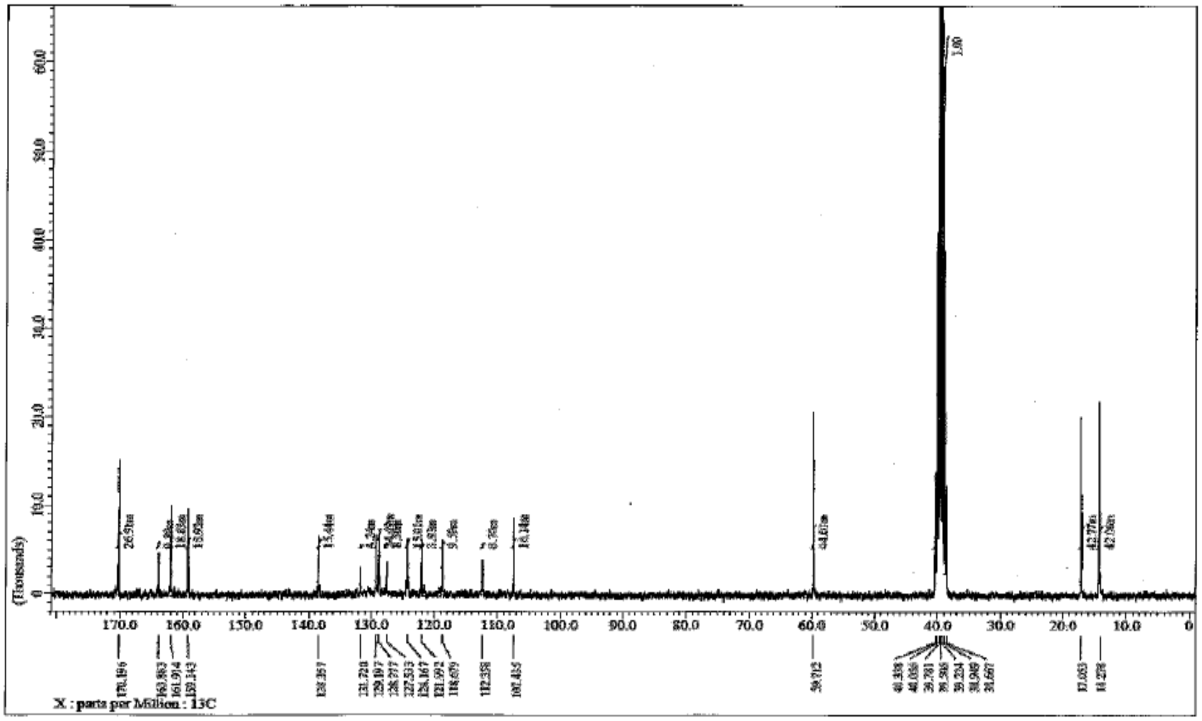

Compound 14 (DMSO-d_6)

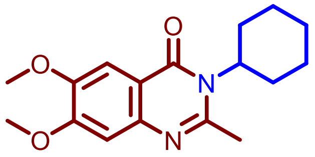

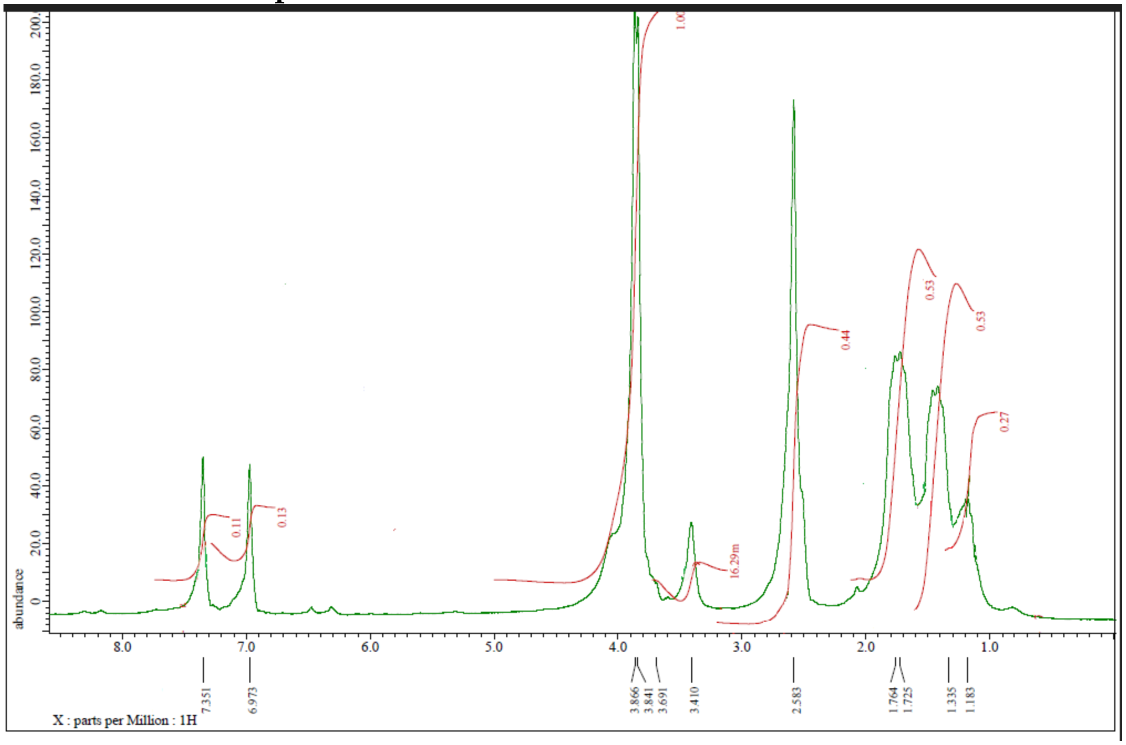

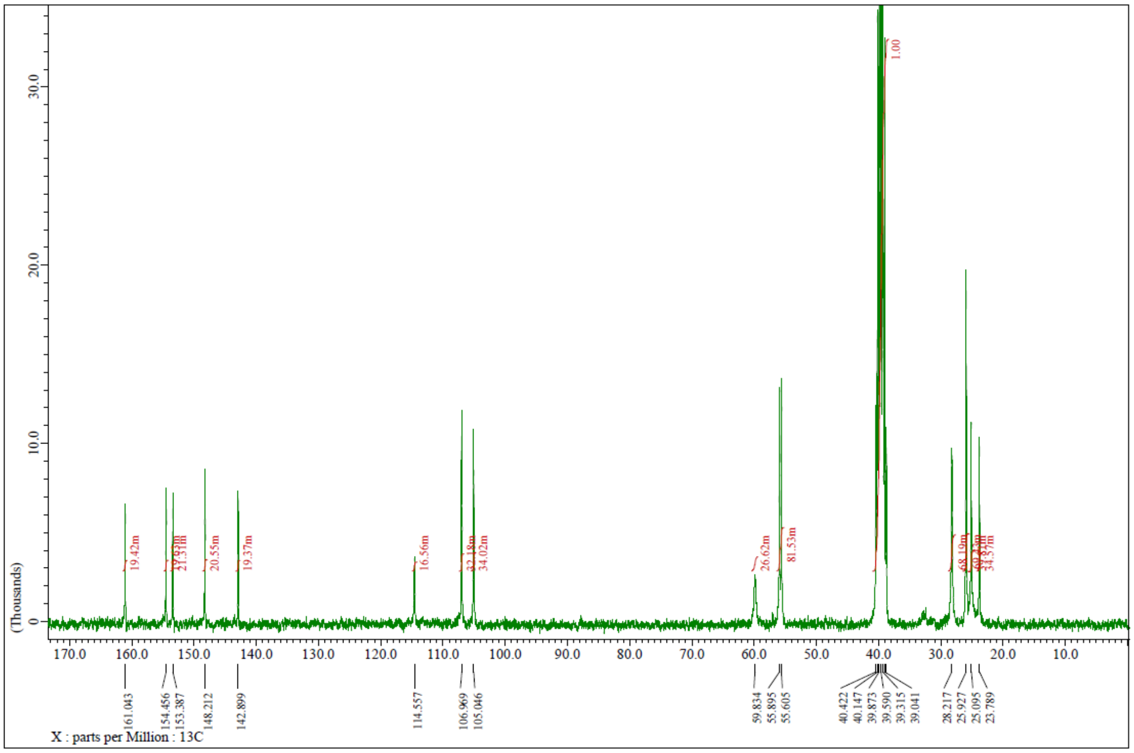

Compound 17 (DMSO-*d*6)

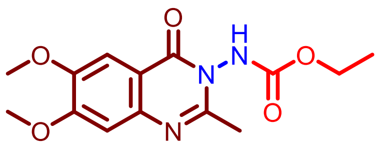

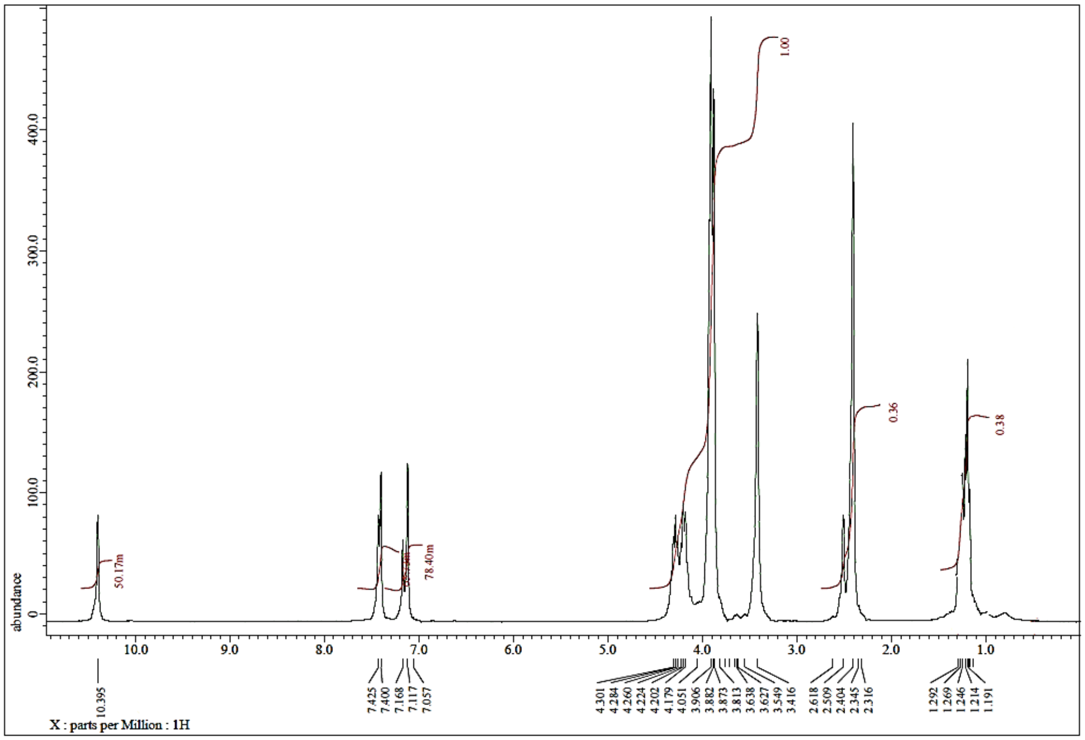

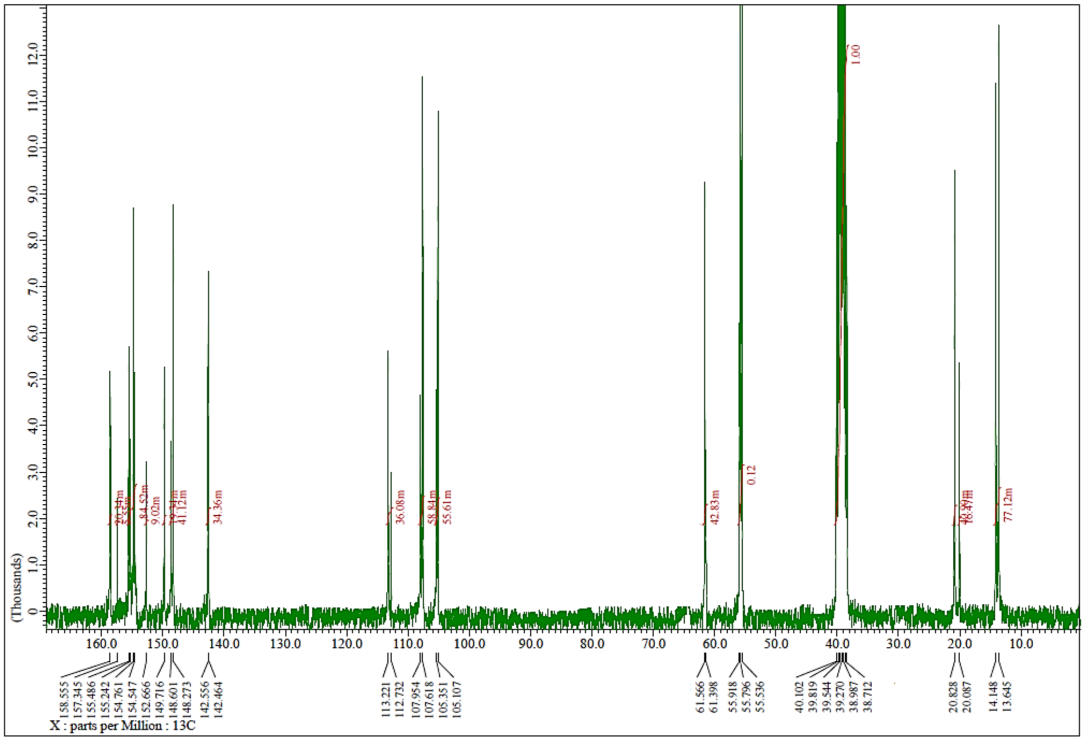

Compound 18b (DMSO-d_6)

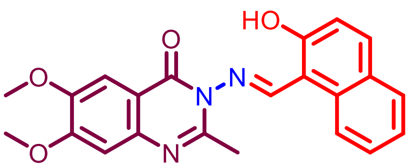

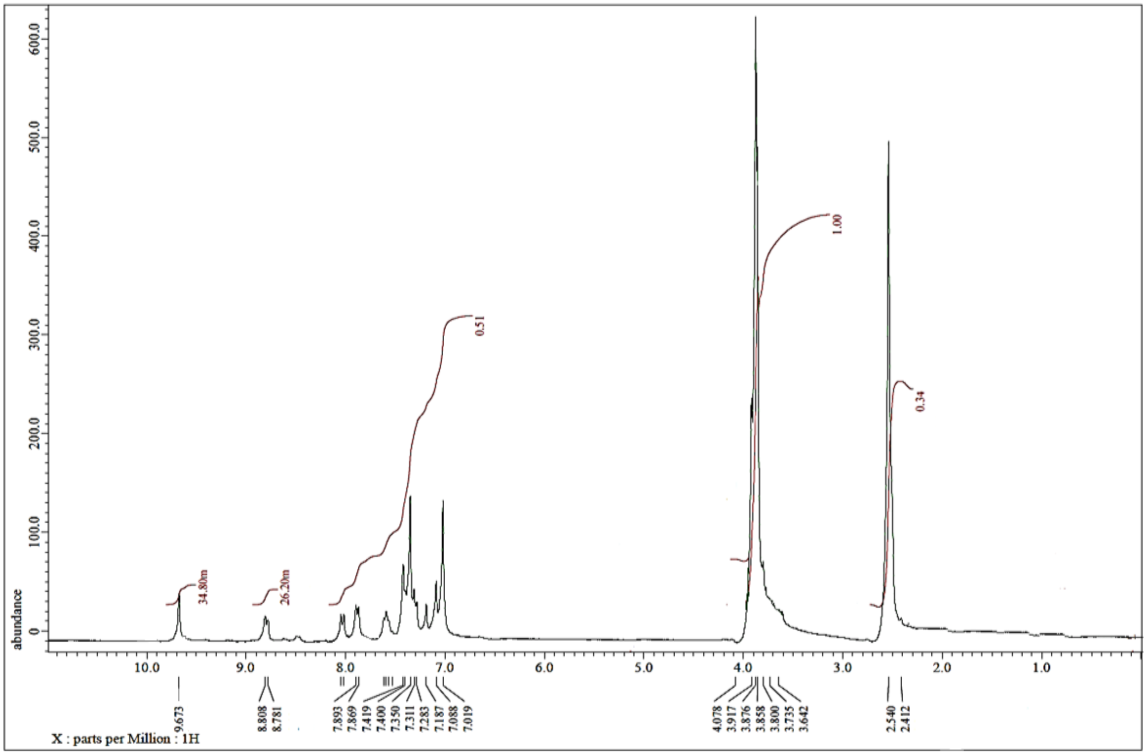

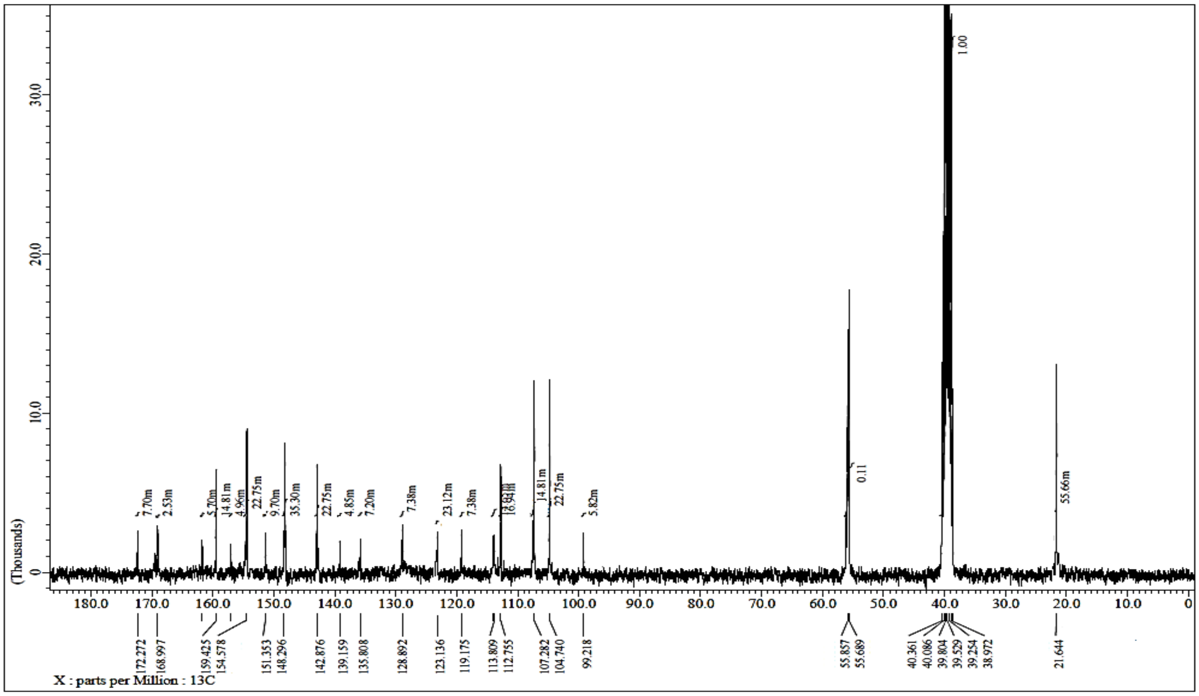

Compound 18c ($CDCl_3$)

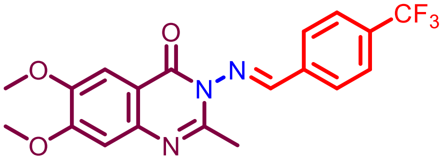

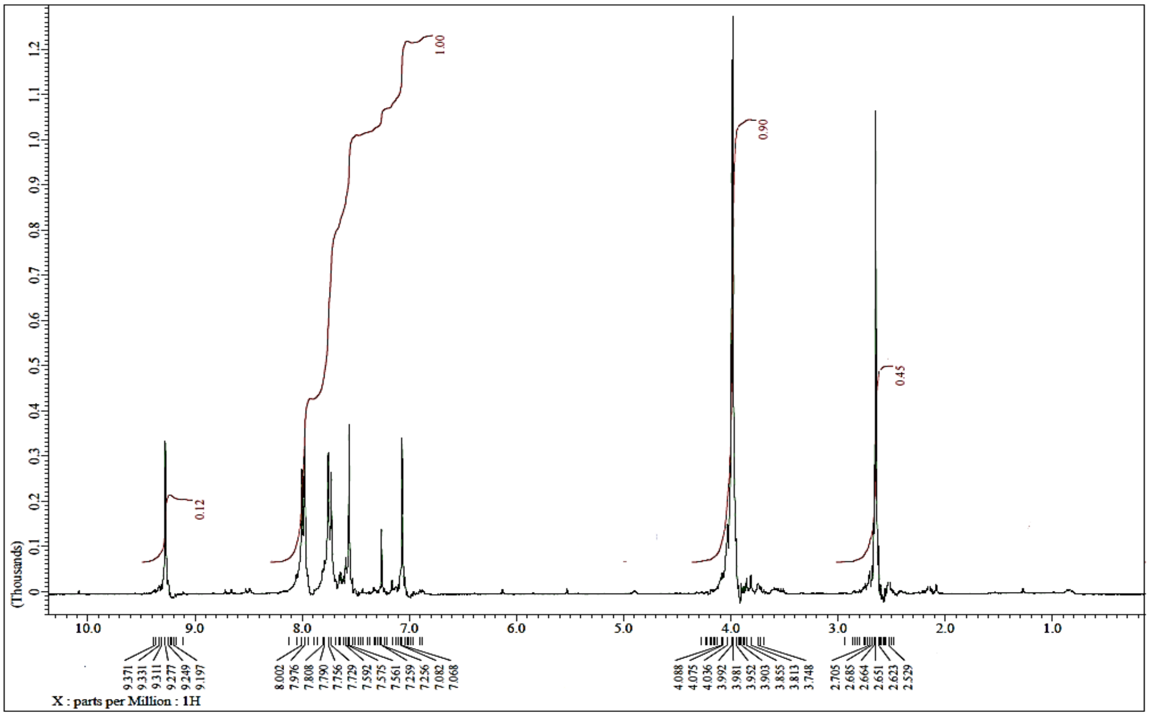

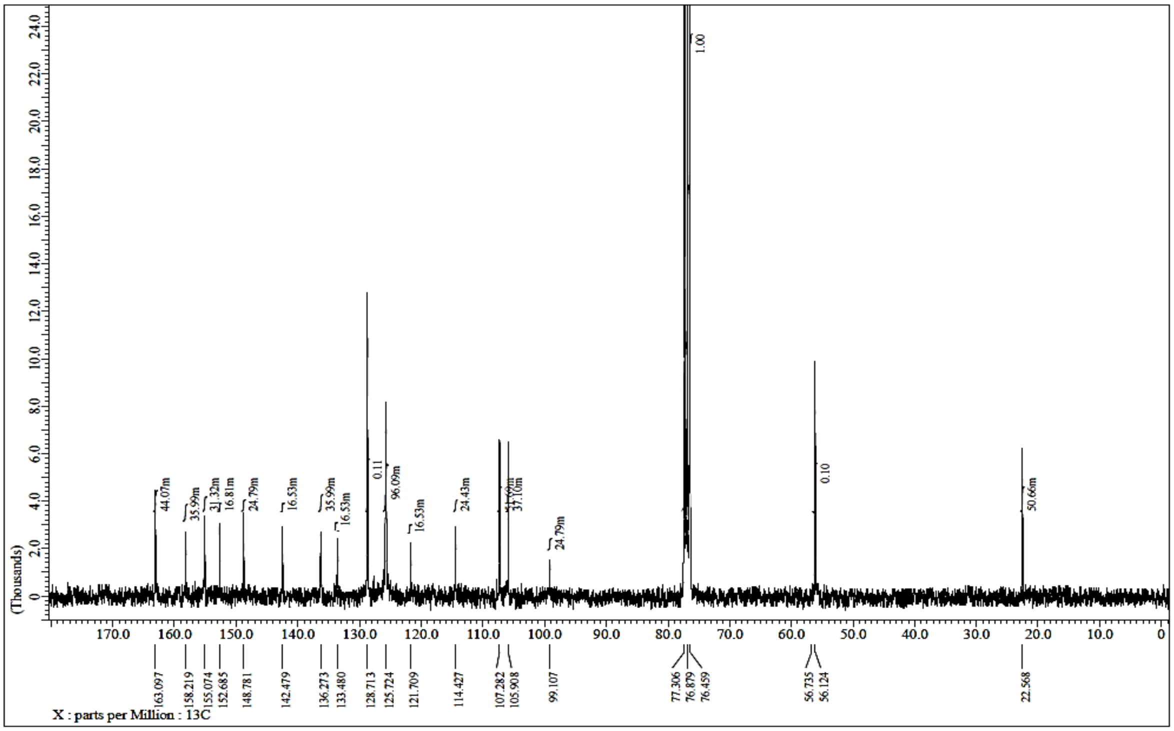

Compound 18d (DMSO-d_6)

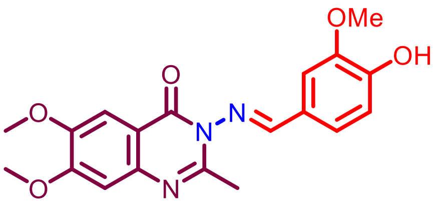

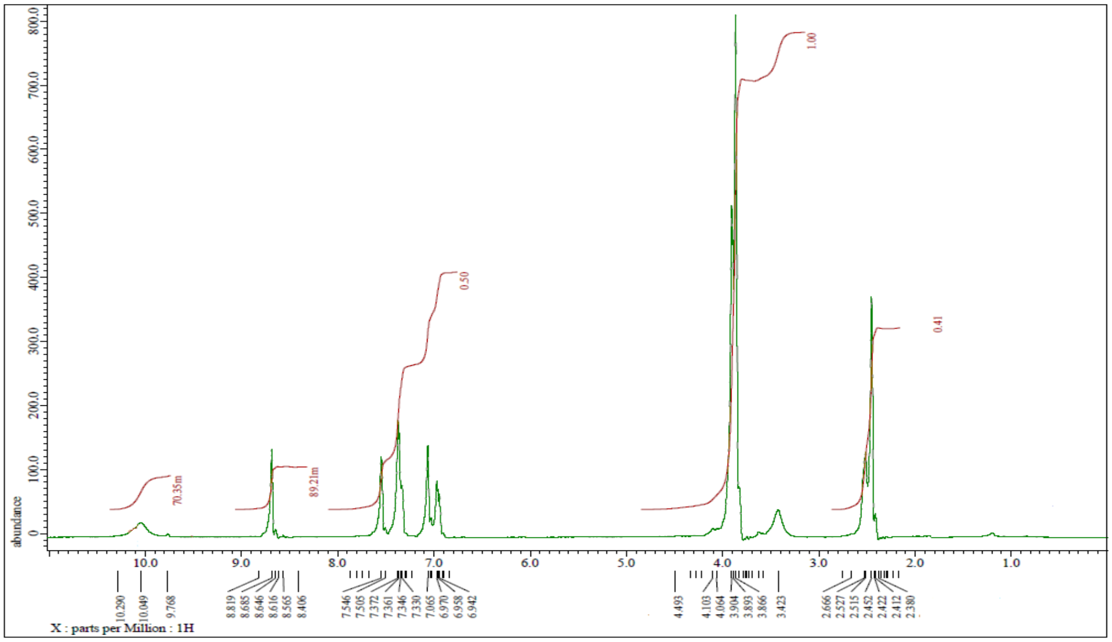

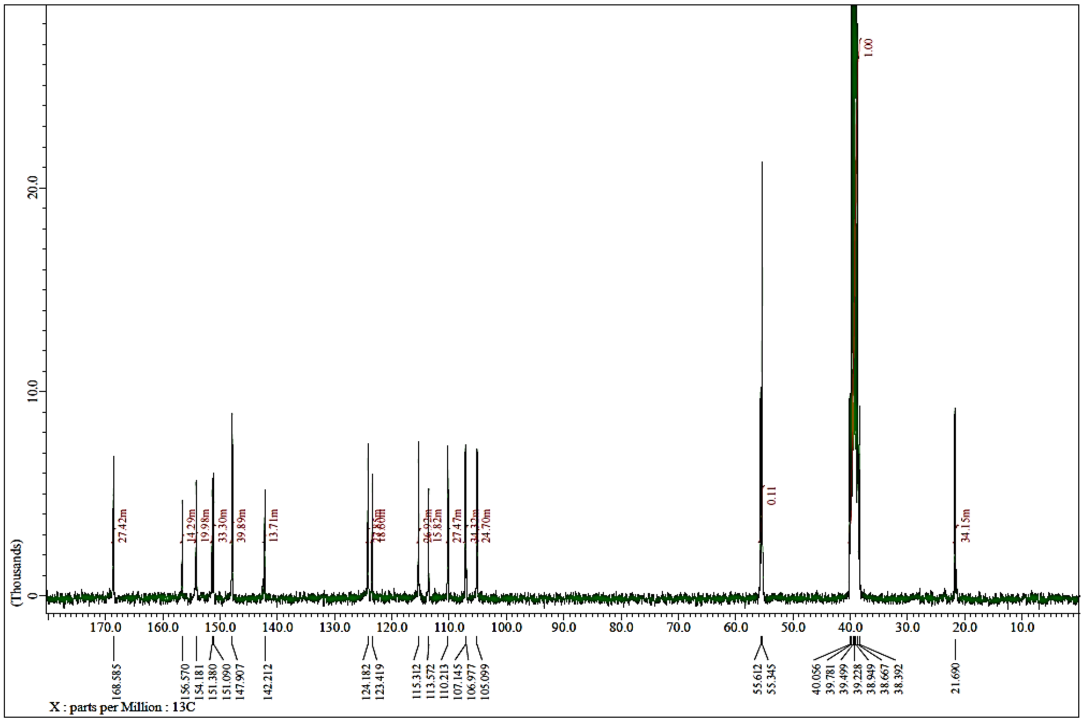

Compound 18e

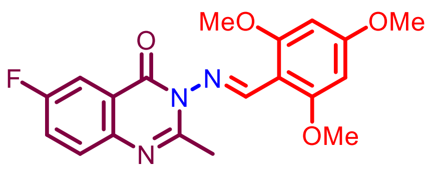

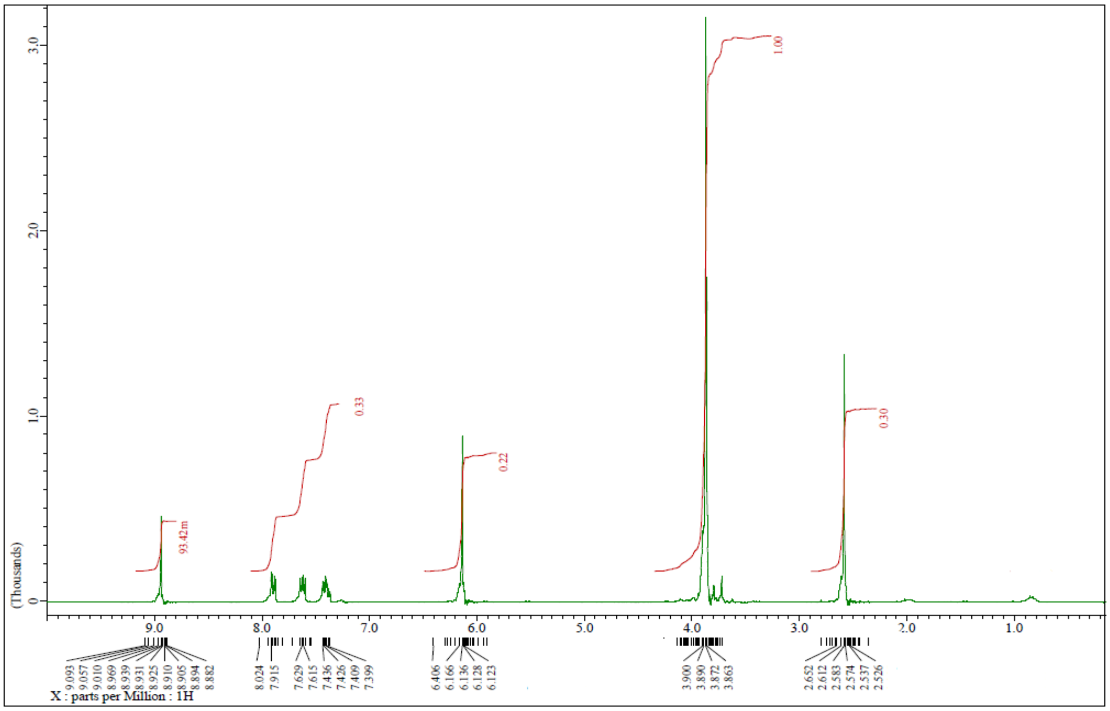

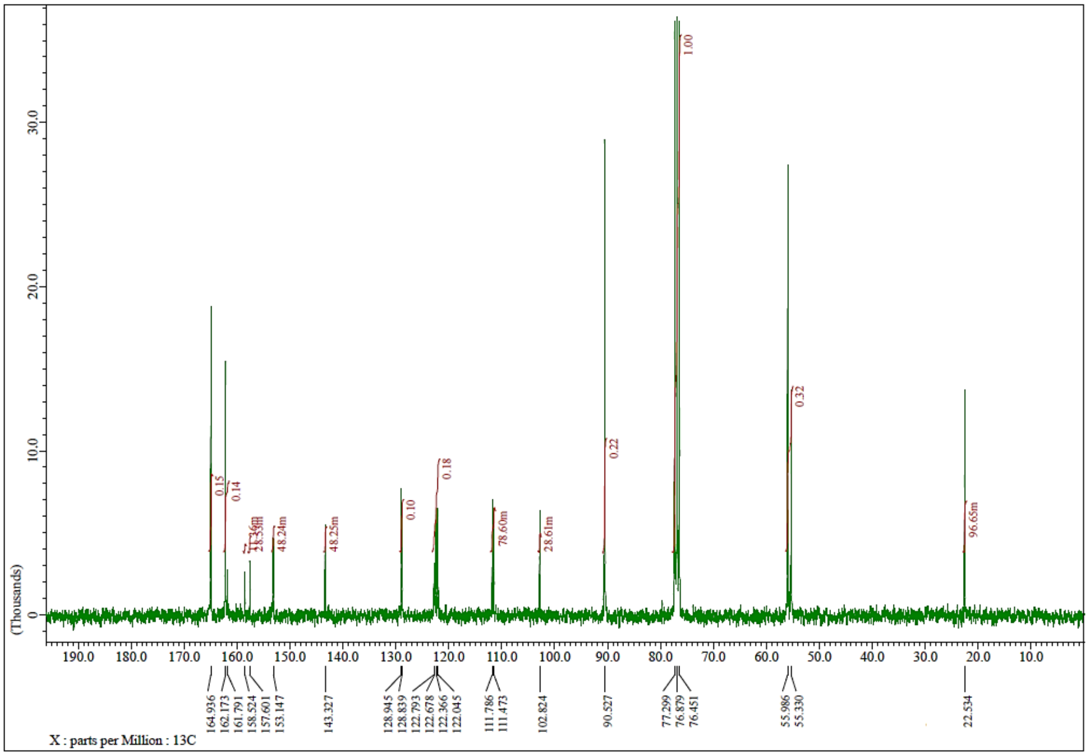

Compound 18f (DMSO-d_6)

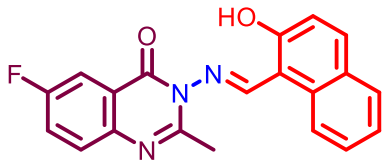

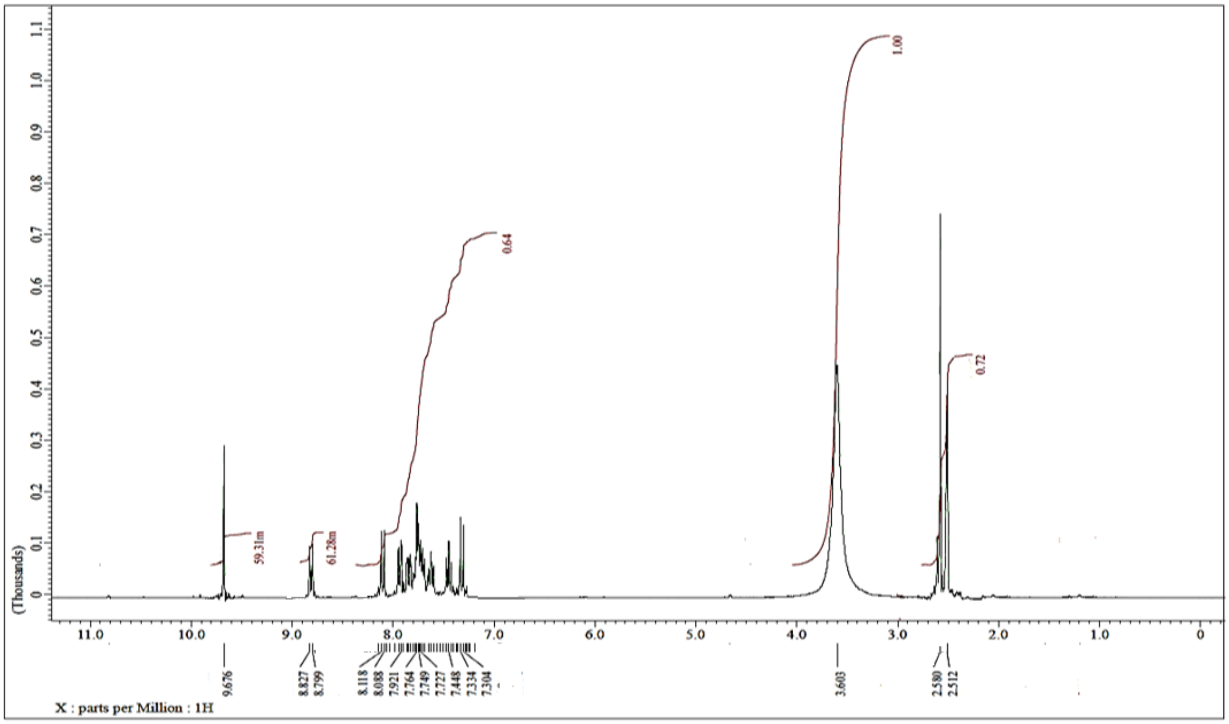

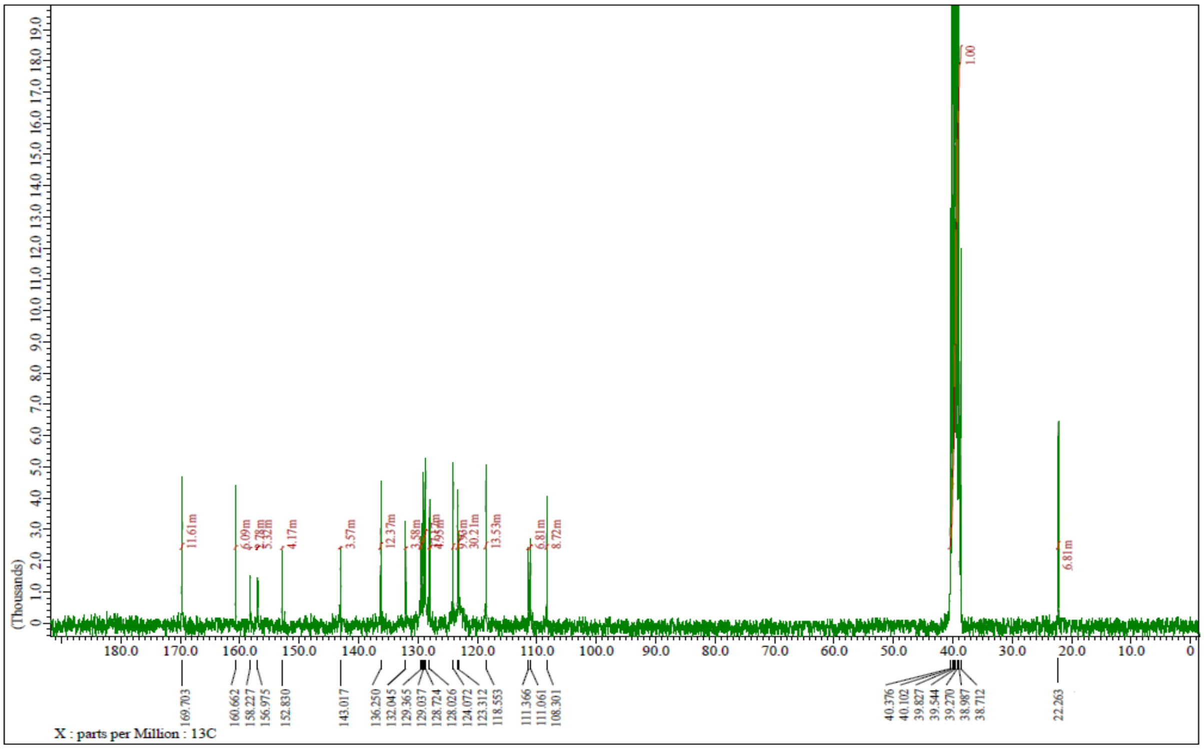

Compound 18h (DMSO-d_6)

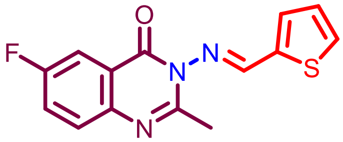

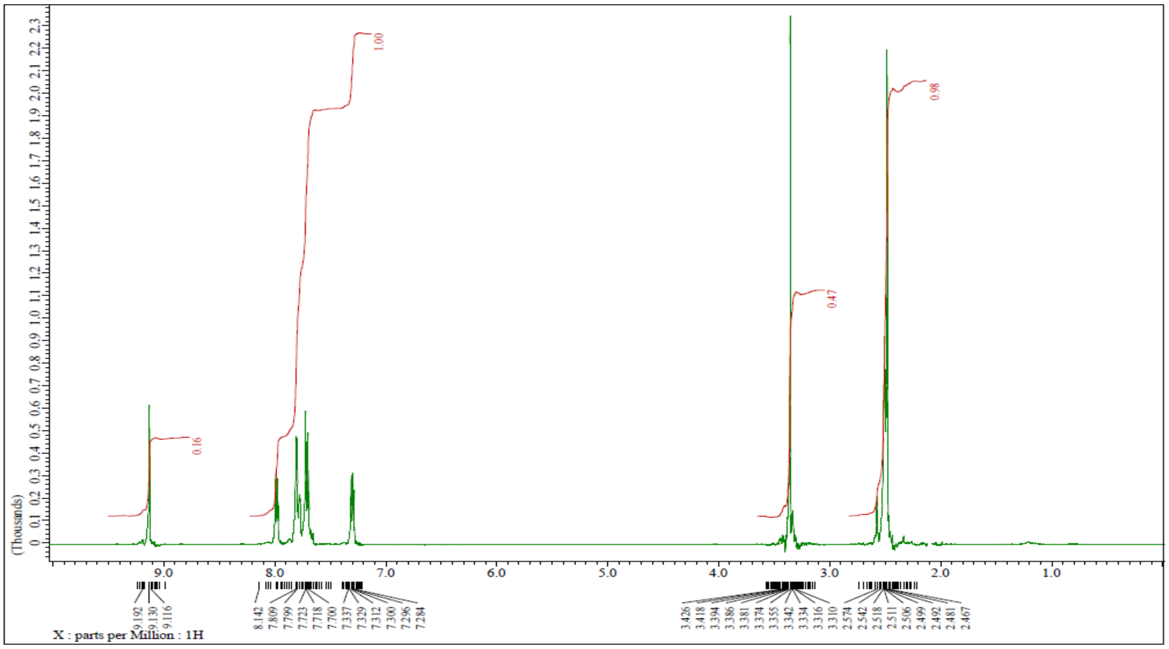

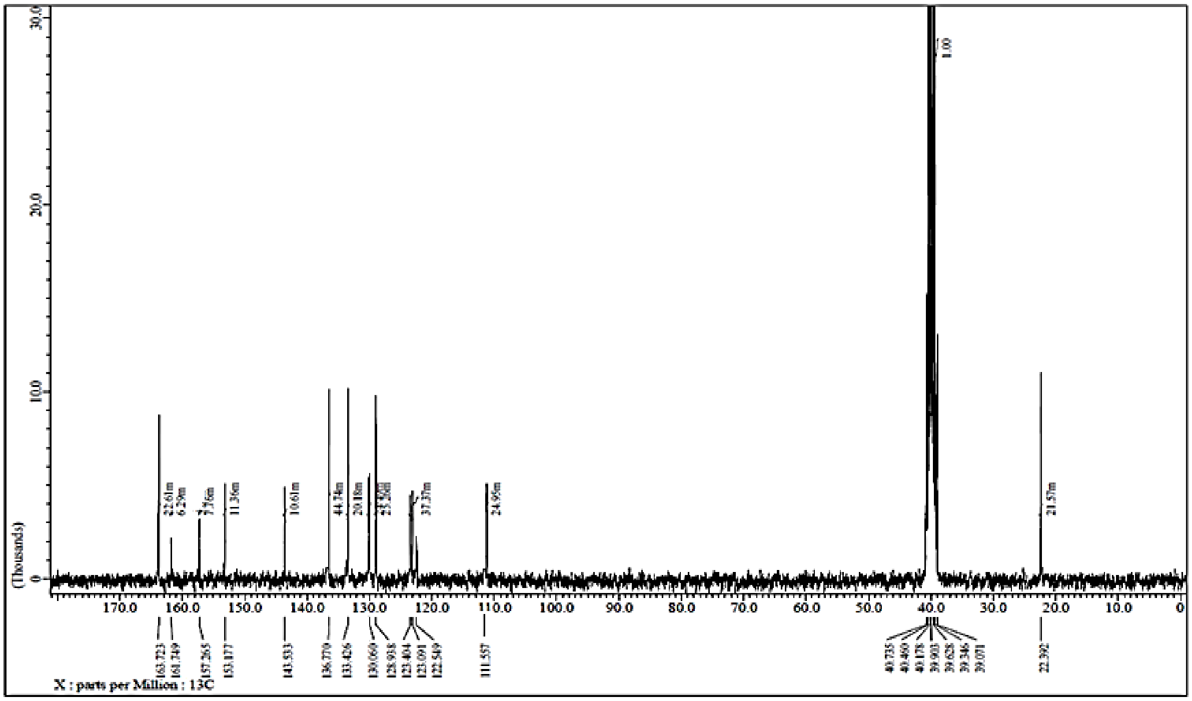

Compound 20 (DMSO-d_6)

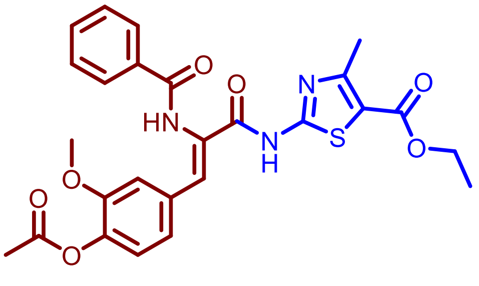

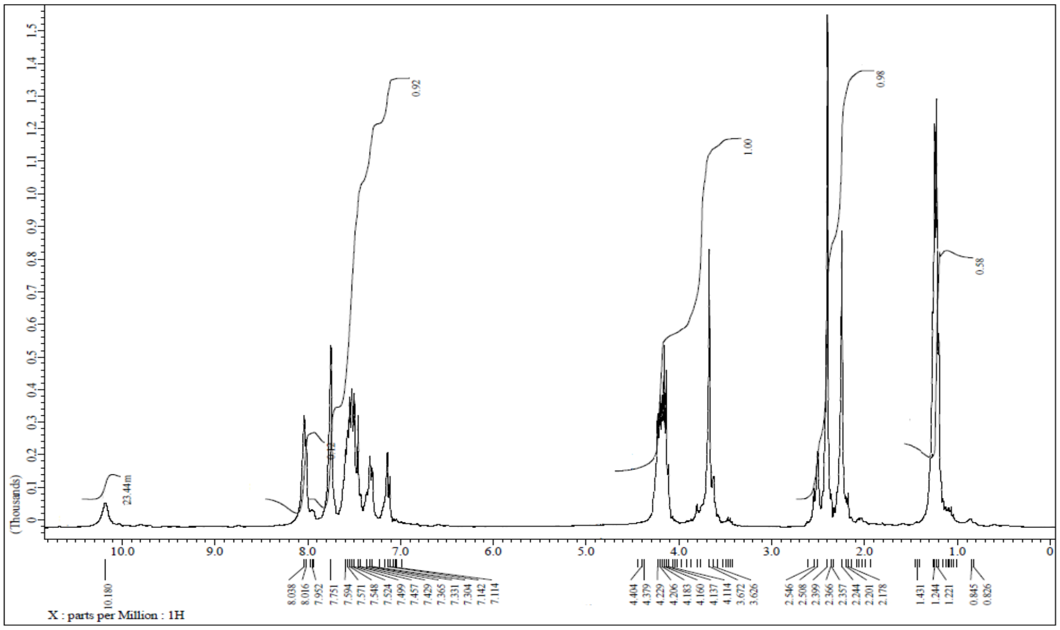

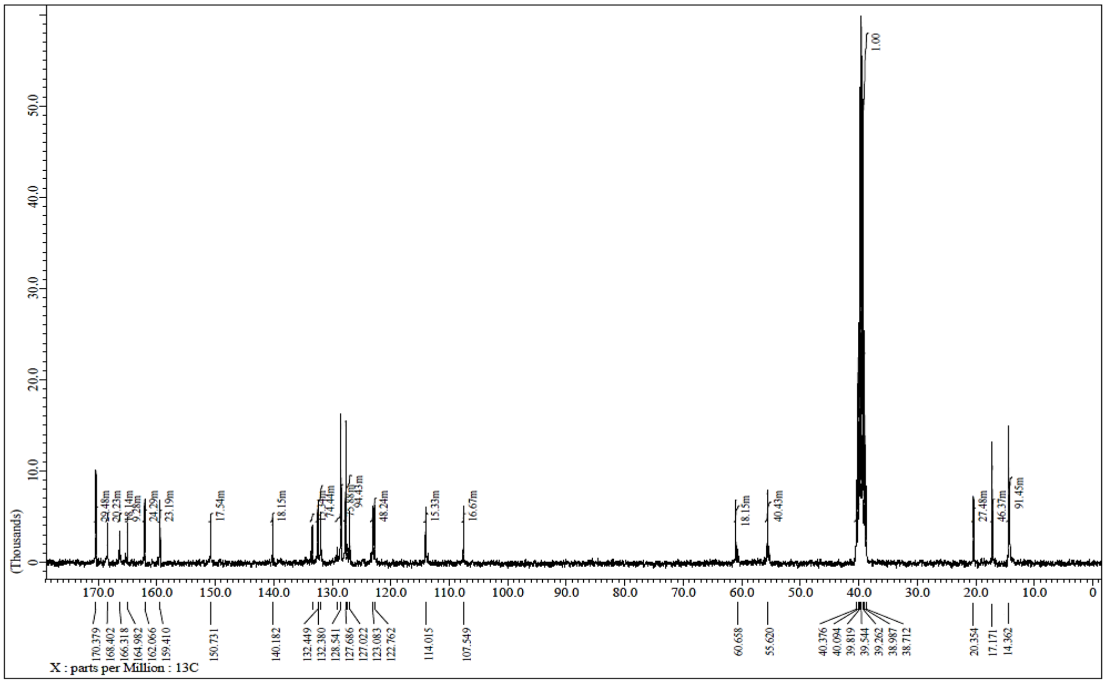

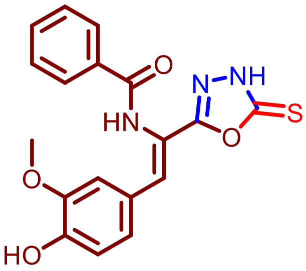

Compound 22 (DMSO-d_6)

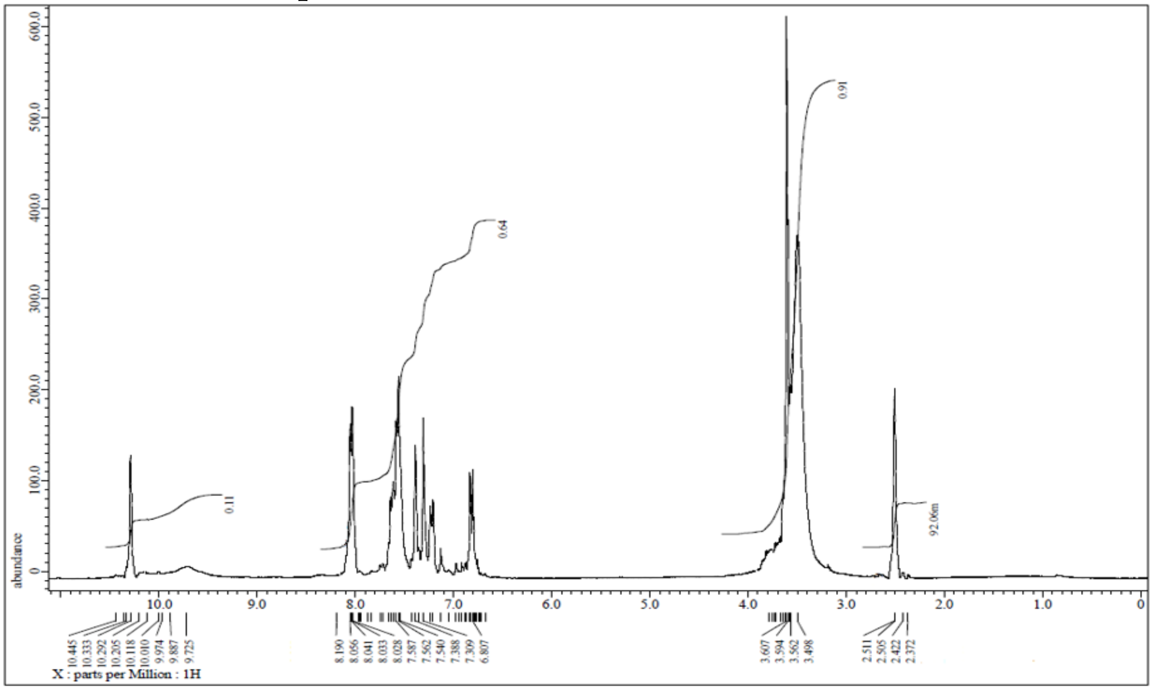

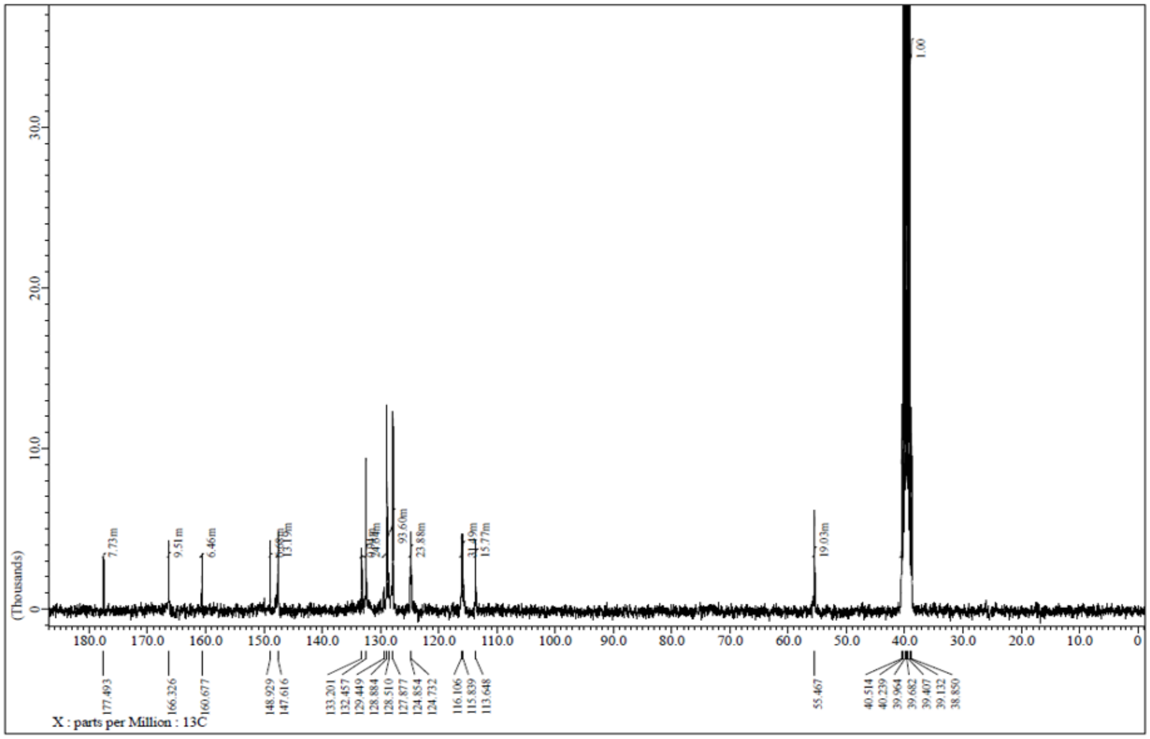

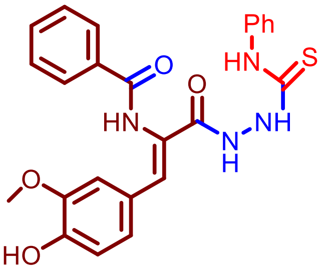

Compound 23 (DMSO-d_6)

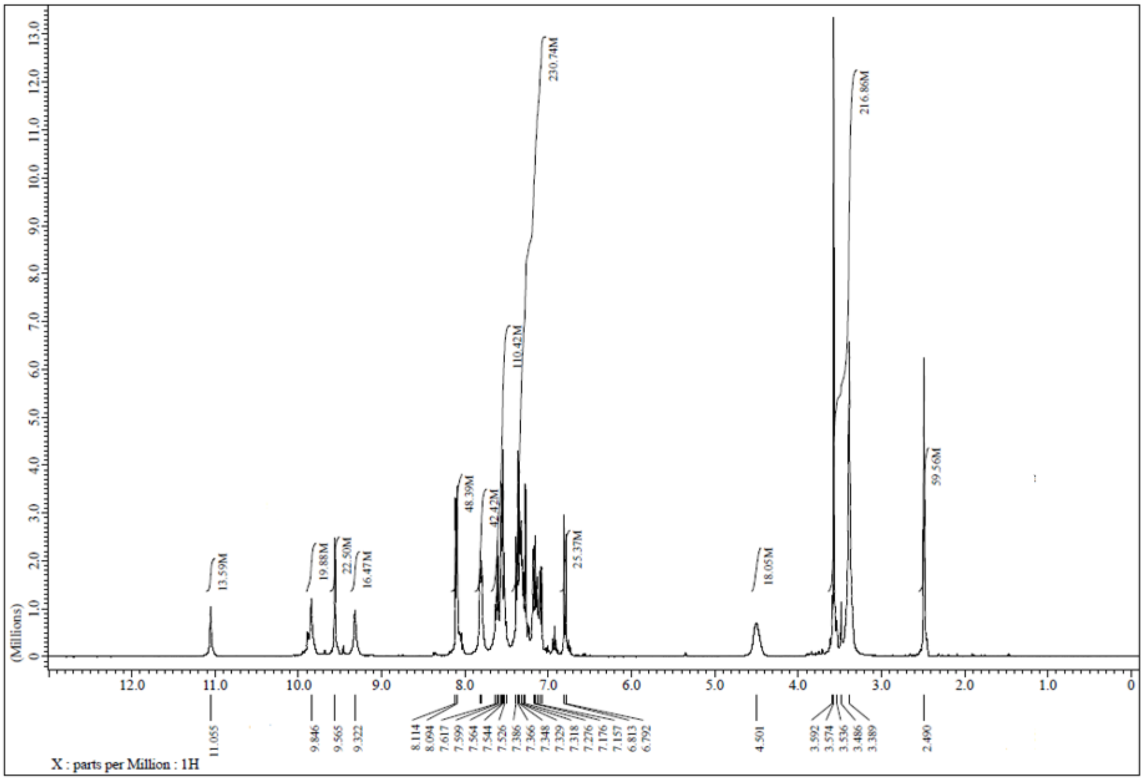

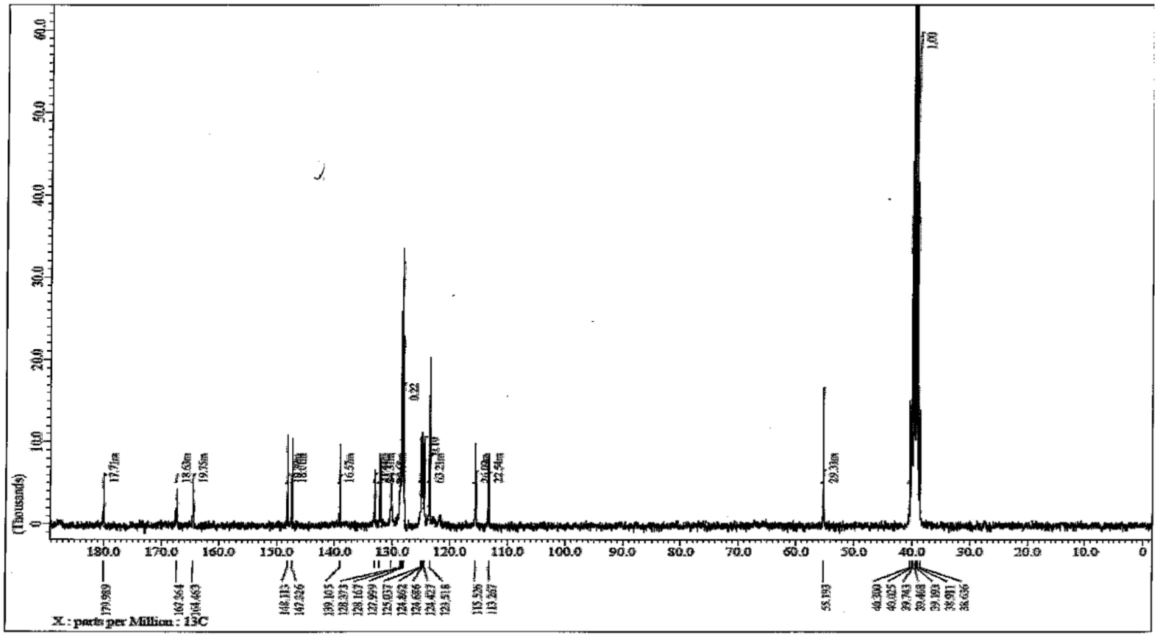

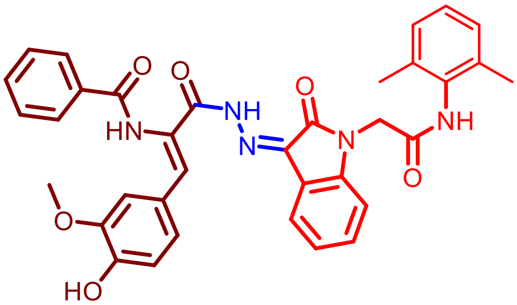

Compound 25 (DMSO-d_6)

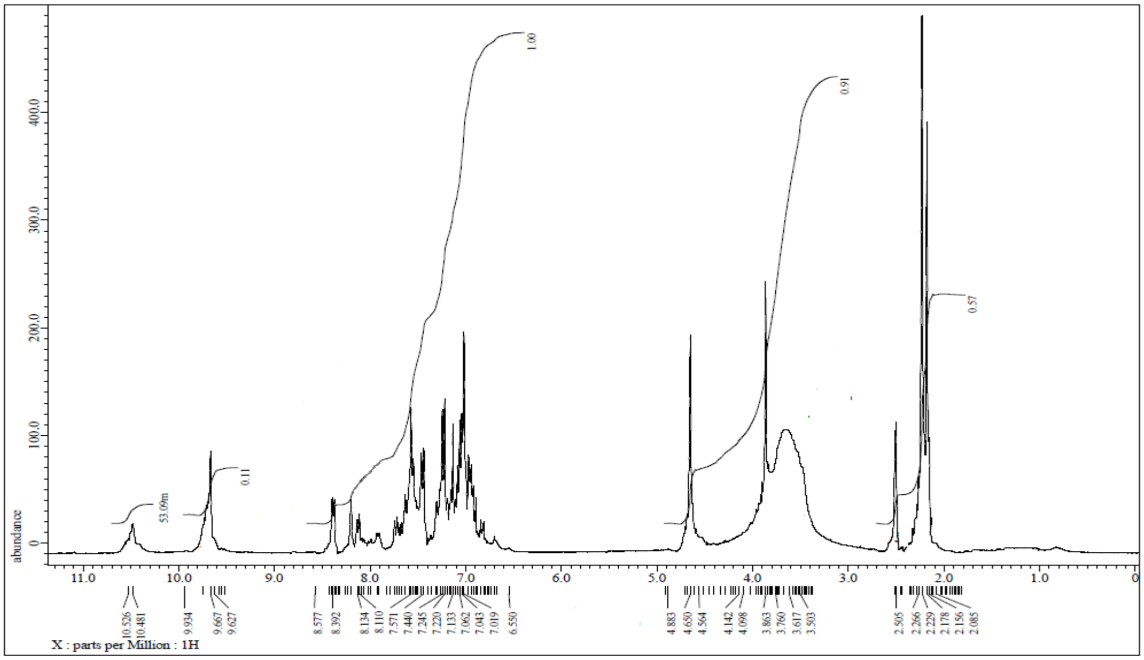

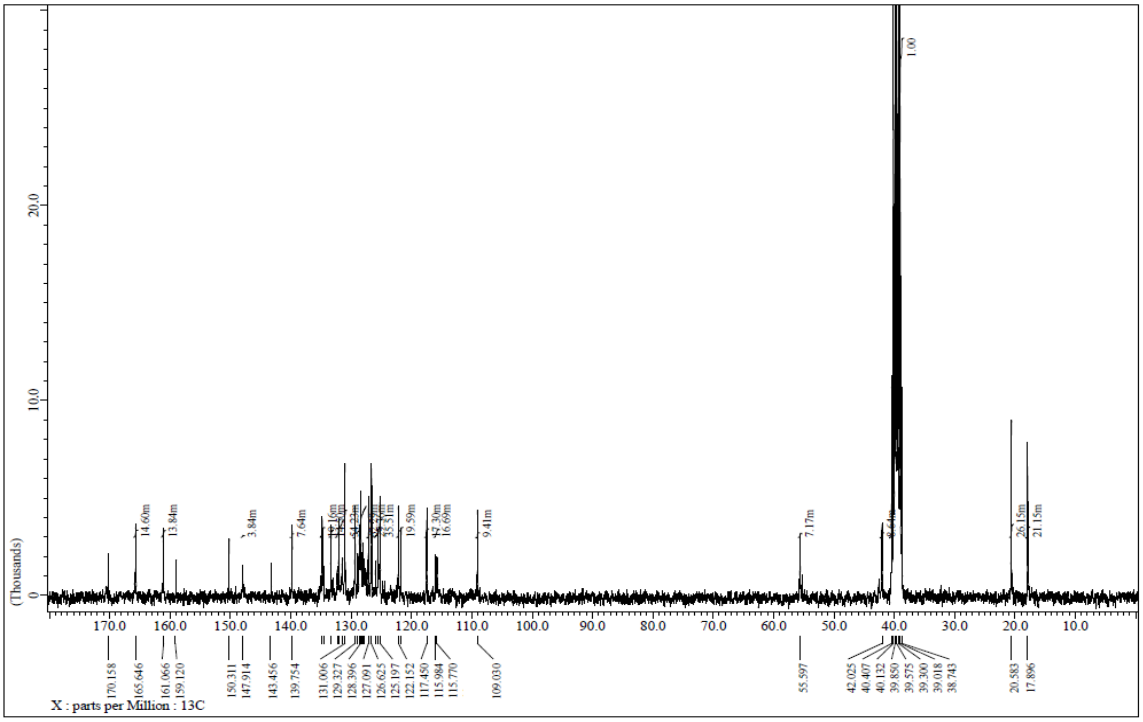

Supplement: Supplementary file 1 [file pharmaceuticals-18-00801-s001.zip › pharmaceuticals-3603449-supplementary.pdf]
